# Upper gut heat shock proteins HSP70 and GRP78 promote insulin resistance, hyperglycemia, and non-alcoholic steatohepatitis

**DOI:** 10.1038/s41467-022-35310-5

**Published:** 2022-12-13

**Authors:** Giulia Angelini, Lidia Castagneto-Gissey, Serenella Salinari, Alessandro Bertuzzi, Danila Anello, Meenakshi Pradhan, Marlen Zschätzsch, Paul Ritter, Carel W. Le Roux, Francesco Rubino, Nicola Basso, Giovanni Casella, Stefan R. Bornstein, Valentina Tremaroli, Geltrude Mingrone

**Affiliations:** 1 Università Cattolica del Sacro Cuore, Rome, Italy; 2 Fondazione Policlinico Universitario A. Gemelli IRCCS, Rome, Italy; 3 Department of Surgical Sciences, Sapienza University of Rome, Rome, Italy; 4 Department of Computer, Control, and Management Engineering “Antonio Ruberti”, University of Rome “Sapienza”, Rome, Italy; 5 CNR-Institute of Systems Analysis and Computer Science (IASI), Rome, Italy; 6 Wallenberg Laboratory for Cardiovascular and Metabolic Research, Department of Molecular and Clinical Medicine, University of Gothenburg, Gothenburg, Sweden; 7 Institute of Natural Materials Technology, Faculty of Mechanical Science and Engineering, Technischen Universität Dresden, Dresden, Germany; 8 Biotopa gGmbH, Dresden, Germany; 9 Bruker Daltonics SPR, Hamburg, Germany; 10 Diabetes Complications Research Centre, Conway Institute, University College Dublin, Dublin, Ireland; 11 Bariatric and Metabolic Surgery; King’s College Hospital, London, UK; 12 Department of Medicine III, Universitätsklinikum Carl Gustav Carus an der Technischen Universität Dresden, Dresden, Germany; 13 Division of Diabetes & Nutritional Sciences, School of Cardiovascular and Metabolic Medicine & Sciences, King’s College London, London, UK

**Keywords:** Endocrinology, Endocrine system and metabolic diseases

## Abstract

A high-fat diet increases the risk of insulin resistance, type-2 diabetes, and non-alcoholic steato-hepatitis. Here we identified two heat-shock proteins, Heat-Shock-Protein70 and Glucose-Regulated Protein78, which are increased in the jejunum of rats on a high-fat diet. We demonstrated a causal link between these proteins and hepatic and whole-body insulin-resistance, as well as the metabolic response to bariatric/metabolic surgery. Long-term continuous infusion of Heat-Shock-Protein70 and Glucose-Regulated Protein78 caused insulin-resistance, hyperglycemia, and non-alcoholic steato-hepatitis in rats on a chow diet, while in rats on a high-fat diet continuous infusion of monoclonal antibodies reversed these phenotypes, mimicking metabolic surgery. Infusion of these proteins or their antibodies was also associated with shifts in fecal microbiota composition. Serum levels of Heat-Shock-Protein70 and Glucose-Regulated Protein78were elevated in patients with non-alcoholic steato-hepatitis, but decreased following metabolic surgery. Understanding the intestinal regulation of metabolism may provide options to reverse metabolic diseases.

## Abstract (web summary)

High-fat diet (HFD) increases the risk of insulin resistance and non-alcoholic steatohepatitis (NASH). Here the authors report that the heat shock proteins HSP70 and GRP78 are increased in the jejunum of rats on HFD, and their continuous infusion leads insulin resistance and NASH, while in rats on a high-fat diet infused with monoclonal antibodies against HSP70 and GRP78 these phenotypes are reversed.

## Introduction

The prevalence of obesity and Type 2 Diabetes (T2D) has increased at epidemic rates over the last four decades, a trend that continues unabated today. Models predict that obesity will affect 50% of the US population by 2030; by the same year, the prevalence of diabetes will increase by 54% with over 54.9 million Americans expected to have diabetes^1,2^. Obesity and T2D share several pathophysiological features, including a state of low-grade chronic inflammation, hepatic and peripheral insulin resistance, ectopic fat deposition, and increased incidence of non-alcoholic fatty liver disease (NAFLD), including the most severe form of non-alcoholic steatohepatitis (NASH).

Metabolic surgery can induce durable (10-year and beyond) remission of T2D^3–7^, as well as reversal of chronic low-grade inflammation, hypertension^8^, early-stage chronic kidney disease^9^, and NAFL/NASH^10,11^. Clinical observations and mechanistic studies in animals^12–15^ and humans^16^ suggest that these effects cannot be entirely explained by weight loss. In fact, despite metabolic surgery often achieves complete clinical remission of obesity-related conditions, rarely does patients’ body mass index (BMI) become normal^3–7^. Moreover, certain surgical procedures that re-route the small intestine are more effective than others at improving insulin resistance, despite inducing similar weight loss^15–17^. In a recent study, Bilio-Pancreatic Diversion (BPD) and Roux-en-Y Gastric-Bypass (RYGB) induced identical weight loss but BPD resulted in greater improvement of insulin sensitivity^17^ and higher rates of diabetes remission^3^. In rodents, duodenal-jejunal bypass (DJB), an experimental procedure that excludes the proximal small bowel from nutrient transit, improves both diabetes^13,14^ and NASH^18^ independently of weight changes.

These observations support a direct effect of gastrointestinal procedures on mechanisms of metabolic regulation. Favorable changes in gut hormones^19^, bile acids^20^, and intestinal microbiota^21^ have been suggested to play a role in the improved metabolic regulation after gastrointestinal (GI) surgery. An alternative hypothesis (“anti-incretin theory”) suggests instead that GI signals induced by the transit of nutrients through the upper small bowel may cause insulin resistance^22^; accordingly, surgical exclusion of the duodenum and jejunum from nutrient transit might reduce such signals thus explaining diabetes reversal and other insulin-resistance states. Consistent with this hypothesis, glucose stimulation in a jejunal loop with intact vascular and nerve supply in swine models induces insulin resistance, whereas jejunectomy improves insulin sensitivity during gastric infusion of glucose^15^. These findings suggest that factors secreted in the jejunum may contribute to insulin resistance and metabolic disease, making the proximal gut a potential future therapeutic target.

We found that human jejunal mucosa secretes heat shock proteins (HSPs) in vitro, namely Heat Shock Protein-70 (HSP70) and the Glucose-Regulated Protein-78 (GRP78). Circulating levels of HSP70 were higher in people resistant to insulin than in healthy subjects and normalized after BPD^23^.

Here, we investigated the following hypotheses: (1) HSPs are secreted in the proximal small bowel in response to diets rich in fat; they negatively regulate insulin sensitivity and induce other pathophysiologic mechanisms of diabetes and NAFLD; (2) such proteins are reduced by DJB; (3) antibody neutralizing the activity of these proteins can recapitulate the beneficial metabolic effects of GI surgery.

## Results

### Rodent studies

#### Sites of intestinal heat shock proteins release

To identify intestinal regulators of insulin sensitivity, we used iTraQ labeling combined with nanoLC-MS/MS analysis to compare the jejunal secretome of four male rats feeding HFD vs. four male rats on chow diet (CD).

**Fig. 1:**
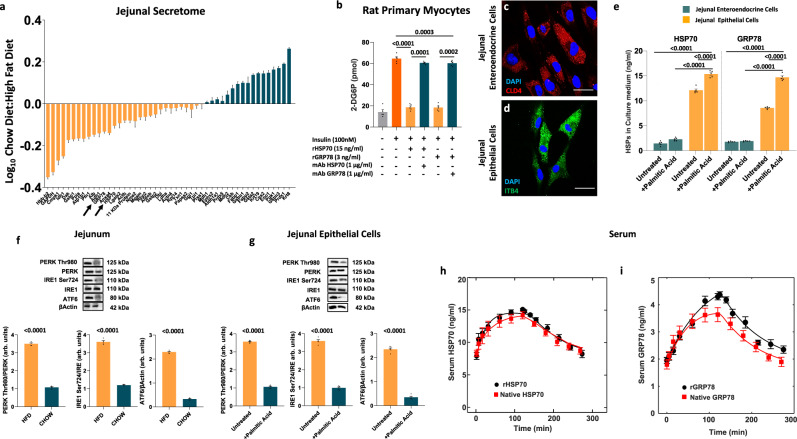
Sites of intestinal heat shock proteins release and their kinetics and action. **a** iTraQ-based analysis of proteins (*n* = 48) differentially expressed in the jejunal secretome in chow diet versus high-fat diet (*n* = 4 biologically independent animals). Downregulated proteins (*n* = 17) are colored in green while upregulated proteins (*n* = 31) are colored in orange. **b** Insulin-mediated glucose uptake of rat primary myocytes treated with HSP70 (15 ng/ml), GRP78 (3 ng/ml) alone or in combination with their respective mAbs (1 μg/ml). Rat myocytes were stimulated with 100 nM insulin for 10 min (*n* = 5 independent experiments). **c**, **d** Representative confocal microscopy images of FACS isolated rat enteroendocrine cells (**c**) stained for Claudin-4 (CLD4,red) and rat intestinal epithelial cells (**d**) stained for Integrin-β4 (ITB4,green) (*n* = 5 independent experiments). **e** HSP70 and GRP78 concentration in the culture medium of rat intestinal epithelial and enteroendocrine cells untreated or stimulated with 0.5 mM palmitic acid (*n* = 5 independent experiments). **f** Jejunal expression of key proteins involved in Unfolded Protein Response (UPR) in rats fed either chow diet (CD) or high-fat diet (HFD) (*n* = 5 biologically independent animals). **g** Expression of UPR proteins in rat intestinal epithelial cells untreated or stimulated with 0.5 mM palmitic acid (*n* = 5 independent experiments). **h**, **i** Time courses and fitting curves of recombinant (black circles) and native (red squares) HSP70 (**h**) and GRP78 (**i**) during and after a 2 h infusion of 2.5 ng/h of recombinant proteins and after an intragastric load of palm oil (*n* = 4 biologically independent animals). Magnification ×60. Scale bar: 50 μm Data are presented as mean value ± SEM. Statistical significances were calculated by unpaired two-tailed *t*-test and one-way Anova with Bonferroni’s correction for multiple comparisons, where appropriate. Source data are provided as a Source Data file.

We identified 17 and 31 proteins with higher and lower abundance, respectively, in the jejunal secretome of rats on HFD versus CD (Fig. 1a). To assess the potential to induce insulin resistance, we tested the secreted proteins in rat primary myocytes and followed their ability to affect insulin-mediated glucose uptake (Supplementary Fig. S1A). HSP70 and GRP78 drastically reduced glucose uptake mediated by insulin in myocytes, an effect counteracted by mAbs (Fig. 1b).

Next, we assessed HSP70 and GRP78 secretion in vitro from the duodenal, jejunal, and ileal mucosa of rats on HFD or CD. To exclude that HSPs release might be due to cell lysis after cell death, we assessed cell death by propidium iodide staining using flow cytometry (Supplementary Fig. S1B). HSP70 and GRP78 were secreted from all three intestinal locations, but the amounts secreted by the duodenum and jejunum were significantly higher in HFD compared to CD, with jejunum contributing the most (Supplementary Fig. S1C, D).

Consistent with these results, gene and protein expression of HSP70 and GPR78 increased in the jejunum of rats fed HFD compared with CD (Supplementary Fig. S1E–H).

To confirm that the release of HSP70 and GPR78 was dependent on lipid consumption, we assessed their serum levels in Zucker Diabetic Fatty (ZDF) rats, which are commonly used as research model of genetic obesity and T2D. Supporting our hypothesis, we observed lower serum levels of HSP70 and GPR78 in ZDF rats versus Wistar rats on HFD, while their levels were higher when compared with Wistar rats on CD (Supplementary Fig. S1I, J). The higher levels of circulating HSPs observed in ZDF rats versus Wistar rats on CD may depend on the concomitant obesity and, thus, on the higher fat mass of the former. We also observed a drastic decrease in serum levels of HSP70 and GPR78 after DJB as compared with sham-operated rats (Supplementary Fig. S1K, L).

Therefore, our results suggest that diet modulated the levels of HSP70 and GPR78 in both the intestine and circulatory stream, while the jejunum was the major site of gut mucosal secretion during HFD.

#### Gut cellular types secreting heat shock proteins

To understand the cellular source of dietary-induced HSP70 and GRP78, intestinal epithelial and enteroendocrine cells were isolated from the jejunum using Fluorescent Activated Cell Sorting (FACS) (Supplementary Fig. S2A, B). Sorted claudin-4 epithelial cells in the small intestine express in an exclusive manner the chromogranin A gene (Chga) and other enteroendocrine cell-related genes (Ffar1, Ffar4, Gpr119), thus are assumed to be enteroendocrine cells^24^ (Fig. 1c). Epithelial cells were tagged with integrin-β4 that is a specific marker of gut epithelial cells (Fig. 1d). Enteroendocrine or epithelial cells were then incubated in vitro with 0.5 mM palmitic acid for 24 h and the cell medium collected. HSP70 and GRP78 were measured in the medium by ELISA. Figure 1e shows it is epithelial cells that secrete HSP70 and GRP78 when stimulated with a lipid load.

#### Endoplasmic reticulum stress and unfolded protein response promote HSP70 and GRP78 synthesis and secretion

To assess if HSP70 and GRP78 synthesis and secretion were promoted by HFD-induced Endoplasmic Reticulum (ER) stress and Unfolded Protein Response (UPR) activation, we measured the three UPR activator proteins, Inositol-requiring enzyme 1 (IRE1), PKR–like endoplasmic reticulum kinase (PERK), and activating transcription factor-6 (ATF6).

Prolonged HFD promoted ER stress of the jejunal epithelium increasing the phosphorylation of IRE1 Ser724 and PERK Thr980 and the expression of ATF6 (Fig. 1f).

Next, we assessed UPR activation in rat gut epithelial cells incubated in vitro with 0.5 mM palmitic acid for 24 h. Consistent with the in vivo results, epithelial cells stimulated with palmitic acid overload showed increased phosphorylation of IRE1 Ser724 and PERK Thr980 as well as a higher expression of ATF6 (Fig. 1g).

#### Recombinant and serum HSP70 and GRP78 kinetics

To compute the kinetics of HSP70 and GRP78 recombinant proteins in the circulatory stream and compare to native proteins, HSP70 and GRP78 serum levels were measured before and at various time points after starting a 2 h infusion of the recombinant HSP70 or GRP78 proteins via a subcutaneous osmotic pump and after stopping it, as well as after an intragastric load of palm oil (Fig. 1h, i).

The serum clearance of both HSP70 and GRP78 either exogenous or endogenous was well predicted by a nonlinear Michaelis-Menten kinetics suggesting a clearance pathway mediated by receptor binding and disposition. The half-life of recombinant and rat native HSPs was about 60 min. The kinetic parameters are reported in the Supplementary Table S1.

#### Pharmacokinetics of mAbs

Validation of mAb binding to native serum HSP70 or GRP78 by Surface Plasmon Resonance and co-immunoprecipitation are reported in the Supplemental Material (Supplementary Fig. S3A–H).

We studied mAb pharmacokinetics using a previously validated method based on fluorescence intensity to measure antibody concentrations^25^.

To assess the kinetics of mAbs, we injected a bolus of 1 µg of anti-HSP70 or anti-GRP78 mAbs subcutaneously in three male rats. The volume of distribution of HSP70 estimated by the model was 44 ml for HSP70 and 51 ml for GRP78 (see Supplementary Fig. S4A, B).

The estimates of the quasi-steady-state dissociation constant, K_SS_, were larger than the measured K_D_ values (752 pM for HSP70 and 608 pM for GRP78), which agrees with values of internalization into tissues (k_int_) close to the elimination constant (k_e_) and suggests that internalization of bound mAbs into tissues may occur with a rate similar to the elimination of free mAbs from serum (see Supplementary Fig. S4A, B).

The mAb half-life, measured from the descending branch of the concentration curve, was 1.68 h for HSP70 and 1.89 h for GRP78.

It has been suggested that delayed absorption depending on prolonged duration at the subcutaneous injection site may increase local catabolism of mAb with subsequent impact on bioavailability^26^. For this reason, we opted for a continuous infusion instead of daily injections.

Then, we assessed the capacity of each mAb to counteract the action of the relative recombinant protein. As shown in Fig. 1b, 1 µg/ml concentration of both mAbs was able to abolish the inhibitory effect of HSP70 or GRP78 on glucose uptake in myocyte cultures.

Based on the affinity of mAbs for the recombinant proteins, on the kinetics data and on the counteracting effects on glucose uptake in isolated myocytes, we decided to continuously infuse 1 µg/h of each mAb subcutaneously.

#### Organ distribution of mAbs

We then aimed to follow the fate of injected mAbs and observe tissue distribution. Rats were anesthetized, injected subcutaneously with 1 mg/ml of Alexa Fluor 488 labeled HSP70 or GRP78 or isotype control and organs scanned in a Fluorescence Imaging system (IVIS Lumina 3, PerkinElmer, Waltham, MA)^27^.

Fluorescence imaging of major organs revealed that the majority of labeled mAbs was present primarily in duodenum and jejunum, followed by kidney and liver, within 2 h of injection (Supplementary Fig. S5A–F).

There are conflicting opinions regarding whether the central pathway of absorption of macromolecules from the subcutaneous space is via lymphatic transport or through a more direct entry into vascular capillaries, although the latter seems most likely^28^.

Once in the circulatory stream, mAbs may distribute through interstitial fluid via diffusion and convection and may be internalized by receptor-mediated endocytosis or by fluid-phase endocytosis and then undergo proteolytic catabolism.

A specific clearance of a mAb is mediated by its interaction with target antigens and, therefore, its accumulation is a function of the expression of its target in tissues^29^, showing that the primary targets are the jejunum and duodenum, followed by the liver.

#### Infusion of HSP70 and GRP78 in rats under chow diet

To study the role of HSPs in the onset of hepatic and peripheral insulin resistance, we implanted subcutaneous osmotic pumps filled with recombinant HSP70 or GRP78 proteins in male rats fed a CD. The infusion rates of recombinant proteins dissolved in saline were set to match the serum concentrations in HFD (Supplementary Fig. S6A). Rats infused with saline solution were included as controls.

**Fig. 2:**
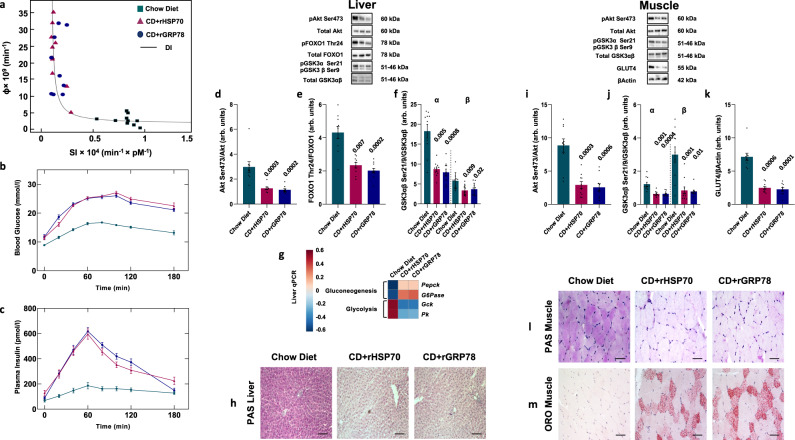
Effects of continuous infusion of HSP70/GRP78 recombinant proteins. **a** Glucose disposition index (DI) metrics showing experimental data and fitting curves. Total β-cell responsivity to glucose (Φ) is a function of whole-body insulin sensitivity (SI). **b**, **c** Time courses of blood glucose and plasma insulin concentrations during an oral glucose tolerance test. **d**–**f** Hepatic phosphorylation of Akt on Ser473 (**d**), FoxO1 on Thr24 (**e**), and GSK3ab on Ser21/9 (**f**). **g** Gene expression of key enzymes involved in hepatic gluconeogenesis and glycolysis. **h** Representative images showing periodic acid Schiff (PAS) staining of liver section from rats fed chow diet (CD) and infused with recombinant HSP70 or GRP78 or saline solution. **i**–**k** Western blot analysis of Akt Ser473 (**i**) and GSK3αβ Ser21/9 (**j**) phosphorylation and GLUT4 (**k**) expression in skeletal muscle. **l**, **m** Representative images showing PAS (**l**) and Oil red O (ORO) (**m**) staining of skeletal muscle sections from rats fed CD and infused with recombinant HSP70 or GRP78 or saline solution. Magnification ×20. Scale bar: 0.10 mm. Data are presented as mean value ± SEM of *n* = 10 biologically independent animals. Statistical significances were calculated by Kruskal–Wallis test with Dunnett’s correction for multiple comparisons. Source data are provided as a Source Data file.

On a standard CD, age-associated metabolic syndrome and obesity require one full year to develop^30^. However, rats with continuous infusion of HSP70 or GRP78 rapidly developed obesity although remaining on a CD (Supplementary Fig. S6B). Total body weight, the weight of the visceral adipose tissue (VAT), plasma total bile acids, alanine transaminase (ALT), aspartate aminotransferase (AST), cholesterol (CH), and triglycerides (TG) increased compared to controls while subcutaneous adipose tissue (SAT) and liver weight were unaffected. We observed no significant changes in food intake and no differences in the plasma levels of glucagon-like peptide-1 (GLP-1) and Peptide YY (PYY) (Supplementary Fig. S6C–M) suggesting a decreased energy expenditure. Moreover, whole body and hepatic insulin resistance with compensatory increased insulin secretion occurred (Fig. 2a–c Supplementary Table S2).

To investigate the effects of HSP70 or GRP78 on insulin signaling, we assessed the expression of proteins involved in insulin signaling and genes coding for key rate-limiting enzymes of gluconeogenesis and glycogen synthesis in the liver and the levels of proteins involved in insulin signaling and glucose uptake in skeletal muscle. Infusion of HSP70 or GRP78 suppressed hepatic Akt Ser473 and Foxo1 Thr24 phosphorylation (Fig. 2d, e), resulting in a higher expression of gluconeogenic genes leading to increased glucose production and reduced glycolysis (Fig. 2g). In addition, lower GSK-3αβ Ser 21/9 phosphorylation (Fig. 2f) resulted in a decreased hepatic glycogen storage (Fig. 2h). In line with the observed whole-body insulin resistance, Akt and GSK-3αβ phosphorylation were reduced in the skeletal muscle tissue by HSP70 or GRP78 infusion (Fig. 2i, j), resulting in reduced GLUT4 expression and glycogen deposition (Fig. 2k, l).

Furthermore, the infusion of recombinant proteins increased ectopic fat accumulation in the skeletal muscle (Fig. 2m). Thus, these results indicate that rats continuously infused with HSP70 or GRP78 develop an obese and insulin-resistant phenotype similar to the one induced by a HFD.

**Fig. 3:**
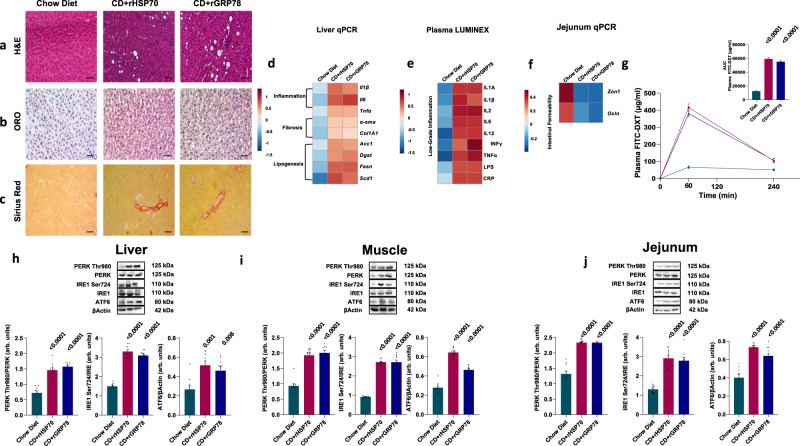
Effects of continuous infusion of HSP70 or GRP78 on NASH hallmarks. **a**–**c** Representative Hematoxylin and Eosin (H&E) (**a**), Oil Red O (ORO) (**b**), and Picro Sirius Red (**c**) staining of liver sections from rats under CD and infused with recombinant HSP70 or GRP78 or saline solution. **d** Gene expression of liver inflammation and fibrosis markers as well as key enzymes involved in hepatic de novo lipogenesis. **e** Plasma low-grade inflammation markers assessed by Luminex. **f** Gene expression of key genes involved in intestinal permeability. **g** Time course and AUC of in vivo intestinal permeability assessed by measuring Fluorescein IsoThioCyanate Dextran 4000 Da (FITC-DXT) in the systemic circulation after an oral gavage with FITC-DXT (500 mg/kg body weight). **h**–**j** Hepatic (**h**), skeletal muscle (**i**), and jejunal (**j**) expression of key proteins involved in Unfolded Protein Response (UPR). Magnification ×20. Scale bar: 0.10 mm. Data are presented as mean value ± SEM of *n* = 10 biologically independent animals. Statistical significances were calculated by one-way Anova with Bonferroni’s correction for multiple comparisons. Source data are provided as a Source Data file.

Obesity is associated with a low-grade inflammation that contributes to the development of complications such as NAFLD, which is recognized as the hepatic manifestation of metabolic syndrome^31^. To determine if HSP70 or GRP78 infusion was also associated with NASH, a more aggressive form of NAFLD in which inflammation associates with liver steatosis^32^, we performed histological analysis and assessed genes and proteins linked to NASH. Compared with saline, rats fed a CD and infused with HSP70 or GRP78 in saline solution showed an increase in hepatic steatosis and portal and lobular inflammation as well as fibrosis (Fig. 3a–c). Consistently, we also observed higher expression of genes involved in inflammation, fibrosis, and lipogenesis (Fig. 3d), increased expression of pro-inflammatory cytokines (IL1A and IL1B, IL2, IL6, IL12, INFγ, TNFα), and increased circulation of bacterial endotoxin lipopolysaccharides (LPS) and C-reactive protein (CRP) (Fig. 3e).

Augmented intestinal permeability is associated with increased liver fat and visceral adiposity, and is a likely main driver of increased LPS circulation and systemic low-grade inflammation^33^. Therefore, we investigated the expression of genes involved in the regulation of intestinal permeability. Rats infused with HSP70 or GRP78 had reduced gene expression of zonulin and occludin, coding for proteins present in epithelial tight junction (Fig. 3f). The increased intestinal permeability as compared with normal rats was confirmed by intragastric gavage with Fluorescein IsoThiocyanate Dextran 4000 Da (FITC-DXT) showing significantly higher FITC-DXT in blood during 4 h after gavage (Fig. 3g).

We then examined whether HSP70 and GRP78 could contribute to intestinal inflammation through toll-like receptor-4 (TLR4). We incubated primary rat colon intestinal epithelial cells with HSP70 or GRP78 in the presence or absence of TAK242, a small molecule that inhibits TLR4^34^, to assess key genes involved in inflammation and intestinal permeability. We showed that in vitro incubation with HSP70 or GRP78 activated TLR4 signaling cascade and increased the expression of genes implicated in the intestinal permeability (Supplementary Fig. S7A–C), while treatment with TAK242 abrogated these effects.

To elucidate the mechanism of action of HSP70 and GRP78 we investigated if these HSPs can activate TLR4 signaling and trigger endoplasmic reticulum (ER) stress in target cells.

In vitro stimulation of rat primary hepatocytes with HSP70 or GRP78 induced cytoplasmic accumulation of triglycerides into lipid droplets due to simultaneous increase of free fatty acid uptake and fatty acid synthesis (Supplementary Fig. S7D–I). Therefore, HSP70 and GRP78 cause liver steatosis.

HSP70 stimulates immune and inflammatory responses^35^. We show that in vitro incubation with HSP70 or GRP78 activated hepatic stellate cells (HSC) that acquired myofibroblast features, including expression of smooth muscle α-actin, tumor growth factor beta (TGF-β) and production of collagen (Supplementary Fig. S7J–O). This translates into the liver fibrosis observed after continuous infusion of these proteins in vivo.

Both rat hepatocytes and HSCs express TLR4 and proteins belonging to the HSP70 family bind TLR4^36^. During our in vitro experiments, the content of LPS was <1 EU/µg of recombinant protein, but we sought to neutralize possible traces of endotoxin in the HSP70 or GRP78 preparations with Polymyxin B. We wanted to verify whether blocking TLR4 would reduce the effects of HSP70 or GRP78. TAK-242 prevented the effects of HSP70 or GRP78 on lipid accumulation in hepatocytes as well as the transition of HSC to myofibroblast phenotype.

Progression from simple steatosis to NASH may depend on ER stress^37^. Thus, we measured IRE1 Ser724, PERK Thr980 phosphorylation, and ATF6 expression in liver and skeletal muscle. In line with our hypothesis, the phosphorylation of IRE1 Ser724 and PERK Thr980 and the expression of ATF6 was increased in the liver and the skeletal muscle by HSP70 or GRP78 continuous infusion (Fig. 3h–j).

Our in vitro experiments, performed in rat primary hepatocytes HSCs and myocytes (Supplementary Fig. S8A–C), confirmed that HSP70 and GRP78 are responsible for ER stress and the activation of UPR in target cells.

Bile acids play a role in the regulation of glucose metabolism enhancing AKT activity and reducing hepatic gluconeogenesis^38^. Thus, we measured Akt Ser473 and Foxo1 Thr24 phosphorylation in rat primary hepatocytes and glucose uptake in rat primary myocytes, treated with recombinant HSP70 or GRP78, in the presence or absence of taurocholate.

Taurocholate treatment, partially reverted the insulin-resistance effects of HSP70 or GRP78 in hepatocytes and myocytes, suggesting other possible mechanisms (Supplementary Fig. S8D, E), such as TLR4 and ER stress activation.

Taken together, our data supports the role of HSP70 and GRP78 in the development of a NASH phenotype comparable to the one induced by prolonged HFD feeding^39^.

#### Infusion of mAbs against HSP70 and GRP78 in rats under HFD

To test the hypothesis that HSP70 and GRP78 are necessary for the development of insulin resistance and NASH, we infused antibodies in saline to block circulating HSP70 or GRP78 in male rats with an established phenotype of NASH caused by prolonged HFD feeding (i.e., 16 weeks). We found a significant reduction of both HSP70 and GRP78 circulating levels following mAb infusion (Supplementary Fig. S9A) suggesting blockade of the circulating and possibly the jejunal targets.

**Fig. 4:**
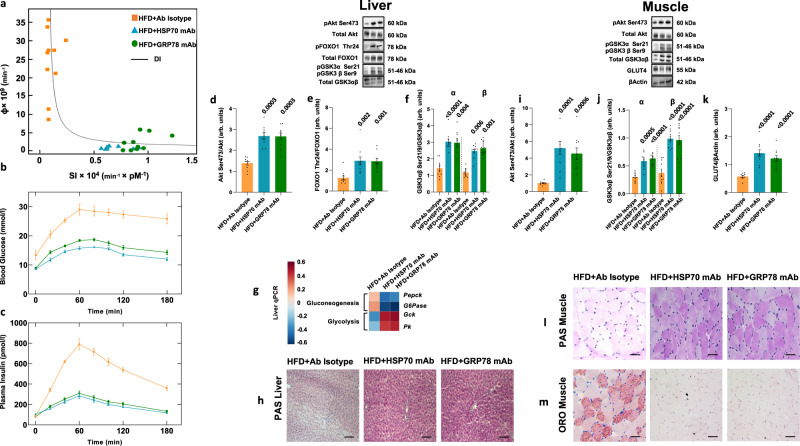
Effects of native HSP70/GRP78 inhibition with mAbs on insulin sensitivity. **a** Glucose disposition index (DI) metrics showing experimental data and fitting curves. Total β-cell responsivity to glucose (Φ) is a function of whole-body insulin sensitivity (SI). **b**, **c** Time courses of blood glucose and plasma insulin concentrations during an oral glucose tolerance test. **d**–**f** Hepatic phosphorylation of Akt on Ser473 (**d**), FoxO1 on Thr24 (**e**), and GSK3ab on Ser21/9 (**f**). **g** Gene expression of key enzymes involved in hepatic gluconeogenesis and glycolysis. **h** Representative images showing periodic acid Schiff (PAS) staining of liver section from rats fed HFD and infused with monoclonal antibodies against HSP70 or GRP78 or antibody isotype. **i**–**k** Western blot analysis of Akt Ser473 (**i**) and GSK3αβ Ser21/9 (**j**) phosphorylation and GLUT4 (**k**) expression in skeletal muscle. **l**, **m** Representative images showing PAS (**l**) and Oil red O (ORO) (**m**) staining of skeletal muscle sections from rats fed HFD and infused with monoclonal antibodies against HSP70 or GRP78 or antibody isotype. Magnification ×20. Scale bar: 0.10 mm. Data are presented as mean value ± SEM of *n* = 10 biologically independent animals. Statistical significances were calculated by Kruskal–Wallis test with Dunnett’s correction for multiple comparisons. Source data are provided as a Source Data file.

Rats infused with isotype control antibodies were included as controls. We observed reduced total body weight time course, VAT, plasma total bile acids, ALT, AST, CH, and TG levels following antibodies infusion compared to isotype-infused controls while SAT depots and liver weight were not affected. We observed no significant changes in food intake and no differences in the plasma levels of GLP-1 and PYY (Supplementary Fig. S9B–M) suggesting an increased energy expenditure. Moreover, hepatic and whole-body insulin sensitivity as well as disposition index increased, while insulin secretion decreased (Fig. 4a–c, Supplementary Table S2), suggesting that the antibody infusions were able to counteract the effect of a prolonged HFD.

The infusion of blocking antibodies increased hepatic Akt Ser473 and Foxo1 Thr24 phosphorylation (Fig. 4d, e) resulting in a lower expression of hepatic gluconeogenic genes (Fig. 4g).

Moreover, higher GSK-3αβ Ser 21/9 phosphorylation (Fig. 4f) led to an increased glycogen storage in the liver (Fig. 4h). In the skeletal muscle, increased Akt Ser473 and GSK-3αβ Ser21/9 phosphorylation (Fig. 4i, j) contributed to a higher expression of GLUT4 and glycogen storage (Fig. 4k, l). Besides, the infusion of blocking antibodies reduced ectopic fat accumulation in the skeletal muscle (Fig. 4m). These results indicated a positive effect of mAbs on insulin signaling.

**Fig. 5:**
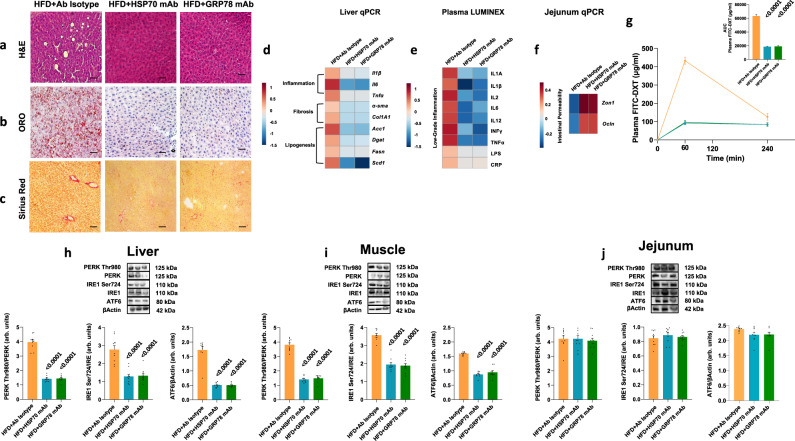
Effects of native HSP70/GRP78 inhibition with mAbs on NASH hallmarks. **a**–**c** Representative Hematoxylin and Eosin (H&E) (**a**), Oil Red O (ORO) (**b**), and Picro Sirius Red (c) staining of liver sections from rats fed HFD and infused with monoclonal antibodies against HSP70 or GRP78 or antibody isotype. **d** Gene expression of liver inflammation and fibrosis markers as well as key enzymes involved in hepatic de novo lipogenesis. **e** Plasma low-grade inflammation markers assessed by Luminex. **f** Gene expression of key genes involved in intestinal permeability. **g** Time course and AUC of in vivo intestinal permeability assessed by measuring Fluorescein IsoThioCyanate Dextran 4000 Da (FITC-DXT) in the systemic circulation after an oral gavage of FITC-DXT (500 mg/kg body weight). **h**–**j**, Hepatic (**h**), skeletal muscle (**i**), and jejunal (**j**) expression of key proteins involved in Unfolded Protein Response (UPR). Magnification ×20. Scale bar: 0.10 mm. Data are presented as mean value ± SEM of *n* = 10 biologically independent animals. Statistical significances were calculated by one-way Anova with Bonferroni’s correction for multiple comparisons. Source data are provided as a Source Data file.

Histological analysis in the liver for markers of NASH showed that the infusion of mAbs decreased hepatic steatosis, inflammation, and fibrosis (Fig. 5a–c). Genes involved in inflammation, fibrosis, and lipogenesis were also decreased (Fig. 5d), while levels of inflammatory cytokines, LPS and CRP in circulation were lower (Fig. 5e). Therefore, as expected, intestinal zonulin and occludin gene expression increased while plasma FITC-DXT decreased after antibody infusions (Fig. 5f, g), suggesting a reduction of intestinal permeability. Moreover, the phosphorylation of IRE1 Ser724 and PERK Thr980 and the expression of ATF6 was decreased in both liver and skeletal muscle by the infusion of blocking antibodies against HSP70 or GRP78 continuous infusion (Fig. 5h–j)

Overall, these results indicate that the infusion of antibodies, which block HSP70 or GRP78, reversed the histological and molecular features of NASH, potentially mimicking the effects of DJB.

#### Infusion of HSP70 and GRP78 in DJB

To ascertain the role of HSP70 and GRP78 in the onset of hepatic and peripheral insulin resistance and NASH pathogenesis, we used osmotic pumps to continuously infuse recombinant HSP70 or GRP78 in saline or saline solution in rats feeding a HFD after a DJB. For the control group, we included sham-operated rats implanted with osmotic pumps filled with saline solution.

**Fig. 6:**
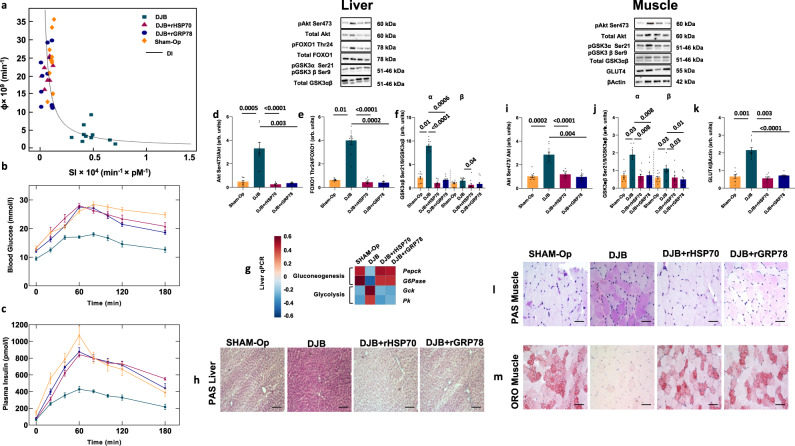
Effects of HSP70 or GRP78 infusion in Duodenal-Jejunal Bypass (DJB) on insulin sensitivity. **a** Glucose disposition index (DI) metrics showing experimental data and fitting curves. Total β-cell responsivity to glucose (Φ) is a function of whole-body insulin sensitivity (SI). **b**, **c** Time courses of blood glucose and plasma insulin concentrations during an oral glucose tolerance test. **d**–**f** Hepatic phosphorylation of Akt on Ser473 (**d**), FoxO1 on Thr24 (**e**), and GSK3ab on Ser21/9 (**f**). **g** Gene expression of key enzymes involved in hepatic gluconeogenesis and glycolysis. **h** Representative images showing periodic acid Schiff (PAS) staining of liver section from rats fed HFD that underwent Sham-operation or DJB and infused with recombinant HSP70 or GRP78 or saline solution. **i**–**k** Western blot analysis of Akt Ser473 (**i**) and GSK3αβ Ser21/9 (**j**) phosphorylation and GLUT4 (**k**) expression in skeletal muscle samples. **l**, **m** Representative images showing PAS (**l**) and (ORO) (**m**) staining of skeletal muscle sections from rats fed HFD that underwent Sham-operation or DJB and infused with recombinant HSP70 or GRP78 or saline solution. Magnification ×20. Scale bar: 0.10 mm Data are presented as mean value ± SEM of *n* = 10 biologically independent animals. Statistical significances were calculated by Kruskal–Wallis test with Dunnett’s correction for multiple comparisons. Source data are provided as a Source Data file.

Rats after a DJB, infused with saline solution, maintained the effects of metabolic surgery on body weight and composition, while the DJB group infused with HSP70 or GRP78 reverted to an obese phenotype similar to that of the sham-operated rats (Supplementary Fig. S10A–I). We observed no significant changes in food intake while the plasma levels of GLP-1 and PYY were significantly increased only in the DJB group (Supplementary Fig. S10J–L). Compared with the sham group, blood glucose increased in response to an oral glucose load in DJB rats infused with HSP70 or GRP78, while hepatic and whole-body insulin sensitivity decreased (Fig. 6a–c, Supplementary Table S2). Accordingly, infusion of HSP70 or GRP78 in the DJB group suppressed liver Akt Ser473 and Foxo1 Thr24 phosphorylation (Fig. 6d, e) resulting in a higher expression of gluconeogenic genes (Fig. 6g). In addition, this group displayed low GSK-3αβ Ser 21/9 phosphorylation (Fig. 6f) and decreased hepatic glycogen storage (Fig. 6h). Infusion of HSP70 or GRP78 reduced Akt Ser473, GSK-3αβ Ser21/9 phosphorylation, and GLUT4 expression in the skeletal muscle (Fig. 6i–k). Moreover, glycogen storage was decreased while ectopic fat deposition was increased (Fig. 6l, m), suggesting reversal to peripheral insulin resistance.

**Fig. 7:**
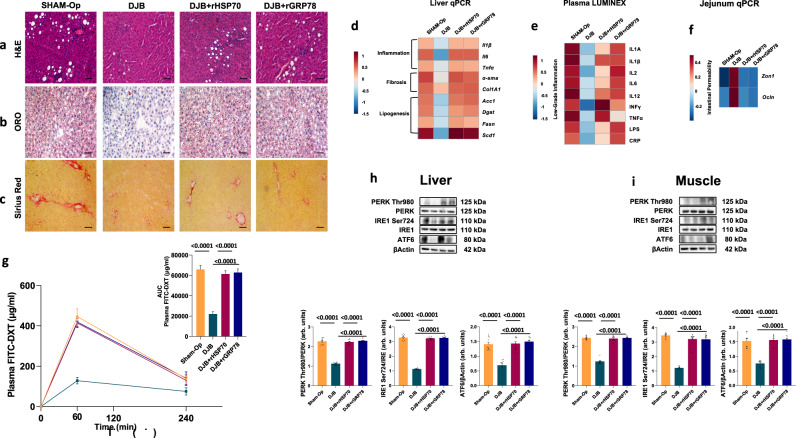
Effects of the infusion of HSP70 and GRP78 in Duodenal-Jejunal Bypass (DJB) on NASH hallmarks. **a**–**c** Representative Hematoxylin and Eosin (H&E) (**a**), Oil Red O (ORO) (**b**), and Picro Sirius Red (**c**) staining of liver sections from rats fed HFD that underwent Sham-operation (Sham-op) or DJB and infused with recombinant HSP70 or GRP78 or saline solution. **d** Gene expression of liver inflammation and fibrosis markers as well as key enzymes involved in hepatic de novo lipogenesis. **e** Plasma low-grade inflammation markers assessed by Luminex. **f** Gene expression of key genes involved in intestinal permeability. **g** Time course and AUC of in vivo intestinal permeability assessed by measuring Fluorescein IsoThioCyanate Dextran 4000 Da (FITC-DXT) in the systemic circulation after an oral gavage of FITC-DXT (500 mg/kg body weight). **h**, **i** Hepatic (**h**) and skeletal muscle (**i**) expression of key proteins involved in Unfolded Protein Response (UPR). Magnification ×20. Scale bar: 0.10 mm. Data are presented as mean value ± SEM of *n* = 10 biologically independent animals. Statistical significances were calculated by one-way Anova with Bonferroni’s correction for multiple comparisons. Source data are provided as a Source Data file.

Infusion of HSP70 or GRP78 abolished the beneficial effects of DJB also on NASH. Hepatic steatosis, inflammation, and fibrosis returned (Fig. 7a–c), and we observed elevated expression of hepatic genes involved in inflammation, fibrosis, and lipogenesis (Fig. 7d). This was similar to sham-operated rats receiving saline infusions. Moreover, infusion of HSP70 or GRP78 contributed to increased levels of pro-inflammatory cytokines, LPS, and CRP in the circulatory stream (Fig. 7e), and, consistent with these results, we observed decreased gene expression of zonulin and occludin in the gut mucosa and a higher plasma FITC-DXT (Fig. 7f, g). We also observed increased phosphorylation of IRE1 Ser724 and PERK Thr980 and expression of ATF6 in the liver and the skeletal muscle when HSP70 or GRP78 were continuously infused after DJB (Fig. 7h, i).

Together, these results confirm our hypothesis that infusion of HSP70 and GRP78 can counteract the beneficial effects of DJB on insulin sensitivity and NASH.

#### Impact on the gut microbiota

Gut microbiota alterations in obesity, T2D, and NAFLD have been linked to intestinal, liver, and systemic inflammation^40,41^. Therefore, we sought to investigate how dietary or surgical manipulations of the upper gut could impact on gut microbiota composition and how HSP70 and GRP78 and their blocking antibodies affected gut microbiota composition.

To determine whether the infusion of recombinant proteins or blocking antibodies affected gut microbiota, we profiled the V4 region of the 16 S rRNA gene in small intestinal (i.e., duodenum, jejunum, and ileum) and fecal samples from a subset of animals (see Methods). Samples clustered depending on the tissue (adonis *p* = 0.001, r^2^ = 0.144, Supplementary Fig. S11A), with large variability in both sequencing depth and microbiota profiles for small intestinal samples (Supplementary Fig. S11A; sufficient data could not be obtained for 20 small intestinal samples as described in Methods). Therefore, the effects of recombinant proteins and blocking antibodies infusions were investigated in fecal samples. Overall fecal microbiota composition was strongly affected by the diet (adonis *p* = 0.001, r^2^ = 0.096, Supplementary Fig. S11B) and by the infusion of both recombinant proteins (adonis; CD-fed: *p* = 0.009, r^2^ = 0.412; DJB-treated: *p* = 0.001, r^2^ = 0.259) and blocking antibodies (HFD-fed: *p* = 0.004, r^2^ = 0.348) (Supplementary Fig. S11C).

Analysis of abundance in fecal samples showed that different taxa were affected by diet (CD and HFD) and by DJB surgery (Supplementary Fig. S12, Source Data). We observed consistent changes for the abundance of taxa in *Bifidobacterium* and *Christensenella*, and in the Erysipelotrichaceae *Dubosiella*, *Allobaculum*, and *Faecalibaculum*, which decreased with the infusion of recombinant proteins and increased with the infusion of blocking antibodies (Source Data). Therefore, our results indicate that systemic modulation of HSP70 and GRP78 influences overall gut microbiota composition and abundance of taxa associated to metabolic health^42^.

To test the link between metabolic/inflammatory phenotypes and gut microbiota, we performed distance-based redundancy analysis (db-RDA) and observed that the variation in gut microbiota composition correlated significantly with glucose tolerance (glucose 120 min, *p* = 0.001), liver function (AST, *p* = 0.005), and systemic inflammation (i.e., LPS, *p* = 0.046), as well as with development of adiposity through the diet (group and SAT, *p* = 0.001) (Supplementary Fig. S13A). In addition, the variation of gut microbiota composition correlated significantly with the fecal proportions of taxa ASV_2280 (*Ruminococcus*) (*p* = 0.026) and ASV_0920 (*Allobaculum*) (*p* = 0.001) in analyses performed on all fecal samples (Supplementary Fig. S13A), and, also, ASV_0918 (*Dubosiella*) (*p* = 0.001) in analyses done on CD and HFD samples (Supplementary Fig. S13B). As previous studies have suggested a possible role of these taxa in modulation of intestinal mucus metabolism, immune function, and metabolic status^42,43^, we performed Spearman’s rho correlation analysis with metabolic/inflammatory variables, and observed that the fecal proportions of ASV_0918 and ASV_0920 correlated positively with the expression of zonulin and occludin, and negatively with jejunal and systemic levels of GRP78, systemic inflammation (e.g., CRP and LPS), liver damage (i.e., ALT and AST), adiposity, blood lipids (i.e., TG), and insulin resistance (e.g., HOMA-IR). Opposite correlations were observed for ASV_2280, which correlated positively with adiposity, glucose intolerance, AST, and circulating as well as intestinal levels of HSPs (Supplementary Fig. S13C). Human studies in Europe and in the US have found that the abundance of gut microbes from *Ruminococcus* and *Erysipelotrichaceae* is significantly altered in the fecal microbiota of individuals with NAFLD^44–48^. Therefore, our results have translational value for a better understanding of the gut microbiota role in NAFLD, as human taxa in *Ruminococcus* have been positively associated with fibrosis independent of metabolic factors^46^ and have been found to be discriminant for cirrhosis^44^, while characterized (e.g., *Holdemania* and *Catenibacterium*) as well as uncharacterized *Erysipelotrichaceae* have been negatively associated with steatosis^47^, fibrosis^45,46^, and cirrhosis^48^.

We then aimed to evaluate if recombinant HSPs repressed and their mAbs had an effect on the bile acid and/or fibroblast growth factor-15 (FGF15) pathway. In fact, bile acids activate the intestinal receptor, Farnesoid-X, that stimulates the synthesis of FGF15, a gut hormone repressing hepatic lipogenesis, but whose action is defective in obese mice^49^. We found a significant increase of circulating total bile acids compared with isotype control infusion (Supplementary Figs. S6, S9, S10). To assess whether the total bile acids levels influenced gut microbiota composition, we added the total bile acids in the distance-based redundancy analysis (db-RDA). We found no significant correlation between the circulating levels of total bile acids and the variation in gut microbiota composition (*p* = 0.137). Moreover, we did not find any significant change in FGF15 plasma concentrations (Supplementary Fig. S14A–C).

### Human studies

#### Fasting serum levels of HSP70 and GRP78 in subjects with or without NASH

To investigate the translational implications of our research models, we investigated whether fasting serum levels of HSP70 and GRP78 were higher in subjects with NASH than in healthy controls. To this end, 47 subjects with histological-proven NASH (NAS ≥ 3) (BRAVES trial, ClinicalTrials.gov Identifier: NCT03524365) and 39 otherwise healthy control subjects (LIBRA trial, ClinicalTrials.gov Identifier: NCT04677101) biopsied during an elective cholecystectomy were included in the study. The anthropometric and laboratory characteristics of the participants are reported in the Supplementary Table S3.

**Fig. 8:**
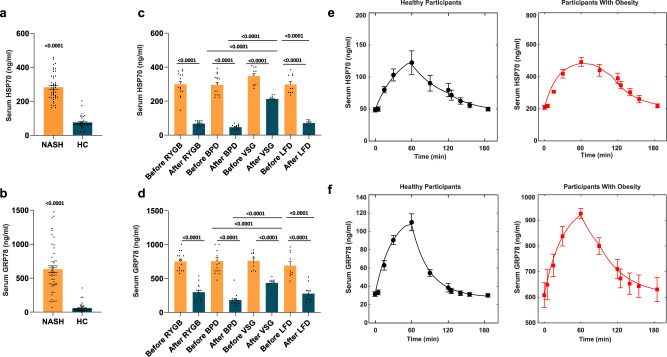
Human studies. **a**, **b** Fasting serum levels of HSP70 (**a**) and of GRP78 (**b**) in people with Non-Alcoholic Steato-Hepatitis (NASH) (*n* = 47 biologically independent samples) and in healthy participants (*n* = 39 biologically independent samples). **c**, **d** Serum levels of HSP70 (**c**) and of GRP78 (**d**) before and after Roux-en-Y Gastric Bypass (RYGB), Biliopancreatic diversion (BPD) (*n* = 15 biologically independent samples), Vertical Sleeve Gastrectomy (VSG) and Low-Fat Diet (LFD) (*n* = 10 biologically independent samples). **e**, **f** Serum HSP70 (**e**) and GRP78 (**f**) time course after a high-fat meal test in healthy participants and participants with obesity (*n* = 5 biologically independent samples). Data are presented as mean value ± SEM. Statistical significances were calculated by unpaired two-tailed *t*-test and one-way Anova with Bonferroni’s correction for multiple comparisons, where appropriate. Source data are provided as a Source Data file.

Indeed, we found that HSP70 concentration was three times larger, and that GRP78 concentration was more than 10 times larger, in subjects with NASH than in healthy controls (Fig. 8a, b).

We obtained a good separation of subjects with histological-proven NASH versus subjects with normal liver by using a two-step partitioning cluster analysis with HSP70 and GRP78 as continuous variables and histological liver scores, i.e., SAF-S, SAF-A, and SAF-F, as categorical variables. The cluster quality, cluster comparison, centroid values, and predictor importance are reported in Supplementary Fig. S15A–D.

Using the NAFLD Activity Score (NAS), a histological scoring system for NAFLD, as dependent variable and GRP78 and HSP70 as independent variables in a linear regression analysis, we obtained a good fit with R^2^ = 0.78 (*P* < 0.001) and the following equation:

NAS = − 0.368 + 0.009·HSP70 + 0.002· GRP78, with a significance of 0.050 for the constant and <0.001 for both HSP70 and GRP78.

The above equation shows that each increase of 100 pg/ml in HSP70 circulating levels translates into a 0.9-point increase in NAS and that at each 100 pg/ml increase in serum GRP78 levels corresponds to a 0.2 increase in NAS. Therefore, if both proteins’ levels increased by 100 units, NAS would increase by 1.1 points.

The standardized coefficients were 0.581 for HSP70 and 0.438 for GRP78, meaning that both HSPs contributed in a similar manner to the increase of the liver histological score and thus of NASH severity.

#### Fasting serum levels of HSP70 and GRP78 in subjects before and after metabolic surgery or low-fat diet

Patients with class III obesity (BMI ≥ 40.0 kg/m^2^) who were scheduled to undergo RYGB (*n* = 12, 11 men) or BPD (*n* = 12, 6 men) surgeries at Catholic University in Rome, Italy, participated in this study (ClinicalTrials.gov number: NCT03111953). The average weight loss, obtained after 20% weight reduction, was 30.5 ± 5 kg for RYGB and 33 ± 6.1 kg for BPD.

To confirm that the jejunum was the major site of gut mucosal secretion, we also included two treatments that do not involve intestinal tract modifications. To this end, we studied 10 people (6 men) who underwent vertical sleeve gastrectomy (VSG) with a mean weight reduction of 42.89 ± 4.32 kg and 10 people (6 men) who underwent a low-fat diet (LFD) with a weight loss of 15.41 ± 2.74 kg at 1-year follow-up (BRAVES trial, ClinicalTrials.gov Identifier: NCT03524365).

The results of insulin sensitivity and glucose kinetics modifications have been previously published^17^. We found that, compared with matched-percentage weight loss induced by RYGB, BPD determined a significantly greater improvement in insulin sensitivity^17^.

Fasting HSP70 and GRP78 decreased after all surgical procedures (Fig. 8c, d) but the effect of VSG in reducing the circulating levels of both HSP70 and GRP78 was significantly lower than that of both RYGB and BPD in spite of the larger weight loss achieved after VSG. Moreover, LFD elicited a greater decrease of serum HSP70 and GRP78 than VSG (see Supplementary Table S4).

Although the large interindividual variability and the small number of subjects precluded finding a significant difference between BPD and RYGB, the reduction in the circulating levels of the two proteins tended to be larger after bypassing both duodenum and jejunum with BPD than after bypassing the duodenum alone with RYGB. These results point towards a dominant role of the upper gut in HSPs secretion under fat meal stimulation but evidence also a possible contribution of fat mass to their circulating levels.

#### HSP70 and GRP78 kinetics after a mixed meal

Next, we investigated the kinetics of HSP70 and GRP78 circulating levels after a 413 kcal high-fat meal (42% carbohydrates, 53% fat, and 5% proteins) in five subjects with obesity (BRAVES trial, ClinicalTrials.gov Identifier: NCT03524365) and five subjects with normal weight (LIBRA trial, ClinicalTrials.gov Identifier: NCT04677101).

Similarly to what was observed in animals, the serum clearance of both HSP70 and GRP78 was well predicted by a nonlinear Michaelis-Menten kinetics, suggesting a receptor-mediated clearance pathway (Fig. 8e, f). The half-life of HSP70 and GRP78 was ca. 40 min in healthy weight subjects and 20 min in subjects with obesity. The kinetic parameters, which had values similar to those found in rodents, are reported in the Supplementary Table S5.

## Discussion

Understanding the molecular mechanisms of diabetes reversal after metabolic surgery has important implications for the development of therapeutic agents to reverse and prevent insulin resistance and low-grade inflammation, which are the major drivers of T2D and NAFLD. We identified two heat shock proteins, secreted by the upper gut, as negative regulators of insulin signaling pathway that are also able to modify gut microbiota and trigger inflammation. This finding partly provides a mechanistic explanation for how the bypass of the duodenum and jejunum in metabolic surgery can reverse insulin resistance, T2D, and NAFLD. Thus, gut-secreted HSP70 and GRP78 are molecular targets for the treatment of T2D and NAFLD, whose inhibition shows a similarly high efficacy to that of metabolic surgery.

HSP70 family is a group of stress response proteins, mainly represented by HSP70-1 and GRP78. Until recently, HSPs have been regarded exclusively as molecular chaperones playing a pivotal role in protein folding. However, literature data show detrimental effects of HSP70 overexpression and beneficial effects of reduced expression of GRP78.

Hypothesizing that HSP70 could have been a major candidate in longevity, Vanhooren et al.^50^. created HSP70 transgenic mice that instead had a shorter lifespan dying before 18 months of life since they developed a series of tumors.

When placed on HFD, GRP78 partial knockout (*Grp78*^+/−^) mice are more resistant to the development of hyperglycemia and hyperinsulinemia, as well as liver steatosis and inflammation of the white adipose tissue^51^.

While HFD promotes HSP70 and GRP78 synthesis in the upper gut of rats and their secretion, duodenal and jejunal bypass prevent the metabolic effects of HFD. Similar results are reproduced in humans, where the longer the bypass of the small intestine the greater is the reduction of circulating levels of HSP70 and GRP78 and the better the metabolic improvements. Accordingly, the metabolic effects of VSG in subjects with obesity are significantly smaller than those of RYGB and BPD but also smaller than those of a low-fat diet showing the importance of the upper gut bypass as well as the relevance of reducing fat intake in the amelioration of the metabolic derangement associated with insulin resistance.

In a genetic model of obesity and diabetes, the male Zucker (*fa/fa*) rats prone to develop T2D caused by an inherited insulin-resistant gene, an increased oxidative stress^52^ can promote secretion of HSP70 and GRP78.

Prolonged HFD promotes ER stress in the colon^53,54^; however, literature is lacking regarding the effects of HFD on upper gut ER stress.

Unfolded and misfolded proteins tend to form insoluble aggregates that are cytotoxic. As a mechanism of defence, cellular organelles, including ER and mitochondria, form a multi-layer proteostasis network (PN)^55^ ensuring that misfolded proteins are removed through the ubiquitin–proteasome system or the autophagosomal-lysosomal pathway^56,57^. ER stress activates UPR to restore ER proteostasis.

UPR is controlled by three proteins, IRE1, ATF6, and PERK, possessing both ER luminal and cytosolic domains^58^. In the absence of significant protein folding abnormalities, IRE1, PERK, and ATF6 remain in an inactive status by associating with GRP78^58^. The presence of misfolded or unfolded protein species causes the dissociation of IRE1, PERK, and ATF6 from GRP78, their enhanced kinase and RNase activity and consequent promotion of protein degradation^59^. The p50-ATF6 fragment cleaved from ATF6 upregulates the expression of GRP78^59^.

Under stress conditions, GRP78 is thus overexpressed and a portion of it can escape the C-terminal KDEL motif retention signal, which permits its retention inside the ER, and migrate on the cell membrane from where it is secreted^60^. A similar mechanism can be hypothesized for HSP70 secretion.

In our series, HFD as well as HSP70 or GRP78 infusion resulted in ER stress of the duodenal and jejunal epithelium, with upregulation of HSP70 and GRP78 gene and protein expression and a profound induction of IRE1, PERK, and ATF6 cellular levels. Antibody infusion did not reverse gut epithelial UPR triggered by HFD but reduced circulating levels of HSP70 or GRP78 by binding to them both in the upper gut epithelium and in the circulatory stream. In fact, we showed that the small intestine is the mAbs’ major clearing organ, beyond the liver and kidney. Binding to circulating HSPs reduces their levels in the blood stream, as it occurs, for instance, with specific mAbs directed against tumor necrosis factor (TNF)-alpha^61^.

Once secreted by the upper gut into the circulatory stream, GRP78 or HSP70 reach the insulin target tissues where they bind to the cell surface TLR4 inducing ER stress. We cannot however exclude the binding of these HSPs to other cellular receptors.

Interestingly, Cani et al.^33^. showed that continuous subcutaneous infusion of the TLR4 agonist LPS in mice for four weeks increased body weight to the same extent as HFD, with a similar distribution between visceral and subcutaneous adipose depots but did not trigger NAFLD. Another important difference with our findings is that while HSPs infusion induce both hepatic and whole-body insulin resistance, liver but not whole-body insulin resistance was detected in the study of Cani et al.^33^. Moreover, infusion of HSP70 or GRP78 increase systemic inflammatory response markers, as well as LPS from the Gram-negative intestinal microbiota due to enhanced gut mucosa permeability, thus triggering the low-grade chronic systemic inflammation typical of obesity and insulin-resistance status, while mAbs infusion and metabolic surgery do the inverse.

We found that mAbs infusion increased the circulating levels of total bile acids that are implicated in insulin signaling^62^. However, taurocholate, at concentrations lower than plasma levels in rat on HFD to exclude acute cytotoxicity and extraction of molecular cholesterol from cell membranes^38^, partially rescued the insulin-resistance effects of HSP70 or GRP78 in hepatocytes and myocytes, suggesting other possible mechanisms, such as reduced ER stress.

An open question is whether it is the increased intestinal permeability caused by HSP70 or GRP78 infusion that elicits NASH onset through gut microbiota changes and bacteria translocation or rather if NASH is triggered directly by the above HSPs.

There is a long-standing debate about whether the alteration of gut permeability is a cause or a consequence of NAFLD.

Chiu et al.^63^ showed that colonization of humanized gnotobiotic mice with fecal bacteria from individuals with NASH exacerbated liver steatosis and inflammation caused by HFD, but it was ineffective if mice were receiving a normal diet. Le Roy et al.^64^. inoculated two groups of germ-free mice with the microbiota of mice that were sensible or resistant to HFD in terms of metabolic derangement. Hyperglycemia and hyperinsulinemia developed in germ-free mice in association with distinct microbiota patterns but the mice were maintained on HFD.

Here we show that HSP70 and GRP78 infusion in rats on CD caused increased gut permeability and alterations of the gut microbiota composition, which, however, was different from that of their mates on HFD. Similarly, infusion of mAbs against HSP70 or GRP78 in rats on HFD reduced gut permeability and modified gut microbiota composition, which however did not become similar to that of rodents on chow diet. Therefore, we can argue that HSP70 and GRP78 directly increase gut permeability and modify gut microbiota composition.

Indeed, heat stress damages the intestinal mucosa and increases intestinal permeability^65^.

A limitation of our study is the lack of a jejunal conditional gene knockout model showing a reduced effect of HFD in inducing insulin resistance and NAFLD, which may be pursued as part of further research. We did not use metabolic cage systems to measure O_2_ consumption and CO_2_ production as well as energy expenditure, respiratory quotient and physical activity that might have shed more light on the key metabolic differences following exposure of recombinant proteins or respective blocking antibodies. Moreover, we included only male rats in our study as do the majority of investigators because they show metabolic disease better than females^66^. However, in a future study we will investigate the sex differences in metabolic homeostasis and disease and the role of gut HSPs.

Future research should include also a better characterization of the mechanisms of secretion and uptake of extracellular HSP70 and GRP78, identification of receptor antagonists of these two proteins, and the study of the effect of their subcutaneous infusion in germ-free mice.

Our study shows that the upper gut secretes heat shock proteins in response to high-fat diet causing insulin resistance, hyperglycemia, and NASH. In fact, continuous infusion of HSP70 or GRP78 mimics the metabolic effects of HFD, while blocking mAbs reverses the metabolic derangement consequent to HFD with an effect similar to metabolic surgery. The metabolic benefits of metabolic surgery are reversed by infusion of HSP70 or GRP78 recombinant proteins.

The data from this study underlying the small intestine’s role in metabolic diseases point towards its therapeutic potential to reverse type 2 diabetes and NASH.

## Methods

### Rodent studies

#### Study design

All animal procedures were approved by the Catholic University of Rome Institutional Animal Care Committee. One-hundred eight adult male Wistar rats, aged 8–10 weeks, and ten male Obese fa/fa Zucker Diabetic Fatty rats (ZDF), aged 6–8 weeks were included in the study.

The rats were housed in individual cages at 22 °C with 12 h light cycles and had ad libitum access to food and water. Rats were either fed a standard chow diet (67% Carbohydrate, 20% Protein, 13% fat) or a high-fat diet (20% Carbohydrate, 20% Protein, 60% fat).

Body weight and food intake were monitored weekly. After 1 week of acclimation, rats were randomly assigned to one of the following groups:

A: Chow diet (CD) for 16 weeks (*n* = 4 Wistar rats)

B: High-fat diet (HFD) for 16 weeks (*n* = 4 Wistar rats)

C: Sixteen weeks of CD (*n* = 10 ZDF rats)

D: Sixteen weeks of CD followed by 4 weeks of continuous infusion of recombinant HSP70 (*n* = 10 Wistar rats) or GRP78 (*n* = 10 Wistar rats) or saline solution (*n* = 10 Wistar rats).

E: Sixteen weeks of HFD followed by 4 weeks of continuous infusion of monoclonal antibodies against HSP70 (*n* = 10 Wistar rats) or GRP78 (*n* = 10 Wistar rats) or Ab Isotype (*n* = 10 Wistar rats).

F: Sixteen weeks of HFD followed by DJB (*n* = 30 Wistar rats) or sham operation (*n* = 10 Wistar rats). Twenty rats of the DJB group were continuously infused with recombinant HSP70 (*n* = 10 Wistar rats) or GRP78 (*n* = 10 Wistar rats) while the remaining rats (*n* = 10 Wistar rats) and the sham-operated animals (*n* = 10 Wistar rats) were infused with saline solution and used as controls.

All infusions with recombinant proteins (2.5 ng/h), monoclonal antibodies (1 µg/h), Ab isotype (1 µg/h), or saline solution were performed through osmotic pumps (Model 2004, Alzet Osmotic Pumps, Cupertino, CA). Recombinant proteins were obtained from Mybiosource (San Diego, CA) while monoclonal antibodies and mouse isotype IgG were obtained from Santa Cruz Biotechnology (Dallas, Texas) mouse IG. The design of the study is summarized in Supplementary Fig. S16A–F.

#### Intestinal heat shock protein secretion

Rat intestinal mucosa was scraped off from the duodenal, jejunal and ileal specimens and incubated for 1 h in oxygenated (O2:CO2, 95:5, v/v) Krebs-Henseleit solution (37 °C, pH 7.4) added with complete protease inhibitor cocktail (Roche, Basilea, CH), to isolate proteins secreted into the medium. One aliquot of conditioned medium from the jejunum was then lyophilized and stored at −80 °C waiting to be processed by iTRAQ LC-MS/MS Analysis.

HSP70 and GRP78 concentrations were measured in the conditioned medium from the duodenal, jejunal and ileal specimens using ELISA (see below). Cell death was assessed by propidium iodide (R&D Systems, Inc., Minneapolis, MN) staining (10 μg/mL) using flow cytometry (Supplementary Fig. S17A).

#### iTRAQ LC-MS/MS analysis

Protein concentrations were determined by BCA assay (Pierce, Rockford, IL). iTRAQ labeling was performed on 90 μg of protein using the reagents and protocols supplied in the 8-Plex Multiplex kit (Applied Biosystems, Framingham, MA) to run 8 samples (4 HFD and 4 chow diet proteins from the conditioned medium). Proteins were dissolved in 0.5 M triethyl ammonium bicarbonate (TEAB) (Sigma–Aldrich, St. Louis, MO) at pH 8.5 buffer with 0.1% sodium dodecylsulfate, reduced with tris(2-carboxyethyl) phosphine (TCEP) (Fluka, St. Louis, MO). The reduced proteins were alkylated with 10 mM methyl methanethiosulfonate (MMTS) (Fluka St. Louis, MO) at room temperature for 20 min, and digested overnight with sequencing-grade, modified porcine trypsin (Promega, Madison, WI).

The eight iTRAQ Reagents—8plex were prepared by adding 70 µl of ethanol to each vial. The solution was transferred to each corresponding sample tube and incubated at room temperature for 2 h. The samples were mixed and analyzed.

Detergent and salts were removed first by strong cation exchange cartridge (Applied Biosystems, Framingham, MA) and then a small reversed-phase column (Strata C18 E, Phenomenex, Macclesfield, UK). Labeled peptides were dried by vacuum concentration and suspended in 0.1% trifluoroacetic acid for LC-MS/MS. A 3-μl aliquot was injected directly onto a polystyrene-divinylbenzene (PS DVB) monolithic column (Dionex Corporation, Amsterdam, The Netherlands, 200 μm i.d. × 5 cm) and UltiMate HPLC system (Dionex, Camberley, UK) with an acetonitrile gradient containing 0.1% heptafluorobutyric acid at a flow rate of 3 μl/min. A Probot microfraction collector (Dionex, Camberley, UK) was used to collect 6-s fractions onto a prespotted anchor chip containing 4-hydroxy-α-cyano-cinnamic acid (Bruker Daltonics, Bremen, DE). MALDI-MS was performed with a Bruker ultraFlex III with WARP-LC software (version 1.1) (Bruker Daltonics, Bremen, DE). Positive-ion spectra of each fraction were acquired in reflector mode, using a Nd:YAG smart beam laser, over a mass range of *m/z* 800–4000. Monoisotopic masses were obtained through flexAnalysis (version 3.0) (Bruker Daltonics, Bremen, DE) using a SNAP averagine algorithm (C 4.9384, N 1.3577, O 1.4773, S 0.0417, H 7.7583) from smoothed (Savitsky-Golay, width 0.2 *m/z*, 1 cycle), baseline subtracted (TopHat) spectra with a signal to noise threshold of 3. The 10 most intense ions with a signal-to-noise ratio greater than 35 were selected for LIFT-MS/MS. LIFT spectra were processed using baseline-subtraction (TopHat) and smoothing (Savitsky-Golay, width 0.15 *m/z*, 4 cycles). Peak lists were created through flexAnalysis (version 3.0) from centroided data below m/z 122 and SNAP averaging algorithm (C 4.9384, N 1.3577, O 1.4773, S 0.0417, H 7.7583) above. Peak lists were submitted to a locally-running copy of Mascot (version 2.1) (Matrix Science Ltd., London, UK) via biotools (Bruker Daltonics, Bremen, DE) and searched against the IPI rats database. The significance threshold was set to 0.1 which gave a false discovery rate at the homology level of 0.99%. Peptides were further filtered with a minimum expect score of 0.05. Only proteins with at least two peptides that meet these criteria are reported. To determine which iTRAQ ratios were significantly different from unity, the method described by Pierce et al.^67^. was used to account for experimental variability. Threshold ratios were determined from a log plot of known ratios from internal replicates versus the number of peptides contributing to the ratios; only ratios that exceed these thresholds were considered significant. From such a plot, threshold ratios of 1.3 and 0.8 were chosen.

#### Insulin-mediated glucose uptake in rat primary myocytes

Skeletal muscle was dissected from the hind limb thighs according to a previously published protocol^68^ with some modification. Briefly, tissue samples were enzymatically disassociated using 0.05% of trypsin–EDTA (Merck, Darmstadt, DE) solution for 60 min in a 37 °C water bath with agitation of 100 rpm. After 60 min, the trypsin solution was removed and Dulbecco’s Modified Eagle Medium (DMEM) (Merck, Darmstadt, DE) medium supplemented with 10% fetal bovine serum (FBS) (Merck, Darmstadt, DE) was added to block trypsin action. The tissue was centrifuged at 300 × *g* for 10 min at 4 °C. The pellet was resuspended with DMEM medium supplemented with 10% FBS and plated in uncoated 100 mm Petri dishes for 20–30 min, to allow contaminating fibroblasts to settle out. Then the supernatant was centrifuged at 300 × *g* for 10 min at 4 °C. The pellet was resuspended in DMEM medium supplemented with 10% FBS. Purified myocytes were maintained in a 5% CO2 incubator. Cells were cultured for 24 h before experiments. Prior stimulation, cells were incubated in serum free DMEM overnight. To assess insulin-mediated glucose uptake myocytes were pretreated for 24 h with recombinant proteins (MyBiosource, San Diego, CA) and with insulin (100 nM) (Merck, Darmstadt, DE) for 10 min. Concentration for recombinant proteins used were: Hb 14 ng/ml; GAPDH 70 ng/ml; CMPK1 20 ng/ml; Idh1 200 ng/ml; DAK 10 ng/ml; Gsto1 10 ng/ml; PPIA70 ng/ml; Atp1b1 25 ng/ml; PFN1 30 ng/ml; ALB 40 ng/ml; Adh1 25 ng/ml;GRP78 3 ng/ml; ANXA4 200 ng/ml; HSP70 15 ng/ml; LGALS2 100 ng/ml; ACTB 4 ng/ml; nme2 100 ng/ml; mgam2 100 ng/ml; RBP2 20 ng/ml; Aldob 40 ng/ml; GStP2 10 ng/ml; DBI 30 ng/ml; Ldha 20 ng/ml; Act4 5 ng/ml; RPL14 25 ng/ml; trx1 50 ng/ml; Papss2 10 ng/ml; GPI1 40 ng/ml; tpi1 18 ng/ml; Prss1 200 ng/ml; Mdh1 200 ng/ml; Ephx2 100 ng/ml; Aldh1a1 100 ng/ml; Prdx5 5 ng/ml; Mdh2 20 ng/ml; Ckb 20 ng/ml; Fbap1 150 ng/ml; Gstpm1 20 ng/ml; Fabp2 2 ng/ml; Gsta2 20 ng/ml; krt19 33 ng/ml; Ces2 100 ng/ml; Eno1 20 ng/ml; pgk1 2 ng/ml; Gstm3 20 ng/ml; PRDX1 15 ng/ml; Keratin 8 1 ng/ml. Glucose uptake was evaluated using Glucose Uptake Assay Kit (Abcam, Cambridge, UK) following manufacturer’ instructions.

#### Intestinal epithelial and enteroendocrine cells isolation

Rat jejunal mucosa was prepared by removing the longitudinal muscle layer and serosa and washing with ice-cold Hanks’ Balanced Salt Solution (HBSS). The supernatant was removed and the settled contents were washed four times. Then, the settled contents were removed, minced and suspended in HBSS. The suspension was clarified with a 1000 μm^2^ mesh filter and incubated in digestion buffer containing 75 U ml^−1^ collagenase type XI, 20 μg ml^−1^ dispase neutral protease II, 0.5 mM Dithiothreitol (DTT), and 1% v/v FBS in DMEM. The digestion buffer containing the tissue was placed in a 37 °C incubator and shaked at 180 rpm for 3 h. The digestion mixture was filtered again, washed with 25 ml DMEM containing 2% w/v d-sorbitol and centrifuged at 200 × *g*, for 4 min, at 4 °C^69^. The mucosal cells isolated were stained with anti-Integrin β4 (B-7)-FITC and anti-Claudin-4 (A-12)-Alexa Fluor 647, while dead cells were excluded by propidium iodide (PI) staining. The stained cells were analyzed using a flow cytometer FACSAria (BD Biosciences, Franklin Lakes, NJ), and suitable cell populations gated on PI − Claudin-4− Integinβ4+ and PI − Claudin-4+ Integinβ4− were sorted. Epithelial cell fraction was defined by PI − Claudin-4− Integinβ4+ while enteroendocrine cell fraction was defined by PI − Claudin-4+ Integinβ4−. After cell sorting the cells were further analyzed by flow cytometry and immunofluorescence using Claudin-4 and Integinβ4 antibodies to assess cell purity, while the remaining cells were resuspended in S-DMEM, plated in a 24-well culture dish and incubated at 37 °C, 7.5% CO_2_. Cells were allowed to settle for 48 h and were successively stimulated with palmitic acid (0.5 mM) for 24 h. HSP70 and GRP78 concentrations were measured in the cell media using ELISA. Antibody for flow cytometry were diluted 1:50 in phosphate buffered saline (PBS) while, antibody for immunofluorescence were diluted 1:200 in phosphate buffered saline (PBS).

#### Western blot analysis

Liver, skeletal muscle and intestinal (jejunum) specimens were homogenized in RIPA buffer containing a cocktail of protease inhibitors. Homogenates were cleared by centrifugation (19,000 × *g*; 30 min, 4 °C). The protein content was determined using Bradford Protein Assay (Bio-Rad Laboratories, Hercules, CA). Protein lysates (30 μg) were separated on 10% SDS-PAGE, transferred on PVDF membrane and blocked with EveryBlot Blocking Buffer (Bio-Rad Laboratories, Hercules,CA) for 5 min. Membranes were probed overnight with phospho-Akt Ser473, GSK3αβ Ser21/9, FoxO1 Thr24, GLUT4, phospho-PERK Thr980, phospho-IRE1 Ser724, ATF6, or Actin. Membranes probed with phospho-antibodies were stripped for 30 min at 56 °C and re-probed overnight with total Akt, GSK3αβ, FoxO1, PERK, or IRE1. Detection and analysis were performed, respectively, with Chemidoc XRS Image system and Image Lab 5.0 software (Bio-Rad Laboratories, Hercules, CA). Phosphorylated proteins were normalized with the total corresponding protein and expressed as a phospho-protein/total protein ratio while GLUT4 and ATF6 were normalized with Actin^70^. Antibodies against phospho-Akt Ser473, phospho-GSK3αβ Ser21/9, pospho-FoxO1 Thr24, PERK Thr980, total Akt, total GSK3αβ, total PERK, and total IRE1, were obtained from Cell Signaling Technology (Danvers, MA). Total FoxO1, GLUT4, ATF6, and Actin were obtained from Santa Cruz Biotecnology (Dallas, TX). Phospho-IRE1 Ser724 was obtained from Abcam (Cambridge, UK). Primary antibody were diluted 1:1000 in EveryBlot Blocking Buffer, while secondary antibodies were diluted 1:5000 in EveryBlot Blocking Buffer.

#### Native and recombinant heat shock protein kinetics

HSP70 and GRP78 serum levels were measured before and during a 2 h infusion of the recombinant proteins via a subcutaneous osmotic pump and after its removal, while the kinetics of native HSP70 and GRP78 was assessed as after an intragastric load of palm oil.

The mathematical model for the in vivo kinetics of HSP70 and GRP78 has been written as follows:

1 $$\frac{dc}{dt}=A(1-{e}^{-{k}_{1}t})-\frac{1}{V}\frac{{T}_{m}c}{{K}_{m}+c},\ c(0)=0,\ t\pounds {t}_{{{{{{\mathrm{max}}}}}}}$$

2 $$\frac{{dc}}{{dt}}=A{e}^{-{k}_{2}\left(t-{t}_{\max }\right)}-\frac{1}{V}\frac{{T}_{m}c}{{K}_{m}+c},\ t \, > \, {t}_{\max }.$$

In^1^ and^2^, the positive term in the right-hand-side of the equation represents the inflow into the plasma of the recombinant protein delivered subcutaneously (in rats), or the inflow of the native protein released by the gut in response to the meal (in rats and humans). The negative term represents the receptor-mediated protein uptake by tissues as modeled by a Michaelis-Menten function. The increment over basal value of plasma concentration (ng/mL) of the protein (HSP70 or GRP78) is denoted by *c*; *V* (mL) is the plasma distribution volume; *A* (ng/(mL× min)) is the maximal inflow rate of the protein; *k*_*1*_ and *k*_*2*_ are the rates (1/min) of inflow increase and, respectively, decrease); *T*_*m*_ (ng/min) and *K*_*m*_ (ng/mL) are the parameters of the Michaelis-Menten function and *t*_*max*_ is the time of maximal measured concentration; *K*_m_ is the substrate concentration at which $v=\frac{{v{{{{{\mathrm{max}}}}}}}}{2}$, in other words *K*_m_ is the substrate concentration at which half of the enzyme’s active sites are saturated with substrate. *K*_m_ is therefore considered a relative measure of the substrate binding affinity or the stability of the enzyme–substrate complex: lower *K*m values imply higher enzyme affinity for the substrate. Model parameters were estimated by fitting the sum *c* + *c*_*b*_ (where *c*_*b*_ is the constant basal concentration of the protein) to the measured concentrations.

The protein half-life (T_1/2_) was determined by finding the time interval between the time of maximal concentration and the time when the incremental concentration *c* reaches half of the maximal value.

#### Model of mAb and HSP kinetics in rats

The mathematical model for the in vivo kinetics of mAb and HSP in rats has been written by following the approach by Berends et al.^71^.

A one-compartment model with volume *V* (L) is used. As the rats were undergoing a high-fat diet before and during the experiment, the total HSP concentration *P*_*t*_ (pM) in serum is assumed to be constant throughout the experiment (and denoted as $\bar{P}$ in the following), while unbound and bound components may change freely. The mAb is delivered as a rapid subcutaneous injection of mass *D* (pmol) and free mAb is eliminated from serum with rate constant *k*_*e*_ (${{{{{{\rm{h}}}}}}}^{-1}$). The time course of the inflow rate of free mAb in serum is denoted as *I* (*t*) (pmol ${{{{{{\rm{h}}}}}}}^{-1}$). The complex mAb-HSP has a binding rate constant *k*_*on*_ (pM^−1^h^−1^), a dissociation rate constant *k*_*off*_ (h^−1^), and elimination from serum (internalization into tissues) with rate constant *k*_*int*_ (${{{{{{\rm{h}}}}}}}^{-1}$). Free mAb concentration in serum is denoted by *A* (pM), mAb-HSP complex concentration by *B* (pM), and free HSP concentration by *P* (pM).

Assuming for simplicity that the antibody binds only one HSP molecule, the balance equations are as follows:

1 $$\frac{{dA}}{{dt}}=\frac{I(t)}{V}-{k}_{{{{{{{\mathrm{on}}}}}}}}{AP}+{k}_{{{{{{{\mathrm{off}}}}}}}}B-{k}_{e}A,\quad A\left(0\right)=0$$

2 $$\frac{{dB}}{{dt}}={k}_{{{{{{{\mathrm{on}}}}}}}}{AP}-{k}_{{{{{{{\mathrm{off}}}}}}}}B-{k}_{{{{{\mathrm{int}}}}}}B,\quad B\left(0\right)=0$$

Still following^71^ we assume in Eq. (2) a quasi-steady state (QSS) approximation [2], which gives

3 $$0={k}_{{{{{{{\mathrm{on}}}}}}}}{AP}-{k}_{{{{{{{\mathrm{off}}}}}}}}B-{k}_{{{{{\mathrm{int}}}}}}B={k}_{{{{{{{\mathrm{on}}}}}}}}{AP}-(k_{{{{{{{\mathrm{off}}}}}}}}+{k}_{{{{{\mathrm{int}}}}}})B,$$

that is, the rate of binding is balanced by dissociation plus internalization rates on the scale of other processes.

A QSS dissociation constant *K*_*SS*_ (pM) is defined as:

$${K}_{{SS}}=\frac{{k}_{{{{{{{\mathrm{off}}}}}}}}+{k}_{{{{{{\mathrm{int}}}}}}}}{{k}_{{{{{{{\mathrm{on}}}}}}}}}={K}_{D}+\frac{{k}_{{{{{\mathrm{int}}}}}}}{{k}_{{{{{{{\mathrm{on}}}}}}}}},$$

where *K*_*D*_ is the dissociation constant in the quasi-equilibrium (QE) approximation. QSS reduces to QE when the internalization rate of complex is much smaller than dissociation rate. Equation (3) now gives $={AP}/{K}_{{SS}}$, leading to $B=A\left(\bar{P}-B\right)/{K}_{{SS}}$ and to

4 $$B=\frac{\bar{P}A}{{K}_{{SS}}+A}.$$

To improve the parameter identifiability^72^, Eqs. (1)–(2) will be rewritten in terms of the total concentrations of mAb (*A*_*t*_) and HSP (*P*_*t*_). We have:

5 $$\frac{d{A}_{t}}{{dt}}=\frac{I(t)}{V}-{k}_{e}A-\frac{{k}_{{{{{\mathrm{int}}}}}}\bar{P}A}{{K}_{{SS}}+A},\quad {A}_{t}\left(0\right)=0$$

The free mAb concentration *A* is obtained by rewriting Eq. (4) as the second-order equation in *A*

$$\left({A}_{t}-A\right)\left({K}_{{SS}}+A\right)=\bar{P}A$$

with nonnegative solution

6 $$A=\frac{1}{2}\left[\left({A}_{t}-\bar{P}-{K}_{{SS}}\right)+\sqrt{{({A}_{t}-\bar{P}-{K}_{{SS}})}^{2}+4{A}_{t}{K}_{{SS}}}\right]$$

To account in a simple way for mAb transport from subcutaneous tissue to blood, we assumed that half of the dose *D* enters the blood from 0 to 120 min (time of maximal concentration) and the other half from 120 min to the end of the experiment. The time course of inflow rate *I* (*t*), such that the area-under-the-curve of *I* (*t*) equals the dose *D*, was computed by using the ratios of measured relative fluorescence units (RFU) as, for instance in the time interval 0–20 min,

$${{{{{{\mathrm{AUC}}}}}}}(I)_{0-20}/{{{{{{\mathrm{AUC}}}}}}}(I)_{0-120}={{{{{{\mathrm{AUC}}}}}}}({{{{{{\mathrm{RFU}}}}}}})_{0-20}/{{{{{{\mathrm{AUC}}}}}}}({{{{{{\mathrm{RFU}}}}}}})_{0-120}$$

where the right-hand-side is known from the data and the denominator of left-hand-side equals *D*/2.

By solving Eqs. (5–6), the model parameters *V*, ${k}_{e}$, ${k}_{{{{{\mathrm{int}}}}}}$ and ${K}_{{SS}}$ were estimated by fitting the time course of experimental fluorescence data, assumed to be proportional with proportionality constant *K* (p${{{{{{\rm{M}}}}}}}^{-1}$) to mAb total concentrations, and setting $\bar{P}$ to the protein concentration value measured before mAb delivery.

#### Surface plasmon resonance

SPR experiments were conducted on a Bruker SPR-32pro (Bruker Daltonics SPR, Hamburg, Germany) equipped with a HCA (high capacity amine) chip. NHS (50 mM), EDC (400 mM), and ethanolamine (1 M) coupling reagents (Bruker) were used to immobilize commercial anti-HSP70 or GRP78 (5 µg/mL, sodium acetate pH 5, flow rate: 5 µL/min, 360 s) to the sensor surface using a standard amine-coupling procedure^73^. Kinetics for native serum protein HSP70 and GRP78 have been performed. Each serum concentration was injected for 180 s at 35 µL/min with 600 s off rate. Interactions were performed at 25 °C. The raw data has been single subtracted using an in-line reference surface using the Bruker R4 analyzer. A global fit with a 1:1 interaction model using a term for mass transport was applied to both data sets.

#### Co-immunoprecipitation and immunoblot

Serum was incubated with primary antibody and Dynabeads Protein G (Life Technology, Carlsbad,CA) for 2 h at room temperature. Beads were washed twice with Phosphate Buffered Saline 1× (PBS). Bound proteins were eluted in 1× Sodium Dodecyl Sulfate (SDS) protein loading buffer containing 1% beta-mercaptoethanol and boiled at 95 °C for 10 min. For immunoblot, samples were separated on 10% SDS-PAGE and electroblotted onto an Immuno-Blot® PVDF membrane (BioRad Laboratories). Membranes were blocked in 5% milk in Tris-buffered saline with 0.1% Tween (TBST) buffer for 1 h at room temperature and incubated with primary antibodies in 3% milk TBST buffer at 4 °C overnight. After several washes, membranes were incubated with HRP-conjugated IgG secondary antibody for 1 h at room temperature and were developed with Chemidoc XRS (BioRad Laboratories). Primary antibody were diluted 1:1000 in EveryBlot Blocking Buffer, while secondary antibodies were diluted 1:5000 in EveryBlot Blocking Buffer.

#### Antibody validation and cross reactivity

Antibody validation was carried out on serum and recombinant proteins by western blot analysis. For western blot analysis we used the methods reported above in the immunoblot section.

Cross reactivity studies were performed on serum depleted of HSP70 or GRP78. Briefly, after co-immunoprecipitation of HSP70, the depleted serum was analyzed by western blot using the antibody directed against GRP78, while the serum depleted of GRP78 was analyzed by western blot using the antibody directed against HSP70.

#### Ex vivo imaging

Rats were anesthetized and injected subcutaneously with 1 μg/ml of mAbs against HSP70 or GRP78 (Alexa Fluor 488 labeled) or 1 μg/ml of antibody isotype (goat anti-rabbit igg (h + l) highly cross-adsorbed secondary antibody, alexa fluor plus 488). Two hours after the injection, five major organs (heart, kidney, spleen, skeletal muscle, visceral adipose tissue, small intestine, and liver) were harvested and immediately subjected to fluorescence imaging using the IVIS imaging system. Optical images were acquired with IVIS Spectrum (Perkin Elmer, Waltham, MA) in fluorescent modality with excitation filter 480 nm and emission filter 570 nm. Other parameters were exposure time 60 s, binning B = 4, f/stop = 2. Fluorescence determinations were expressed as a pseudocolor on a gray background, with yellow color denoting the highest and dark red the lowest intensity. Data were analyzed using Living Image version 4.7.4 software (https://www.perkinelmer.com). Regions of interest (ROIs) were drawn in the area of interest and quantified in the “Radiant Efficiency [p/s/sr]/[µW/cm^2^]”.

#### Interventions

The rats were anesthetized using 75 mg/kg of ketamine and 10 mg/kg of xylazine intramuscularly. Ten milliliters of sterile 0.9% NaCl were administered subcutaneously before surgery for hydration. Access to the peritoneal cavity was obtained by 3 cm laparotomy^74^.

##### Duodenal-jejunal bypass (DJB)

The stomach and proximal duodenum were exposed and blunt dissection of the stomach just above the pylorus allowed for its isolation and division. The duodenal stump was closed by means of a continuous non-absorbable monofilament 6-0 suture. The Treitz ligament was then identified and the jejunum was run 10 cm cephalad and divided. The jejunal stump was cut obliquely on the anti-mesenteric border to allow for a wider gastric outlet and a beveled gastro-jejunal anastomosis was performed using two semi-continuous non-absorbable monofilament 6-0 sutures. The jejuno jejunostomy was then executed ~10 cm distally from the gastro-jejunostomy. The peritoneal cavity was soaked with warm saline and abdominal closure was achieved by separate closure of peritoneum, fascia and skin^18^.

##### Sham operation

In sham-operated rats, a midline laparotomy was performed, and the stomach was exposed and gently manipulated. A 1 cm gastrotomy was performed and then closed as in the DJB group. The abdominal cavity was kept open for the same amount of time required to perform DJB^18^. Survival rates were 95% after sham operation, and 85% after DJB.

##### Osmotic pumps implant

A mid-scapular incision was performed, then a sterile hemostat forceps was inserted into the incision to create a subcutaneous pocket for pump allocation. The pump (Model 2004, Alzet Osmotic Pumps, Cupertino, CA) was inserted into the pocket, delivery portal first, to minimize interaction between the compound delivered and the healing of the incision. The incision was closed with non-absorbable monofilament 4-0 sutures.

#### Peri- and postoperative care

At the end of the surgical procedures, all rats received sterile intraperitoneal injections of 0.9% NaCl to maintain hydration during healing and 5 mg/kg of ketoprofen as an analgesic. They were placed on a heated mat until recovery and then returned to their home cages. The rats were allowed to drink purified water for 12 h after surgery, and a liquid diet containing 5% glucose and 0.2% KCl was provided for the next 48 h^75^.

#### Oral glucose tolerance test (OGTT)

All animals underwent an OGTT at 4 weeks after the implant of the osmotic pump. After an overnight fasting, the rats received a 50% D-glucose solution (1 g/kg body weight) by oral gavage. Blood samples were taken by tail bleeding and collected in EDTA tubes. All blood samples were immediately centrifuged and plasma divided into appropriate subsamples and stored at −20 °C for further analysis. Blood glucose, plasma insulin and C-peptide were measured at 0, 20, 40, 60, 80, 100, 120, and 180 min. Blood glucose levels were measured by glucometer (Accu-Chek, Roche Diagnostics Division, Grenzacherstrasse, CH). Plasma insulin was measured by ELISA (EMD Millipore Corporation, Billerica, MA), with a sensitivity of 0.1 ng/ml and an intra- and interassay precision of 1.9% and 7.6%, respectively. C-Peptide was measured by ELISA (RayBiotech, Peachtree Corners, GA) with a sensitivity of 772 pg/ml and an intra- and interassay precision <10% and <15%, respectively. C-reactive protein (CRP) was measured by ELISA (Cusabio, Houston, TX) with a sensitivity of 7.81 ng/ml and an intra- and interassay precision <8% and <10%, respectively. Lipopolysaccharide (LPS) was measured by ELISA (Cusabio, Houston, TX) with a sensitivity of 0.039 ng/mL and an intra- and interassay precision <8% and <10%, respectively.

#### Mathematical models

##### Measurement of insulin sensitivity (S_I_)

The glucose minimal model^76^ was used to analyze the OGTT data, as it provides a reliable estimate of S_I_ that correlates with the euglycemic hyperinsulinemic clamp. The minimal model is currently used to assess S_I_ (min^−1^ ⋅ pM^−1^) and glucose effectiveness S_G_ (min^−1^) from the OGTT in humans and has been validated in mice^77,78^.

Minimal model equations were written in the form:

$$\frac{{dG}}{{dt}}=-\!\left({S}_{G}+{S}_{I}\cdot Z\right)G+{S}_{G}{G}_{b}+\frac{{Ra}}{{V}_{G}},\ G\left(0\right)={G}_{b}$$

$$\frac{{dZ}}{{dt}}=p\cdot \left(-Z+I-{I}_{b}\right),\ Z\left(0\right)=0$$

$${{{{{\rm{with}}}}}}\ {Ra}=(a\cdot \Delta G+d(\Delta G)/{dt})/b$$

where G (mmol/l) is the glucose concentration (basal value G_b_, and ΔG = G − G_b_), I (pmol/l) is the insulin concentration (basal value I_b_), Z (pmol/l) is a variable related to insulin action, Ra (mmol/min) is the rate of appearance of oral glucose in plasma, V_G_ (l) the glucose distribution volume, and p a parameter (min^−1^) that represents the rate constant of insulin action on glucose disposal.

Model parameters S_G_, S_I_, p, a(bV_G_)^−1^, and (bV_G_)^−1^ were estimated by fitting glucose concentration data. To this purpose, the experimental points were interpolated every 10 min to obtain a sufficiently robust parameter estimate.

##### Measurement of insulin secretion (ISR_AUC_) and insulin sensitivity to glucose (Φ)

C-peptide kinetics was assessed by using the two-compartment model originally proposed by Eaton et al.^79^.

$$\frac{d{{{{{{{\mathrm{CP}}}}}}}}_{1}(t)}{{dt}}=-\!\left[{k}_{01}+{k}_{21}\right]{{{{{{{\mathrm{CP}}}}}}}}_{1}\left(t\right)+{k}_{12}{{{{{{{\mathrm{CP}}}}}}}}_{2}\left(t\right)+{{{{{{\mathrm{SR}}}}}}}\left(t\right),\ {{{{{{{\mathrm{CP}}}}}}}}_{1}\left(0\right)=0$$

$$\frac{d{{{{{{{\mathrm{CP}}}}}}}}_{2}(t)}{{dt}}={k}_{21}{{{{{{{\mathrm{CP}}}}}}}}_{1}\left(t\right)-{k}_{12}{{{{{{{\mathrm{CP}}}}}}}}_{2}\left(t\right),\ {{{{{{{\mathrm{CP}}}}}}}}_{2}\left(t\right)=0$$

CP_1_ and CP_2_ (nmol/l) are C-peptide concentrations above basal in the accessible and in the peripheral compartment, respectively, kij (min^–1^) are C-peptide kinetic parameters; and SR (pmol l^–1^ min^–1^) is the pancreatic secretion above basal, entering the accessible compartment, normalized by the distribution volume of compartment 1. The C-peptide kinetic parameters kij and distribution volumes V_1_ and V_2_ were computed, for each rat, using the approach developed by Ahrén et al.^80^. in mice. In this paper^80^ they find: V_tot_ = V_1_ + V_2_ = 38% of body weight, V1 = 42% of Vtot, k01 = 8.2% of Vtot/V1, k21 = 7.7% of Vtot/V1, and k12 = 7.7% of Vtot/V2. The ISR = SR × V_1_ was obtained, for each rat, by deconvolution of the C-peptide data. Moreover, in order to obtain the ISR time course from 0 to 180 min, we assumed that the value of C-peptide at 360 min was equal to the basal one. The ISR_AUC_ was computed by the trapezoidal rule.

The value of the insulin sensitivity to glucose, Φ, was obtained using the model independent formula^81^

$$\Phi \ \approx \ {k}_{01}\int _{0}^{\infty }C{P}_{1}\left(t\right){dt}/\int _{0}^{\infty }\left[G\left(t\right)-{G}_{b}\right]{dt}$$

where CP1 is the value of the plasma C-peptide, G(t) is the plasma Glucose and Gb the basal value of Glucose.

#### Intestinal permeability in vivo

All animals underwent the permeability test at 4 weeks after the implant of the osmotic pump. After 6 h fasting, the rats received Fluorescein Isothiocyanate–dextran 4000 Da (FITC-DXT) (Sigma–Aldrich, St. Louis, MO) by oral gavage (500 mg/kg body weight).

After 1 h and 4 h, blood was collected from the tip of the tail vein and centrifuged at 4 °C, 12,000 × *g* for 3 min. Plasma was diluted in an equal volume of PBS (pH 7.4) and analyzed for FITC-DXT concentration with a fluorescence spectrophotometer (Varioskan LUX microplate reader, Thermo Fisher Scientific, Waltham, MA) at an excitation wavelength of 485 nm and emission wavelength of 535 nm. Standard curves were obtained by diluting FITC-DXT in plasma obtained before FITC-DXT gavage.

#### Quantitative real-time PCR analysis

Total RNA from liver and small intestine biopsies was extracted using the RNeasy Plus Mini Kit (Qiagen GmbH, Hilden, DE) according to the indications provided by the company. A small aliquot of total RNA obtained (3 μl) was subjected to qualitative and quantitative control by using the microdrop (Thermo Fischer Scientific, Waltham, MA). The qualitative and quantitative assessment of the individual samples was determined using a dedicated software. The total RNA was reverse transcribed into cDNA by using iScript RT (Bio-Rad Laboratories, Hercules, CA)^82^. SYBR Green gene expression assays were performed according to the manufacturer’s instruction using the iQ™ SYBR® Green Supermix (Bio-Rad Laboratories, Hercules, CA) and the CFX96 Touch Real-Time PCR Detection System (Bio-Rad Laboratories, Hercules, CA).

Primer sequences are reported in Supplementary Table S6.

mRNA expression levels were normalized to β2-microglobulin and quantification of relative gene expression, presented as percentage of the relevant baseline, was calculated using the 2-∆CT (comparative threshold) method.

#### Histology

The day of the sacrifice fresh portions of liver and skeletal muscle were cut, embedded in cryo-embedding media (OCT) and snap frozen in liquid nitrogen. Biopsies were cut using a cryostat (5 μm) and slides stored a −20 °C until analyses. Hematoxylin and Eosin staining was performed to assess hepatic steatosis. Slides were fixed 10 min with 95% ethanol, stained with hematoxylin for 1 min, washed with distilled water, stained with eosin for 30 seconds and cleared in two changes of pure ethanol and two changes of xylene. Oil Red O was performed in both liver and skeletal muscle to assess intracellular lipid accumulation. Slides were fixed overnight with 4% formalin, stained with Oil Red O solution for 1 h. Counterstain was performed with Hematoxylin solution.

Periodic acid Schiff staining was used in both liver and skeletal muscle to evaluate glycogen storage. Slides were fixed 20 min with 4% formalin, stained with Periodic Acid Solution for 5 min and with Schiff’s Reagent for 15 min. Counterstain was performed with Hematoxylin solution. Sirius Red was used to identify hepatic fibrosis. Slides were fixed 10 min with 4% formalin, stained in Direct red 80 for 1 h, washed in acidified water and dehydrate in 3 changes of absolute ethanol. After brief clearing in xylene, the slides were mounted in a resinous medium. Images were taken with an optical microscope (Nikon Eclipse e200, Tokyo, J). All reagents for histological analysis were obtained from Sigma–Aldrich (St. Louis, MO)^83^.

### ELISA

Alanine aminotransferase (ALT) and Aspartate aminotransferase (AST) were measured by ELISA (Cusabio, Houston, TX) with a sensitivity of 0.78 mIU/ml and an intra- and interassay precision <8% and <10%, respectively. Triglycerides were measured by a mammalian triglycerides assay (Abcam, Cambridge, UK) with a sensitivity of 2 µM. Total Bile Acids were measured by ELISA (MyBiosource, San Diego, CA) with a sensitivity of 1 µM. FGF15 levels were measured by ELISA (MyBiosource, San Diego, CA) with a sensitivity <5.2 pg/ml and an intra- and interassay precision <10% and <12%, respectively. HSP70 was measured by ELISA (MyBiosource, San Diego, CA) with a sensitivity <1.18 ng/ml and an intra- and interassay precision <10% and <12%, respectively. GRP78 was measured by ELISA (MyBiosource, San Diego, CA) with a sensitivity of 0.043 ng/ml and an intra- and interassay precision <8% and <10%, respectively.

### Luminex

Interleukin 1 α (IL-1α), Interleukin 1β (IL-1β), Interleukin 2 (IL-2), Interleukin 6 (IL-6), Interleukin 12(IL-12), Interferon γ (IFN-γ), and Tumor necrosis Factor α (TNF-α) were measured by a rat cytokine magnetic bead panel (Bio-Rad Laboratories, Hercules, CA) and measured by a Luminex™ 200™ Instrument System and Bio-Plex Manager Software (Bio-Rad Laboratories, Hercules, CA) according to manufacturer’s instruction.

#### DNA extraction and gut microbiota profiling

Total genomic DNA was isolated from 150-200 mg of small intestinal tissue (duodenum, jejunum and ileum from 28 rats: CD, *n* = 3; CD+rHSP70, *n* = 4; CD+rGRP78, *n* = 3; HFD, *n* = 8; HFD+AbHSP70, *n* = 2; HFD+AbGRP78, *n* = 2; DJB+rHSP70, *n* = 3; DJB+rGRP78, *n* = 3) and one fecal pellet (48 rats: CD, *n* = 3; CD+rHSP70, *n* = 4; CD+rGRP78, *n* = 4; HFD, *n* = 8; HFD+AbHSP70, *n* = 4; HFD+AbGRP78, *n* = 5; Sham, *n* = 7; DJB, *n* = 5; DJB+rHSP70, *n* = 5; DJB+rGRP78, *n* = 3) using a modification of the IHMS DNA extraction protocol Q^84^. Samples were placed in Lysing Matrix E tubes (MP Biomedicals) containing ASL buffer (Qiagen GmbH, Hilden, DE), vortexed for 2 min and lysed by two cycles of heating at 90 °C for 10 min followed by two bursts of bead beating at 5.5 m/s for 60 s in a FastPrep®−24 Instrument (MP Biomedicals). After each bead-beating burst, samples were placed on ice for 5 min. Supernatants were collected after each cycle by centrifugation at 4 °C; the DNA was recovered by isopropanol precipitation and purified using the QIAamp DNA Mini kit (Qiagen GmbH, Hilden, DE).

Microbiota composition was analyzed as described previously^85^ by Illumina sequencing (V2 kit, 2 × 250 bp paired-end reads, MiSeq llumina RTA v1.17.28; MCS v2.5) of the V4 region in the 16 S rRNA gene, amplified in duplicate reactions with 515 F and 806 R primers^86^ in the presence of 20 ng (feces) or 100 ng (intestinal tissues) of total genomic DNA. Illumina reads were merged using Usearch version 11 64-bit^87^, allowing for up to 30 mismatches in the alignment of the paired-end reads^88^, whereas discarding reads with a merged length greater than 270 bp and less than 230 bp. The merged reads were quality filtered based on expected errors, removing reads above the threshold of 1. The merged reads were turned into zero-radius operational taxonomic units (Zotus; also known as amplicon sequence variants, ASVs)^89^ by compiling the sequences into sets of unique reads and performing error-correction using the UNOISE3 algorithm^90^, discarding sequences with fewer than 4 reads. The Zotus were assigned taxonomy using DADA2 v.1.10^91^ (assignTaxonomy, minBoot = 50) and assignSpecies, using a formatted version of the Silva version 138 database.

The sequencing depth of small intestinal samples varied extensively (range = 569–140,794), and 8, 11, and 1 samples from duodenum, jejunum, and ileum, respectively, yielded <5000 reads. Therefore, due to low sequencing depth and low number of samples for the small intestinal tissues, microbiota analyses on the effects of recombinant proteins and blocking antibodies infusions were performed only on fecal samples (sequencing depth: median = 65,358; range = 20,060–94,709). For β-diversity analyses, 20,060 sequences were randomly subsampled from each sample to correct for differences in sequencing depth and used to calculate the Bray-Curtis dissimilarity index. Statistical differences in overall gut microbiota composition based on the Bray-Curtis dissimilarity were tested using adonis with 999 permutations. The Wald test implemented in DESeq2 was used in analyses of differential abundance on not subsampled count data; raw *p*-values were adjusted by the Benjamini–Hochberg method^92^ with a false discovery rate of 5%. Prevalence filtering of the ASVs (present in at least 10% samples) was applied when checking for differential abundance between groups. Distance-based redundancy analysis was performed on Bray-Curtis dissimilarity distances for all fecal samples and significant taxa between diets using the vegan package in R. Spearman’s rank–order correlation was used to test the correlation between metadata and differential abundant ASVs. ASVs with a Spearman rho coefficient at a cutoff of 0.3, FDR < 0.05, were plotted in heatmaps constructed based on hierarchical clustering of the differentially abundant ASVs. Statistical analyses and visualizations were performed using the R environment (version 4.0.3).

### Human study

#### Cohorts

In the first study in humans “Fasting serum levels of HSP70 and GRP78 in subjects with or without NASH” we evaluated 47 subjects with histological-proven non-alcoholic steatohepatitis (NASH) (NAS ≥ 3) (Cohort 1: BRAVES trial, ClinicalTrials.gov Identifier: NCT03524365) and 39 otherwise healthy control subjects (Cohort 2: LIBRA trial, ClinicalTrials.gov Identifier: NCT04677101) biopsied during an elective cholecystectomy. Baseline data as well data at 1-year follow-up are included for Cohort 1, the data and safety monitoring board has approved the release and publication of these data at this time.

The primary outcomes of cohort 2 trial have been previously published^93^.

In cohort 1 and 2 trials “HSP70 and GRP78 kinetics after a mixed meal” we investigated the kinetics of circulating HSPs following a high-fat meal.

Five participants affected by obesity and five healthy controls received a 413 kcal high-fat meal (42% carbohydrates, 53% fat, and 5% proteins) (BRAVES trial, ClinicalTrials.gov Identifier: NCT03524365; LIBRA trial, ClinicalTrials.gov Identifier: NCT04677101).

People who underwent vertical sleeve gastrectomy or low-fat diet belonged to the BRAVES study (Cohort 1) and were evaluated at baseline and at 1-year after the intervention (see Supplementary Table S4).

For subjects with NASH, the enrollment exclusion criteria were^1^: regular and/or excessive alcohol uptake (>20 g alcohol/day for women and >30 g alcohol/day for men)^2^; clinical evidence of NAFLD secondary to iatrogenic gastrointestinal or immunodeficiency (HIV infection) diseases^3^; clinical evidence of non-NAFLD hepatic diseases, including hepatitis B or C, or hemochromatosis^4^, Wilson disease^5^, glycogenosis^6^, Alpha-1 antitrypsin deficiency^7^, autoimmune hepatitis^8^, cholestasis liver disease^9^, presence of relevant cardiovascular, gastrointestinal or respiratory diseases, or any hormonal disorder^10^, clinical evidence of decompensated liver disease (Child-Pugh score>7 points)^11^, undergoing narcotics abuse^12^, relevant systemic diseases, and^13^ pregnancy.

Finally, patients with class III obesity (BMI ≥ 40.0 kg/m^2^) who were scheduled to undergo RYGB (*n* = 12, 11 men) or BPD (*n* = 12, 6 men) surgeries at Catholic University in Rome, Italy, participated in cohort 3 study (ClinicalTrials.gov number: NCT03111953). Cohort 3 include data at baseline and at −20% weight loss. The primary outcomes of this trial have been previously published^17^.

The study protocols were approved by the ethical committee of the Università Cattolica S. Cuore in Rome, Italy. All participants provided written informed consent at the time of enrollment. The authors had full access to the data and take responsibility for completeness and accuracy of the data and integrity of their analysis.

#### Anthropometric measures

Height and weight were measured and body mass index (BMI) calculated as weight (kg)/height (m)^2^.

#### Blood samples analyses

Peripheral blood samples were drawn at 8:00 a.m. after an overnight fast. Plasma glucose, glycated hemoglobin (HbA1c), alanine transaminase (ALT), aspartate aminotransferase (AST), cholesterol (CH), high density lipoprotein (HDL), low density lipoprotein (LDL), triglycerides (TG), and blood count were measured by routine analysis. Plasma insulin was measured by ARCHITECT Insulin assay (Abbott Laboratories), a chemiluminescent microparticle immunoassay (CMIA)^11^.

#### Liver biopsy and histology

In subjects with obesity, percutaneous liver biopsies were performed under ultrasonography with 16-gauge biopsy needles. In the control subject group, needle liver biopsies (also 16-gauge biopsy needles) were obtained during laparoscopic cholecystectomy^94^.

All biopsies had length ≥15 mm and contained ≥11 portal areas. Fibrosis stage was classified into five staged from 0 to 4 using the METAVIR scoring system: (F0—no fibrosis, F1—portal fibrosis, F2—periportal fibrosis, F3—bridging fibrosis, F4—cirrhosis), according to Bedossa and Ponyard^95^. Fibrosis and steatosis were also staged according to the system described by Kleiner et al.^96^. and Brunt et al.^97^. NASH was diagnosed in presence of steatosis, lobular inflammation and hepatocyte ballooning with or without peri-sinusoidal fibrosis and NASH activity was graded according to the value of NAFLD Activity Score (NAS). The NAS was calculated by adding the severity scores for steatosis, lobular inflammation, and ballooning with a range from 0 to 8. A NAS=3, resulting from the sum of steatosis = 1, lobular inflammation = 1 and hepatocyte ballooning = 1, was the minimum value to make diagnosis of NASH. The SAF score was also calculated^98^. The SAF scoring system separately assesses the grade of steatosis S from S0 to S3, the activity grade A from A0 to A4 by addition of grades of ballooning and lobular inflammation, each graded from 0 to 2, and the stage of fibrosis F, from F0 to F4 according to NASH Clinical Research Network staging system (CRN).

A single expert hepatopathologist read the digitized slides according to CRN criteria.

#### HSP70 and GRP78 fasting levels changes after RYGB or BPD study

Patients with class III obesity (BMI ≥ 40.0 kg/m2) who were scheduled to undergo Roux-en-Y Gastric Bypass (RYGB) (*n* = 12, 11 men) or Biliopancreatic diversion (BPD) (*n* = 12, 6 men) surgeries at Catholic University in Rome, Italy participated in this study. All subjects completed a medical evaluation, including a history and physical examination and standard blood tests. Many participants in both the RYGB and BPD groups had cardiometabolic diseases: hypercholesterolemia (total cholesterol ≥ 240 mg/dL or LDL-C ≥ 160 mg/dL, or treatment with atorvastatin) in 8 RYGB and 9 BPD participants, hypertension (systolic or diastolic blood pressure > 140/90 mmHg, or treatment with antihypertensive medication) in 7 RYGB and 7 BPD participants, and impaired fasting glucose (plasma glucose ≥ 100 mg/dl) in 7 RYGB and 5 BPD participants. People with diabetes were excluded to avoid the potential confounding effect of diabetes medications and postoperative changes in medications on the outcome measures. In addition, those with previous intestinal surgery or a history of inflammatory intestinal disease, and those who were taking medications that could influence the study outcome measures were excluded.

### ELISA

HSP70 was measured by ELISA (Proteintech, Rosemont, IL, USA) with a sensitivity of 1.0 ng/ml and intra- and interassay CVs were 4.2–6.3% and 5.5–6.1%, respectively. GRP78 was measured by ELISA (Enzo Life Science, Farmingdale, NY) with a sensitivity of 8.4 ng/ml and intra- and interassay CVs were 4.5–14.1% and 4.8–13.1%, respectively.

#### HSP70 and GRP78 kinetic after a mixed meal

HSP70 and GRP78 serum levels were measured before and at various time points after a 413 kcal high-fat meal (42% carbohydrates, 53% fat, and 5% proteins). The mathematical model was the same for rats and humans (see Supplemental Material).

### In vitro studies

Rat hepatocytes were isolated by collagenase perfusion of the liver as described elsewhere^99^. Cells were cultured for 24 h in DMEM with 10% FBS. Prior stimulation, cells were incubated in serum free DMEM overnight.

To assess lipid droplet accumulation, primary rat hepatocytes were stimulated with complete DMEM medium supplemented with HSP70 (15 ng/ml) or GRP78 (3 ng/ml) or Oleic Acid (400 µmol/L) for 24 h. To test the effect of TLR4 inhibition, cells were pretreated with or without TAK-242 (200 nM) for one hour. After the stimulation, half of the cells were stained with Nile Red (100 ng/mL) for 45 min. Stained cells were used for immunofluorescence to quantify the deposition of lipid droplets. Unstained cells were used to assess lipid uptake and liponeogenesis through quantitative real-time PCR. Primer sequences are reported in Supplementary Table S6.

Images of lipid droplets were taken using confocal microscope Nikon A1 RHD25 and NIS-Elements imaging software was used to analyze images.

DMEM and FBS were obtained from Corning (New York, NY), Oleic acid and Nile red were obtained from Merck (Darmstadt, DE) and Plin2 was obtained from Abcam (Cambridge, UK).

#### Hepatic stellate cells

Rat hepatic stellate cells (HSCs) were isolated as described elsewhere^100^.

HSCs were stimulated with complete DMEM medium supplemented with HSP70 (15 ng/ml) or GRP78 (3 ng/ml) for 24 h. TLR4 inhibition in HSCs was performed using the same experimental condition employed for primary hepatocytes. HSCs activation was assessed by flow cytometry and real-time PCR by evaluating alpha-smooth muscle Actin (α-SMA) and Collagen type I alpha 1 (COL1A1) protein and gene expression. α-SMA and COL1A1 antibodies were obtained from Thermo Fisher scientific, (Waltham, MA). Antibody for flow cytometry were diluted 1:50 in phosphate buffered saline (PBS). Primer sequences are reported in Supplementary Table S6. Gating strategy is showed in Supplementary Fig. S17B.

#### Intestinal epithelial cells (colon)

Rat colon mucosal layer was dissected from the specimens and vigorously shaken in Ca2+-Mg2+-free HBSS containing 2 mM EDTA for 20 min at 37 °C. Epithelial cells were purified from the supernatant with a 30% Percoll gradient centrifugation. Purified cells were incubated in DMEM containing 10% (vol/vol) FBS and 1% (vol/vol) penicillin-streptomycin in a 96-well plate at the concentration of 5 × 10^4^ cells/well for 24 h in the presence of HSP70 (15 ng/ml) and GRP78 (3 ng/ml).

#### Statistical analysis

Data are expressed as the mean ± SEM unless otherwise specified. Statistical significance was set at *P* < 0.05 (two-tailed). Heatmaps were used as a graphical representation of quantitative real-time PCR, numerical data were included in Supplementary Tables S7 and S8. The statistical analyses were carried out by the SPSS version 26 software (SPSS Inc., Chicago, IL, USA). The parameters DI, x∞, y∞ of the Disposition Index hyperbole y = DI/(x − x∞) + y∞ were estimated by fitting the data (SI(i), Φ(i)) for each experiment.

### Reporting summary

Further information on research design is available in the Nature Portfolio Reporting Summary linked to this article.

## Supplementary information

Supplementary Information

Reporting Summary

## Data Availability

The authors declare that the data supporting the findings of this study are available within the paper and Supplementary Information. All uncropped immunoblotting images can be found within the source data. The proteomics data generated in this study have been deposited in the ProteomeXchange consortium under the following accession code PASS03793. The high-quality metagenome assembled genomes (MAGs) data for this study have been deposited in the European Nucleotide Archive (ENA) at EMBL-EBI under accession number PRJEB57602. Source data are provided with this paper.

## Source data

Source Data
