# Supplementary material for: Upper gut heat shock proteins HSP70 and GRP78 promote insulin resistance, hyperglycemia, and non-alcoholic steatohepatitis
Source: Nat Commun. 2022 Dec 13;13:7715. doi: 10.1038/s41467-022-35310-5 (PMC9748124; doi:10.1038/s41467-022-35310-5)
Supplement: Supplementary file 1 — Supplementary Information [file 41467_2022_35310_MOESM1_ESM.pdf]

SUPPLEMENTARY INFORMATION

Upper gut heat shock proteins HSP70 and GRP78 promote insulin resistance, hyperglycemia, and non-alcoholic steatohepatitis

[1,2]Giulia Angelini, [3]Lidia Castagneto-Gissey, [4]Serenella Salinari, [5]Alessandro Bertuzzi, [1]Danila Anello, [6]Meenakshi Pradhan, [7,8]Marlen Zschätzsch, [9]Paul Ritter, [10]Carel W. Le Roux, [11]Francesco Rubino, [3]Nicola Basso, [3]Giovanni Casella, [12,13]Stefan R. Bornstein[*], [6]Valentina Tremaroli[*], [1,2,13]Geltrude Mingrone[*]

Affiliations:

[1]Università Cattolica del Sacro Cuore, Rome, Italy
[2]Fondazione Policlinico Universitario A. Gemelli IRCCS, Rome, Italy
[3]Department of Surgical Sciences, Sapienza University of Rome, Rome, Italy
[4] Department of Computer, Control, and Management Engineering "Antonio Ruberti", University of Rome "Sapienza", Rome, Italy
[5]CNR-Institute of Systems Analysis and Computer Science (IASI), Rome, Italy
[6]Wallenberg Laboratory for Cardiovascular and Metabolic Research, Department of Molecular and Clinical Medicine, University of Gothenburg, Gothenburg, Sweden.
[7]Institute of Natural Materials Technology, Faculty of Mechanical Science and Engineering, Technischen Universität Dresden, Dresden, Germany
[8]Biotopa gGmbH, Dresden, Germany
[9]Bruker Daltonics SPR, Hamburg, Germany
[10]Diabetes Complications Research Centre, Conway Institute, University College Dublin, Dublin, Ireland.
[11]Bariatric and Metabolic Surgery; King's College Hospital, London UK
[12]Department of Medicine III, Universitätsklinikum Carl Gustav Carus an der Technischen Universität Dresden, Dresden, Germany
[13]Division of Diabetes & Nutritional Sciences, School of Cardiovascular and Metabolic Medicine & Sciences, King's College London, London, United Kingdom

[*]These Authors jointly supervised this work

SUPPLEMENTARY RESULTS

Validation of mAb binding to native serum HSP70 or GRP78

Amino acid sequences of HSP70 and GRP78 are shown in the Supplementary Fig.S3A,B using the one letter code and numbers indicating amino acid positions, where the part in light blue highlights the amino acids sequence of the epitope recognized by the respective monoclonal antibodies (mAbs). HSP70 (Clone 3A3) mAb recognizes the region located between amino acids 504-617 of HSP70, while GRP78 (Clone 76-E6) mAb recognizes the region located between amino acids 497 and 581.

We aimed to ascertain whether commercial mAbs recognize native epitopes of HSP70 or GRP78 and thus measured the affinity constants of these antibodies for the respective native serum proteins by a combination of strategies including Surface Plasmon Resonance (SPR) and co-immunoprecipitation assay. In fact, for physiological studies, it is imperative that mAbs recognize the target protein in its native conformation.

We wanted to ascertain that mAbs used in our study recognized native epitopes of HSP70 or GRP78 and thus we measured the affinity constant of these antibodies for the respective native serum proteins by SPR using covalently attached mAbs to a dextran sensor surface.

Supplementary Fig.S3C,D shows the fitting curves from independent experiments where various dilutions of HFD rat serum with known concentrations of native proteins by ELISA was titrated with a fixed concentration of anti-HSP70 or anti-GRP78 mAbs.

The equilibrium dissociation constant, (KD=kd/ka) was 0.71×10^{-9} M for anti-HSP70 and 0.58×10^{-9} M for anti-GRP78 mAbs. This indicates that antibodies are of high affinity with sensitivity in picomolar range.

The interaction between mAbs and HSP70 or GRP78 was further confirmed by the immunoprecipitation and immunoblotting assays.

To validate mAb specificity, each mAb was separately incubated with a serum sample containing 13.42 ng of HSP70 and 3.08 ng of GRP78 enabling the antibody to bind the respective serum protein. The antibody/antigen complex was then pulled out of the sample using magnetic beads coated with anti-mouse IgG antibody. Western blot SDS-PAGE shows that the mAbs tested were highly specific (Supplementary Fig.S3E,F) since native HSPs were no more detectable after co-immunoprecipitation.

The validation assay, carried out in triplicate, demonstrated, in fact, that Santa Cruz mAb detected HSP70 at the 70±0.8 kDa and 70±4 kDa band position with recombinant and endogenous proteins, respectively, and this conformed with the expected mass of 70 kDa, as indicated in the manufacturer's technical data sheet. Specific mAb from Santa Cruz detected GRP78 at molecular weight positions of 78±0.7 and 78±0.5 kDa for recombinant protein and serum protein, respectively. This is in close agreement with the reported 78-kDa molecular mass.

To exclude that mAbs were able to bind similar antigenic sites on different proteins, we performed cross-reactivity studies on a serum depleted of either HSP70 or GRP78. After immunoprecipitating HSP70 from serum using its specific antibody, we found that mAb against GRP78 did not cross-react with HSP70 in the precipitate (Supplementary Fig.S3G).

Similarly, after GRP78 immunoprecipitation, mAb against HSP70 was unable to detect GRP78 in the immune precipitate (Supplementary Fig.S3H).

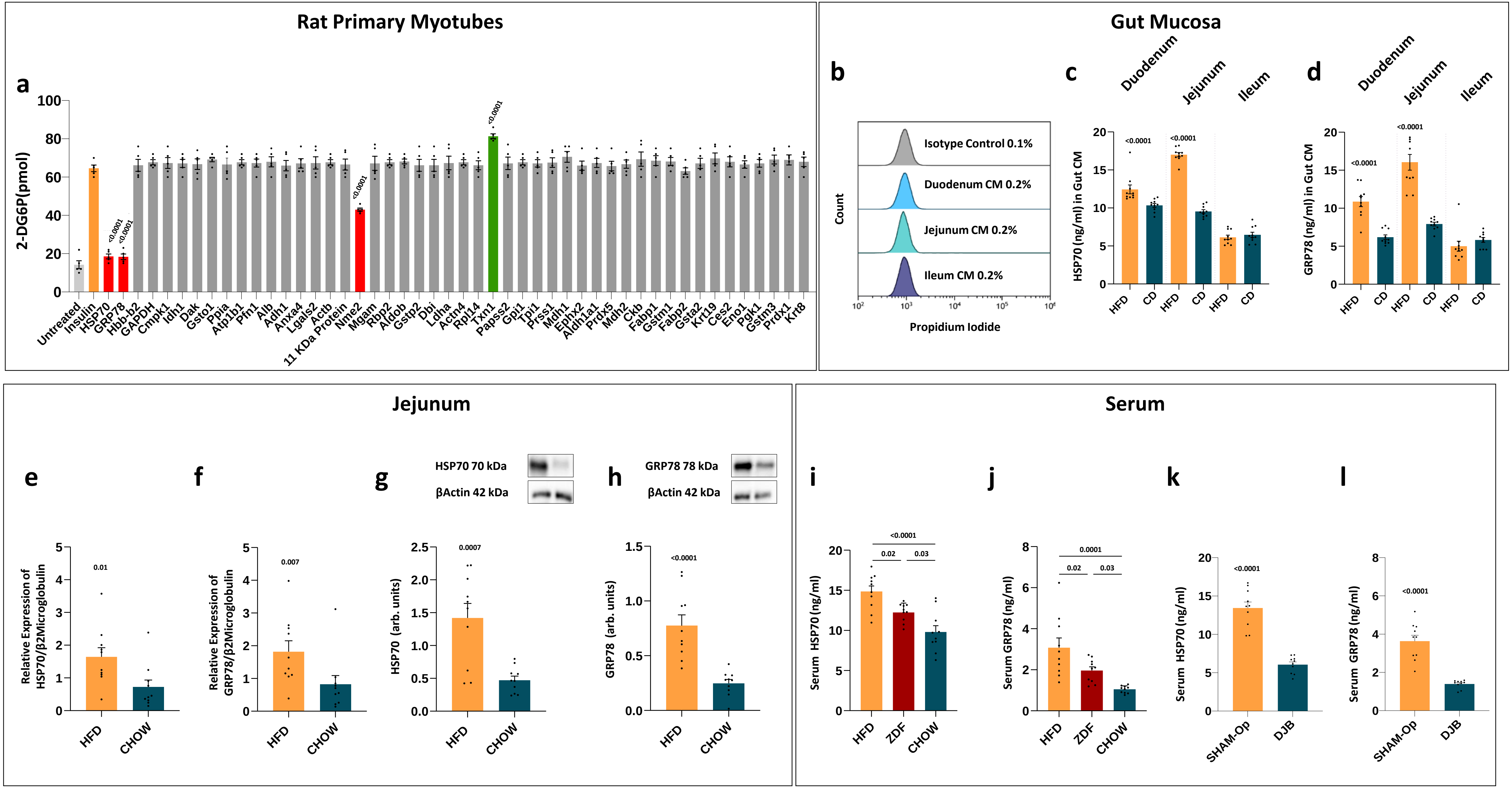

Rat Primary Myotubes
a
2-DG6P(pmol)
Untreated
Insulin
HSP70
GRP78
Hbb-b2
GAPDH
Cmpk1
Idh1
Dak
Gsto1
Ppia
Atp1b1
Pfn1
Alb
Adh1
Anxa4
Lgals2
Actb
11 KDa Protein
Nme2
Mgam
Rbp2
Aldob
Gstp2
Dbi
Ldha
Actn4
Rpl14
Txn1
Papss2
Gpi1
Tpi1
Prss1
Mdh1
Ephx2
Aldh1a1
Prdx5
Mdh2
Ckb
Fabp1
Gstm1
Fabp2
Gsta2
Krt19
Ces2
Eno1
Pgk1
Gstm3
Prdx1
Krt8
<0.0001
Gut Mucosa
b
Isotype Control 0.1%
Duodenum CM 0.2%
Jejunum CM 0.2%
Ileum CM 0.2%
Count
Propidium Iodide
c
Duodenum
Jejunum
Ileum
HSP70 (ng/ml) in Gut CM
HFD
CD
d
GRP78 (ng/ml) in Gut CM
Jejunum
e
Relative Expression of HSP70/β2Microglobulin
0.01
HFD
CHOW
f
Relative Expression of GRP78/β2Microglobulin
0.007
g
HSP70 70 kDa
βActin 42 kDa
HSP70 (arb. units)
0.0007
h
GRP78 78 kDa
βActin 42 kDa
GRP78 (arb. units)
<0.0001
Serum
i
Serum HSP70 (ng/ml)
<0.0001
0.02
0.03
HFD
ZDF
CHOW
j
Serum GRP78 (ng/ml)
0.0001
0.02
0.03
k
Serum HSP70 (ng/ml)
<0.0001
SHAM-Op
DJB
l
Serum GRP78 (ng/ml)
<0.0001

Figure S1.

a, In vitro glucose uptake in rat primary myocytes stimulated with insulin (100 nM) and recombinant proteins (n= 5 biologically independent experiments; most effective concentration selected see Methods). **b,** Flow cytometry-based analysis of intestinal mucosa cell death using propidium iodide staining (n= 5 biologically independent animals). **c,d** In vitro study of the secretion of HSP70 (c) and GRP78 (d) in the conditioned medium (CM) from duodenum, jejunum and ileum in rats fed chow (CD) or high fat diet (HFD) (n= 5 biologically independent animals). **e-h** Intestinal (Jejunum) gene (e,f) and protein (g,h) expression of HSP70 and GRP78 in rats fed a CD or HFD (n= 5 biologically independent animals). **i,j,** Serum concentration of HSP70 (e) and GRP78 (f) in rats under HFD, CD and Zucker Diabetic Fatty (ZDF) rats (n= 5 biologically independent animals). **k,l** Serum concentration of HSP70 (g) and GRP78 (h) in rats that underwent sham-operation (Sham-op) or Duodenal-jejunal Bypass (DJB) (n= 5 biologically independent animals). Data are presented as mean value ± SEM. Statistical significances were calculated by unpaired two-tailed *t*-test, two-tailed Mann–Whitney test and One-way Anova with Bonferroni’s correction for multiple comparisons, where appropriate. Source data are provided as a Source Data file.

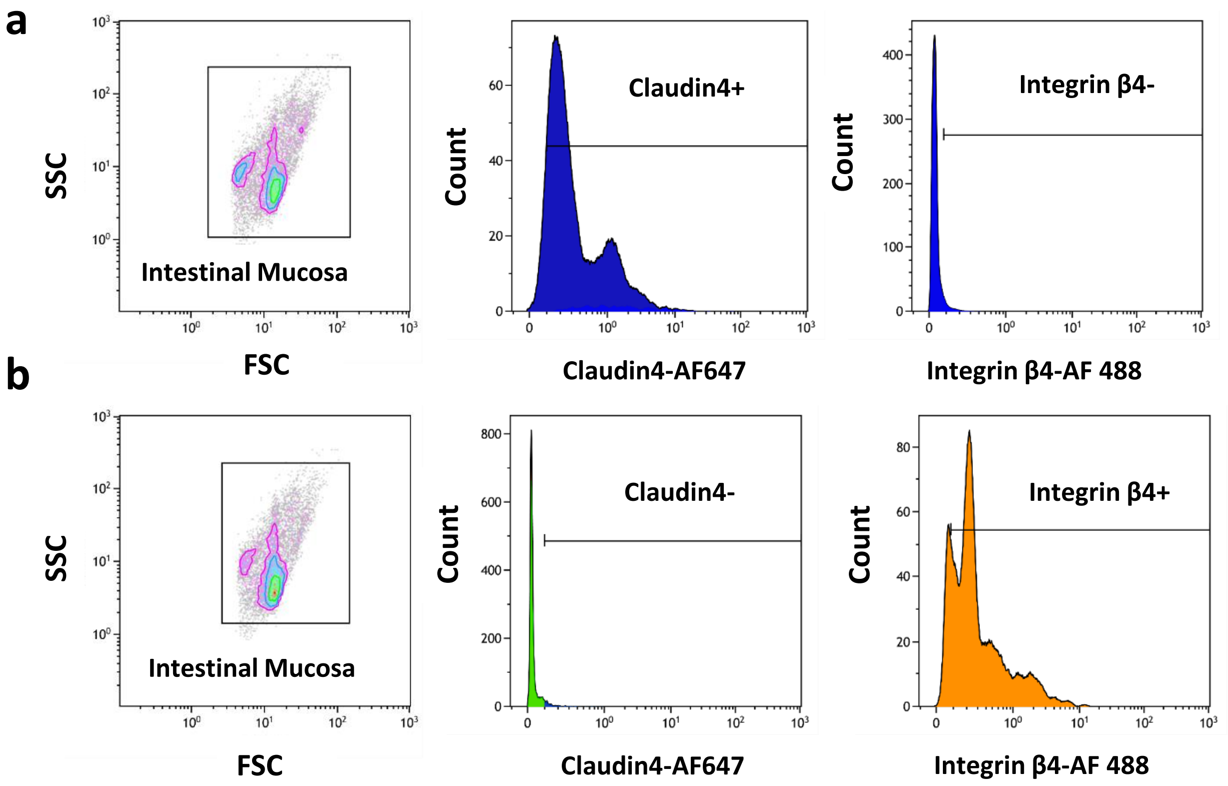
a
b
SSC
FSC
Intestinal Mucosa
Count
Claudin4+
Claudin4-
Claudin4-AF647
Integrin β4-
Integrin β4+
Integrin β4-AF 488

Figure S2.

a,b, Representative image of post-sorting analysis (n= 5 biologically independent animals). After sorting, enteroendocrine cells (a) purity was >90% as shown by the high expression of Claudin-4 and marginal expression of Integrin-β4, while, intestinal epithelial cells (b) purity was >85% since the large majority of cells expressed Integrin-β4.

a

HSP70 (3A3) mAb

```
        10         20         30         40         50
MAKAAAIGID LGTTYSCVGV FQHGKVEIIA NDQGNRTTPS YVAFTDTERL
        60         70         80         90        100
IGDAAKNQVA LNPQNTVFDA KRLIGRKFGD PVVQSDMKHW PFQVINDGDK
       110        120        130        140        150
PKVQVSYKGE TKAFYPEEIS SMVLTKMKEI AEAYLGYPVT NAVITVPAYF
       160        170        180        190        200
NDSQRQATKD AGVIAGLNVL RIINEPTAAA IAYGLDRTGK GERNVLIFDL
       210        220        230        240        250
GGGTFDVSIL TIDDGIFEVK ATAGDTHLGG EDFDNRLVNH FVEEFKRKHK
       260        270        280        290        300
KDISQNKRAV RRLRTACERA KRTLSSSTQA SLEIDSLFEG IDFYTSITRA
       310        320        330        340        350
RFEELCSDLF RSTLEPVEKA LRDAKLDKAQ IHDLVLVGGS TRIPKVQKLL
       360        370        380        390        400
QDFFNGRDLN KSINPDEAVA YGAAVQAAIL MGDKSENVQD LLLLDVAPLS
       410        420        430        440        450
LGLETAGGVM TALIKRNSTI PTKQTQIFTT YSDNQPGVLI QVYEGERAMT
       460        470        480        490        500
KDNNLLGRFE LSGIPPAPRG VPQIEVTFDI DANGILNVTA TDKSTGKANK
       510        520        530        540        550
ITITNDKGRL SKEEIERMVQ EAEKYKAEDE VQRERVSAKN ALESYAFNMK
       560        570        580        590        600
SAVEDEGLKG KISEADKKKV LDKCQEVISW LDANTLAEKD EFEHKRKELE
       610        620        630        640
QVCNPIISGL YQGAGGPGPG GFGAQGPKGG SGSGPTIEEV D
```

b

GRP78 (76-E6) mAb

```
        10         20         30         40         50
MMKFTVVAAA LLLLGAVRAE EEDKKEDVGT VVGIDLGTTY SCVGVFKNGR
        60         70         80         90        100
VEIIANDQGN RITPSYVAFT PEGERLIGDA AKNQLTSNPE NTVFDAKRLI
       110        120        130        140        150
GRTWNDPSVQ QDIKFLPFKV VEKKTKPYIQ VDIGGGQTKT FAPEEISAMV
       160        170        180        190        200
LTKMKETAEA YLGKKVTHAV VTVPAYFNDA QRQATKDAGT IAGLNVMRII
       210        220        230        240        250
NEPTAAAIAY GLDKREGEKN ILVFDLGGGT FDVSLLTIDN GVFEVVATNG
       260        270        280        290        300
DTHLGGEDFD QRVMEHFIKL YKKKTGKDVR KDNRAVQKLR REVEKAKRAL
       310        320        330        340        350
SSQHQARIEI ESFFEGEDFS ETLTRAKFEE LNMDLFRSTM KPVQKVLEDS
       360        370        380        390        400
DLKKSDIDEI VLVGGSTRIP KIQQLVKEFF NGKEPSRGIN PDEAVAYGAA
       410        420        430        440        450
VQAGVLSGDQ DTGDLVLLDV CPLTLGIETV GGVMTKLIPR NTVVPTKKSQ
       460        470        480        490        500
IFSTASDNQP TVTIKVYEGE RPLTKDNHLL GTFDLTGIPP APRGVPQIEV
       510        520        530        540        550
TFEIDVNGIL RVTAEDKGTG NKNKITITND QNRLTPEEIE RMVNDAEKFA
       560        570        580        590        600
EEDKKLKERI DTRNELESYA YSLKNQIGDK EKLGGKLSSE DKETMEKAVE
       610        620        630        640        650
EKIEWLESHQ DADIEDFKAK KKELEEIVQP IISKLYGSGG PPPTGEEDTS

EKDEL
```

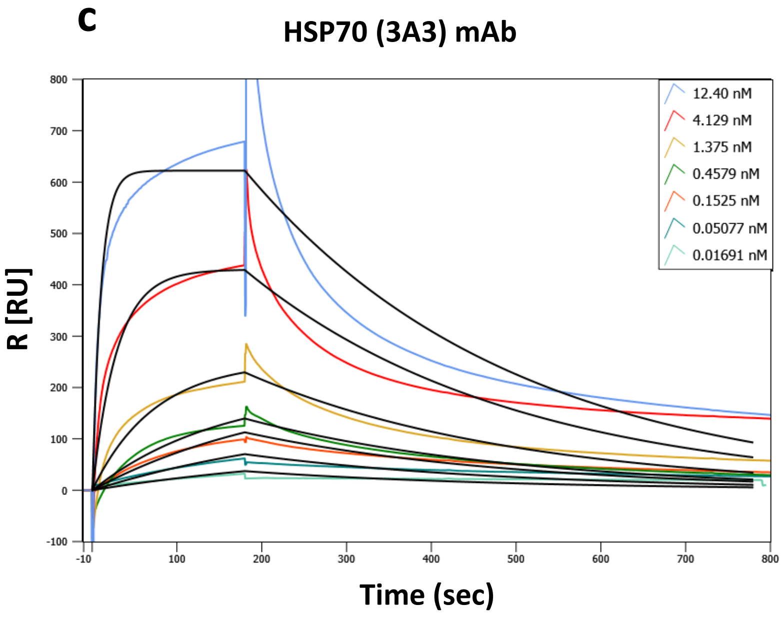

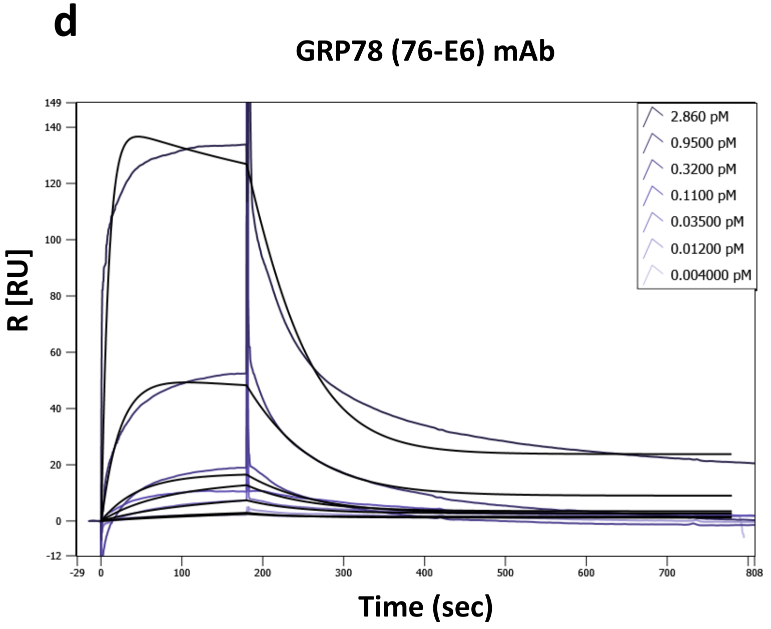

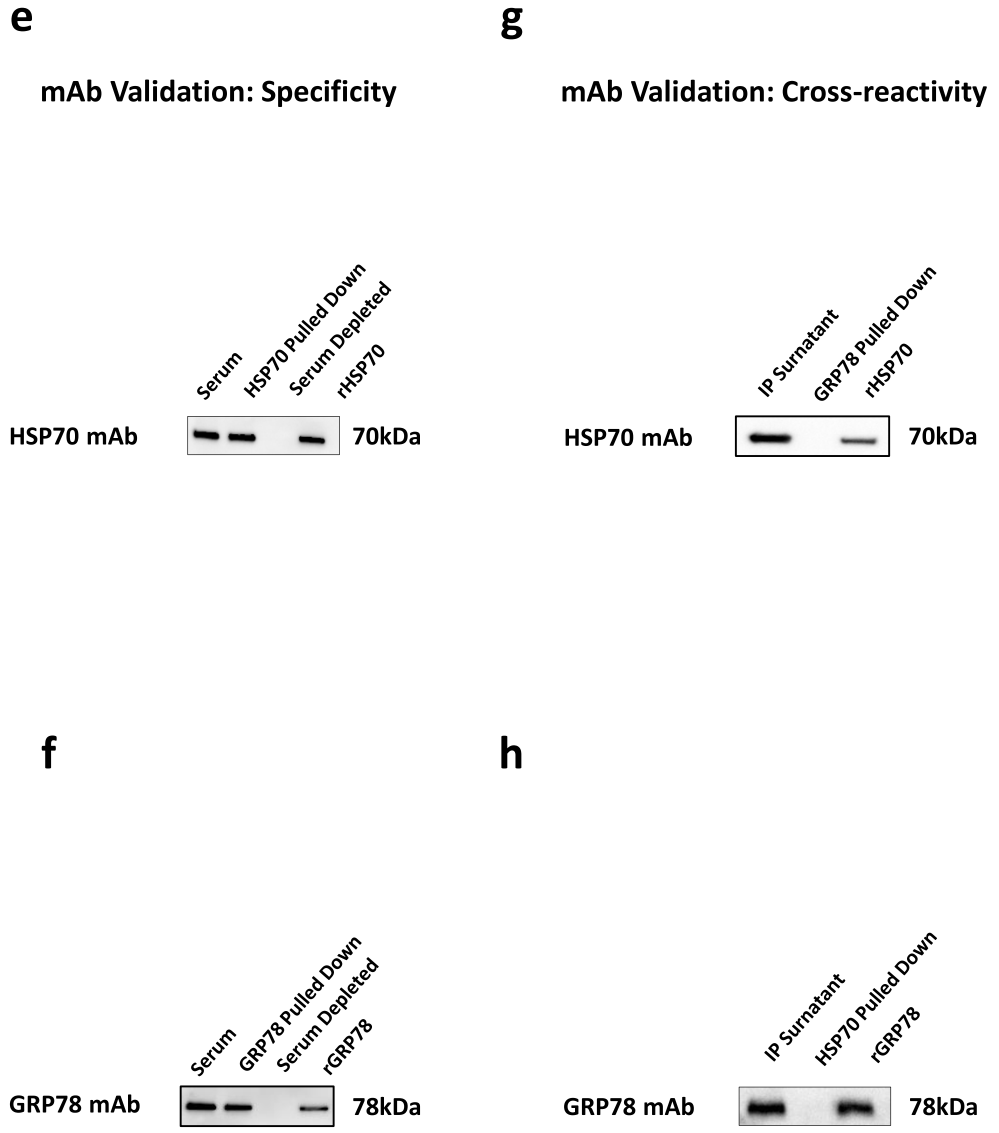

Figure S3.

a,b, Amino acid sequence of HSP70 (a) and GRP78 (b) using the one letter code and numbers indicating amino acid positions; the part in light blue highlights the amino acids sequence of the epitope recognized by the respective monoclonal antibodies (mAbs). HSP70 (Clone 3A3) mAb recognizes the region located between amino acids 504-617 of HSP70, while GRP78 (Clone 76-E6) mAb recognizes the region located between amino acids 497 and 581. **c,d,** Sensorgram of Surface Plasmon Resonance evaluating the binding affinity between mAbs against HSP70 (c) or GRP78 (d) to serum HSP70 and GRP78. **e-h,** Antibodies specificity (e,f) and cross- reactivity (g,h) validation by co-immunoprecipitation and Western Blot. N=5 indipendent experiments.

a **HSP70 (3A3) mAb**

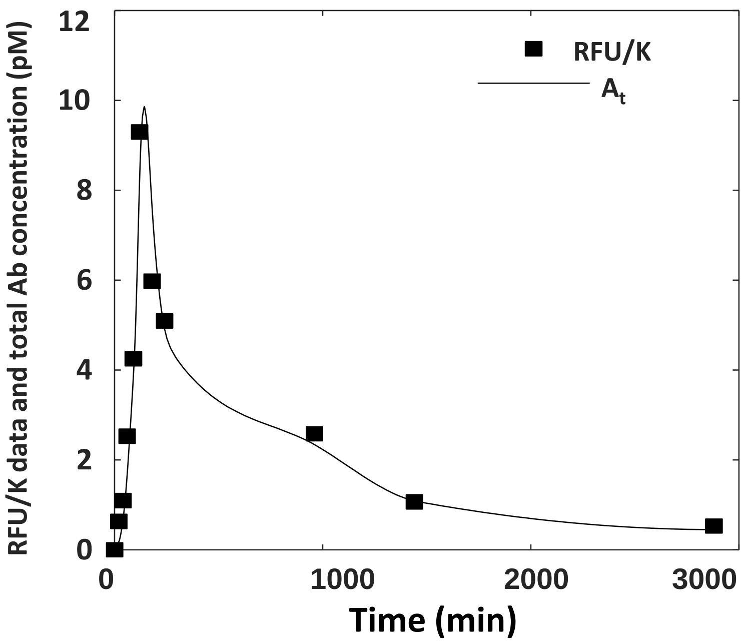

V =0.044 (l)
$\bar{P}$ =211 (pM)
K =1.38 (pM^{-1})
K_{SS} =752 (10^{-12} M)
k_e=0.055 (h^{-1})
k_{int} =0.057 (h^{-1})

b **GRP78 (76-E6) mAb**

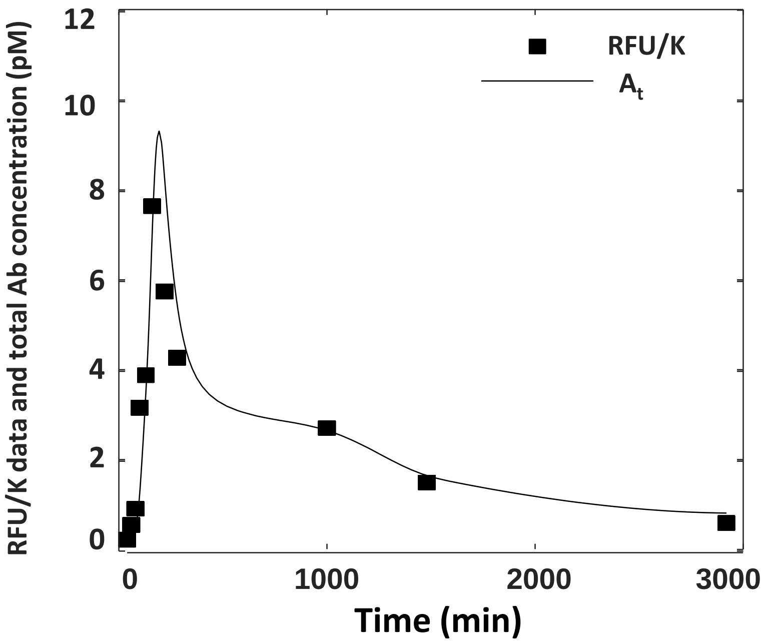

V =0.051 (l)
$\bar{P}$ =42.1 (pM)
K =1.63(pM^{-1})
K_{SS} =608 (10^{-12} M)
k_e=0.018(h^{-1})
k_{int} =0.017 (h^{-1})

Figure S4.

Time-course of the Relative Fluorescent Units (RFU) of mAbs against HSP70 (a) and GRP78 (b). RFU is represented by filled squares, while the model fitting is represented by a continuous line. The model fitting provides the mAb concentration in pM. V (l) is the mAb distribution volume. $\bar{P}$ (pM) is the free HSP serum concentration. K (pM^{-1}) is a constant proportional to mAb total concentration. K_{SS} (10^{-12} M) is the quasi-steady state dissociation constant of the mAb-HSP complex. The elimination of mAb from serum is represented by k_e (h^{-1}). Finally, the mAb internalization into tissues is represented by the constant rate k_{int} (h^{-1}).

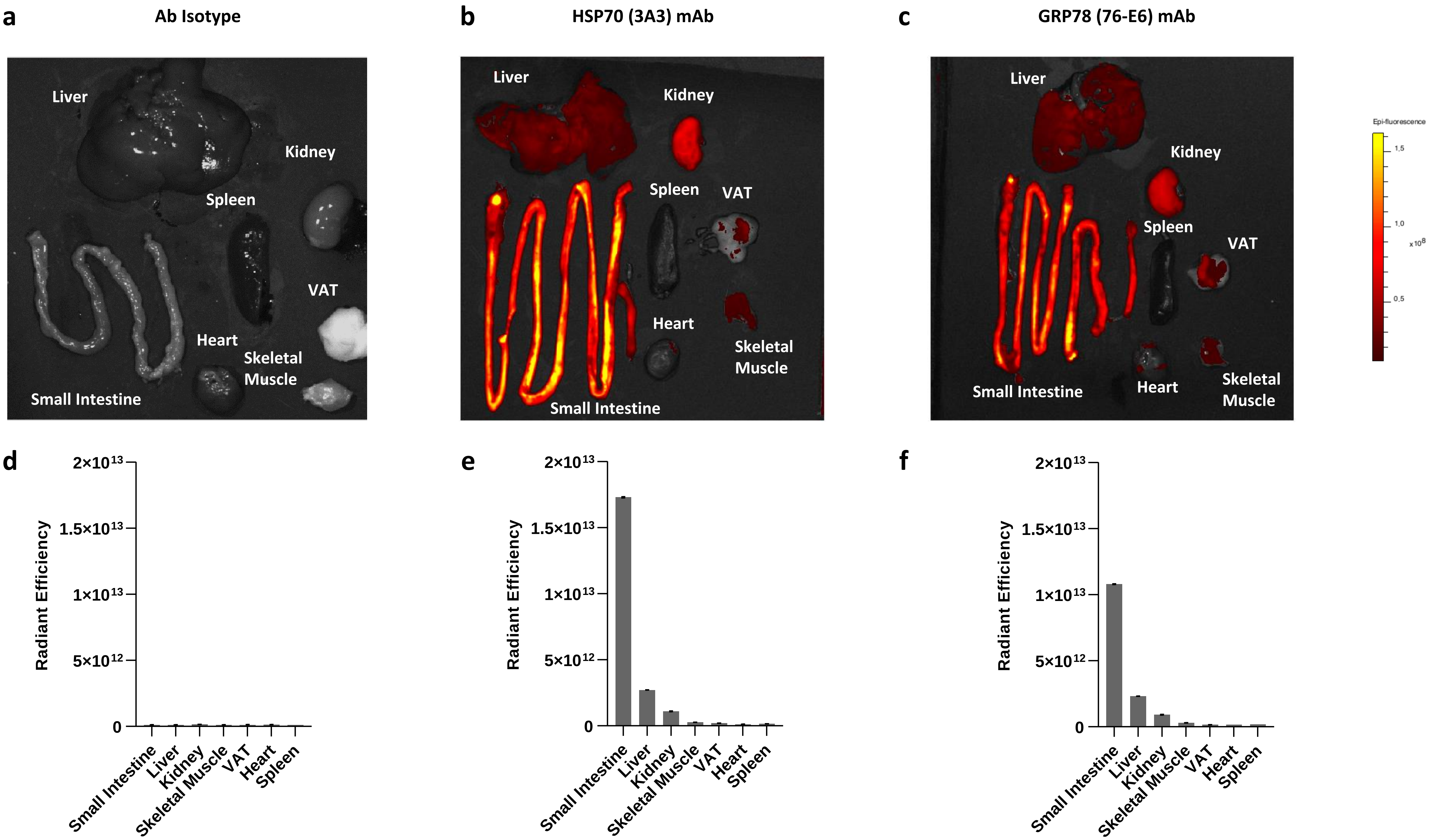

a
Ab Isotype
b
HSP70 (3A3) mAb
c
GRP78 (76-E6) mAb
Liver
Kidney
Spleen
VAT
Heart
Skeletal Muscle
Small Intestine
Epi-fluorescence
1,5
1,0
0,5
x10^8
d
e
f
Radiant Efficiency
2×10^13
1.5×10^13
1×10^13
5×10^12
0
Small Intestine
Liver
Kidney
Skeletal Muscle
VAT
Heart
Spleen

Figure S5.

a-c, Representative *ex vivo* images of dissected organs 2 hours after the injection (1µg/ml) of fluorescent Ab isotype (a) or mAbs against HSP70 (b) or GRP78 (c). **c,d,** Radiant efficiency of the tissues after the infusion with Ab isotype (d) or mAbs against HSP70 (e) or GRP78 (f). Data are presented as mean value ± SEM of n= 5 biologically independent animals. Source data are provided as a Source Data file.

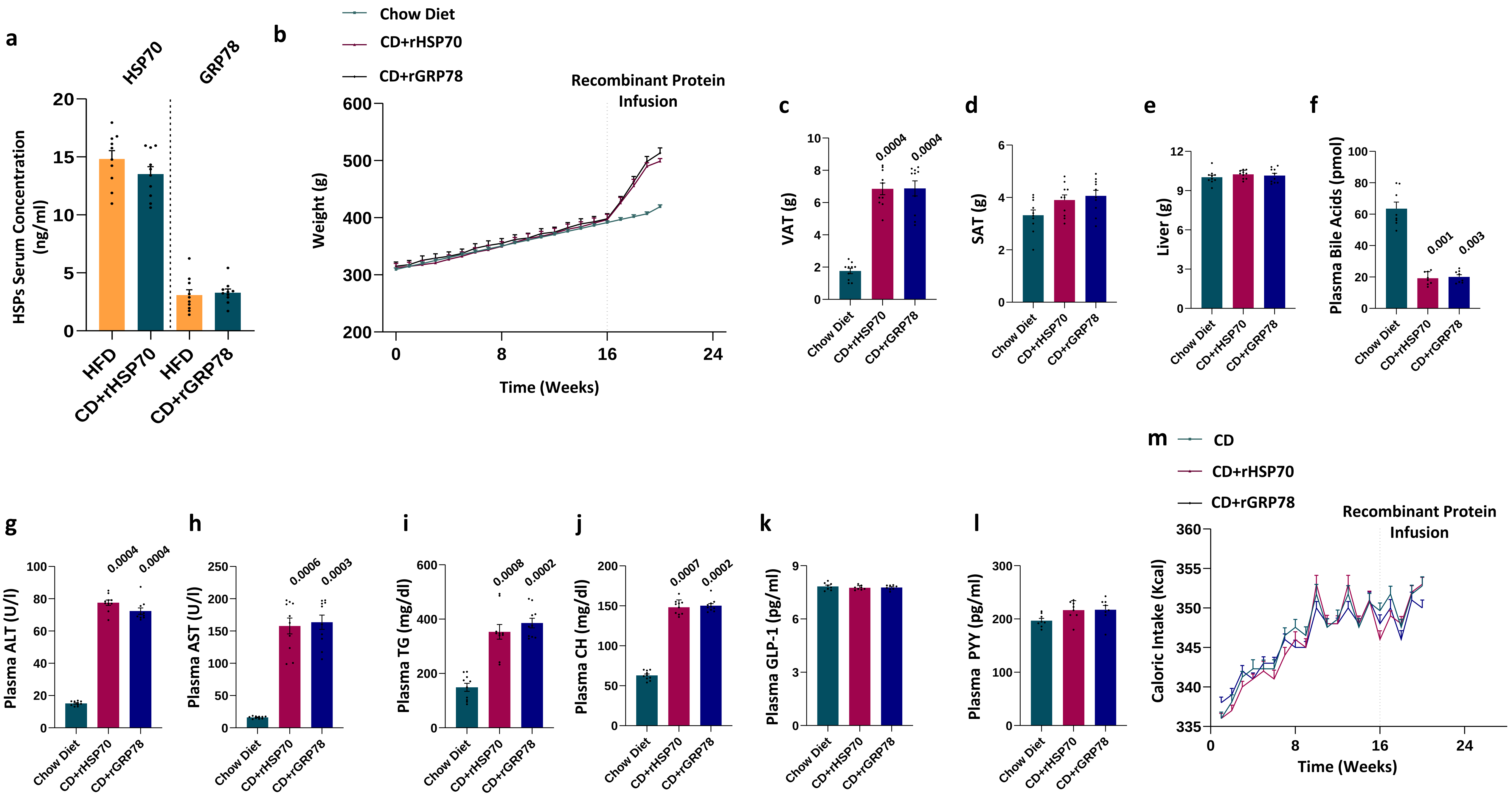

a
HSP70
GRP78
HSPs Serum Concentration (ng/ml)
HFD
CD+rHSP70
HFD
CD+rGRP78
b
Chow Diet
CD+rHSP70
CD+rGRP78
Recombinant Protein Infusion
Weight (g)
Time (Weeks)
c
VAT (g)
0.0004
0.0004
d
SAT (g)
e
Liver (g)
f
Plasma Bile Acids (pmol)
0.001
0.003
Chow Diet
CD+rHSP70
CD+rGRP78
g
Plasma ALT (U/l)
0.0004
0.0004
h
Plasma AST (U/l)
0.0006
0.0003
i
Plasma TG (mg/dl)
0.0008
0.0002
j
Plasma CH (mg/dl)
0.0007
0.0002
k
Plasma GLP-1 (pg/ml)
l
Plasma PYY (pg/ml)
m
CD
CD+rHSP70
CD+rGRP78
Recombinant Protein Infusion
Caloric Intake (Kcal)
Time (Weeks)

Figure S6.

a, Serum concentration of HSP70 and GRP78 in rats under chow diet in combination with continuous infusion of recombinant HSP70 or GRP78. **b**, Average body weight time course; the dotted line represents the time when the osmotic pumps were implanted to infuse recombinant HSP70 or GRP78 or saline solution. **c-e,** Body composition; weight of visceral adipose tissue (VAT) (c), subcutaneous adipose tissue (SAT) (d) and liver (e). **f-l,** Plasma levels of total Bile Acids (f), Alanine aminotransferase (ALT) (g), Aspartate aminotransferase (AST) (h), Triglycerides (TG) (i), Cholesterol (CH) (j), Glucagon-like peptide-1 (GLP-1) (k) and Peptide YY (PYY) (l) in rats fed a chow diet (CD) and infused with recombinant HSP70 or GRP78 or saline solution. **m,** Time course of caloric intake; the dotted line represents the time when the osmotic pumps were implanted to infuse recombinant HSP70 or GRP78 or saline solution. Data are mean±SEM from n=10 biologically independent animals. Statistical significances were calculated by Kruskal–Wallis test with Dunnett's correction for multiple comparisons. Source data are provided as a Source Data file.

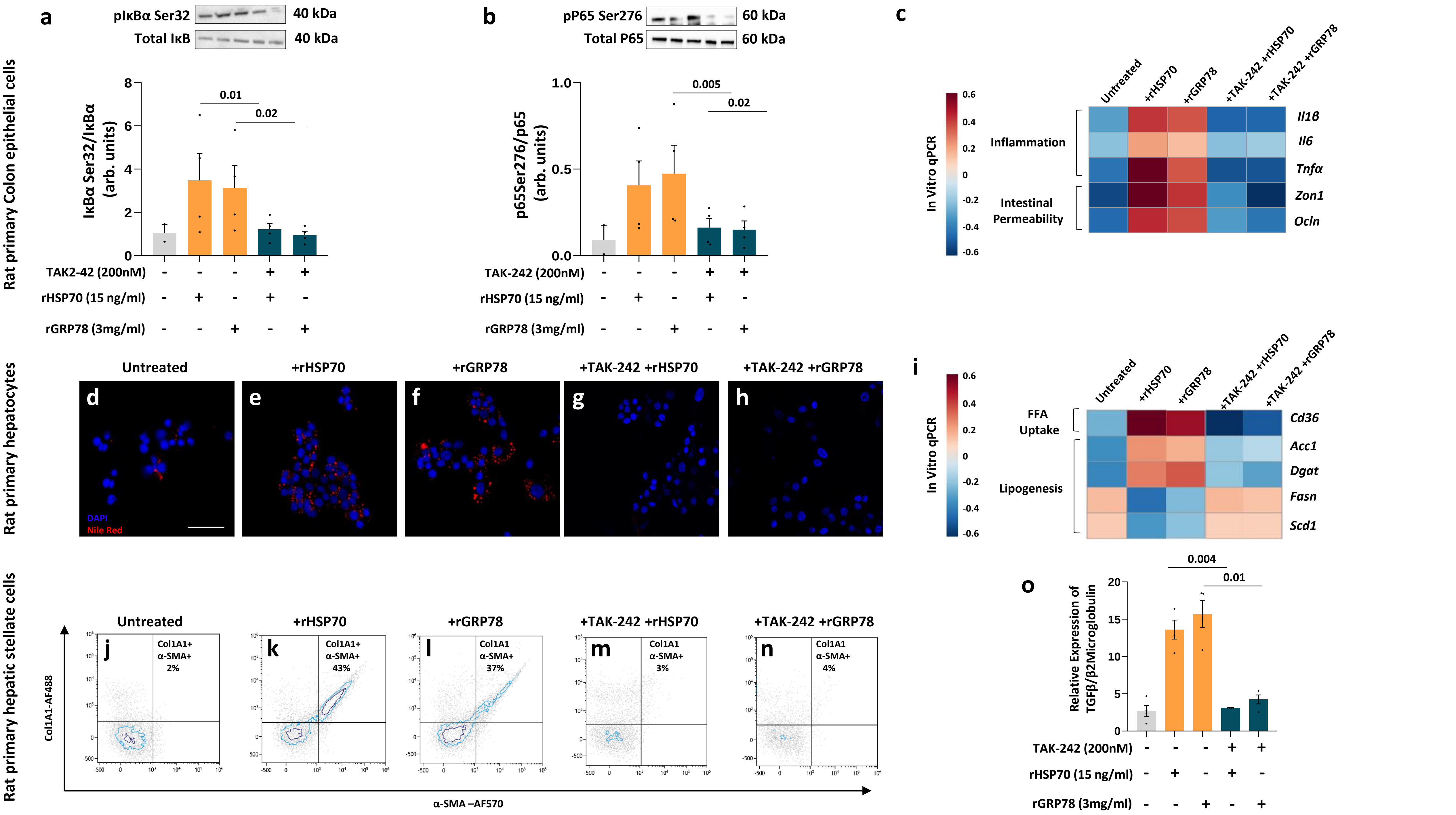
a
pIκBα Ser32
40 kDa
Total IκB
40 kDa
Rat primary Colon epithelial cells
IκBα Ser32/IκBα (arb. units)
8
6
4
2
0
0.01
0.02
TAK2-42 (200nM) - - - + +
rHSP70 (15 ng/ml) - + - + -
rGRP78 (3mg/ml) - - + - +
b
pP65 Ser276
60 kDa
Total P65
60 kDa
p65Ser276/p65 (arb. units)
1.0
0.5
0.0
0.005
0.02
TAK-242 (200nM) - - - + +
rHSP70 (15 ng/ml) - + - + -
rGRP78 (3mg/ml) - - + - +
c
In Vitro qPCR
0.6
0.4
0.2
0
-0.2
-0.4
-0.6
Untreated
+rHSP70
+rGRP78
+TAK-242 +rHSP70
+TAK-242 +rGRP78
Inflammation
Intestinal Permeability
Il1β
Il6
Tnfα
Zon1
Ocln
Rat primary hepatocytes
Untreated
+rHSP70
+rGRP78
+TAK-242 +rHSP70
+TAK-242 +rGRP78
d
e
f
g
h
DAPI
Nile Red
i
In Vitro qPCR
0.6
0.4
0.2
0
-0.2
-0.4
-0.6
Untreated
+rHSP70
+rGRP78
+TAK-242 +rHSP70
+TAK-242 +rGRP78
FFA Uptake
Lipogenesis
Cd36
Acc1
Dgat
Fasn
Scd1
Rat primary hepatic stellate cells
Untreated
+rHSP70
+rGRP78
+TAK-242 +rHSP70
+TAK-242 +rGRP78
j
Col1A1+ α-SMA+ 2%
k
Col1A1+ α-SMA+ 43%
l
Col1A1 α-SMA+ 37%
m
Col1A1 α-SMA+ 3%
n
Col1A1 α-SMA+ 4%
Col1A1-AF488
α-SMA –AF570
o
Relative Expression of TGFβ/β2Microglobulin
20
15
10
5
0
0.004
0.01
TAK-242 (200nM) - - - + +
rHSP70 (15 ng/ml) - + - + -
rGRP78 (3mg/ml) - - + - +

Figure S7.

a,b, Intestinal (rat primary colon epithelial cells) phosphorylation of phospho-inhibitory subunit of NF-κB alpha (pIκBα) on Ser32 and Nuclear factor NF-kappa-B p65 subunit (p65) on Ser276 in the presence of recombinant heat-shock proteins (15 ng/ml of HSP70 and 3 ng/ml of GRP78) and in the presence or absence of TLR4 inhibitor, TAK-242 (200nM). **c**, Gene expression of intestinal (rat primary colon epithelial cells) inflammatory markers as well as key regulators of intestinal permeability. **d-h**, Nile red staining of primary rat hepatocytes incubated with recombinant heat-shock proteins (15 ng/ml of HSP70 and 3 ng/ml of GRP78) and in the presence or absence of TAK-242 (200nM). **i,** Gene expression of free-fatty acid transporter CD36 and key enzymes involved in hepatic de novo lipogenesis. **j-n,** Flow cytometry of myofibroblastic differentiation of rat primary hepatic stellate cells treated with recombinant heat-shock proteins (15 ng/ml of HSP70 and 3 ng/ml of GRP78) and in the presence or absence of TLR4 inhibitor, TAK- 242 (200nM). **o,** Gene expression of Tumor growth factor β (TGF-β) in rat primary hepatic stellate cells treated with recombinant HSP70 (15 ng/ml) or GRP78 (3 ng/ml) and in the presence or absence of TAK-242 (200nM). Magnification 40X. Scale bar: 50 μm. Data are mean ± SEM of n=4 independent experiments. Statistical significances were calculated by paired two-tailed *t*-test. Source data are provided as a Source Data file.

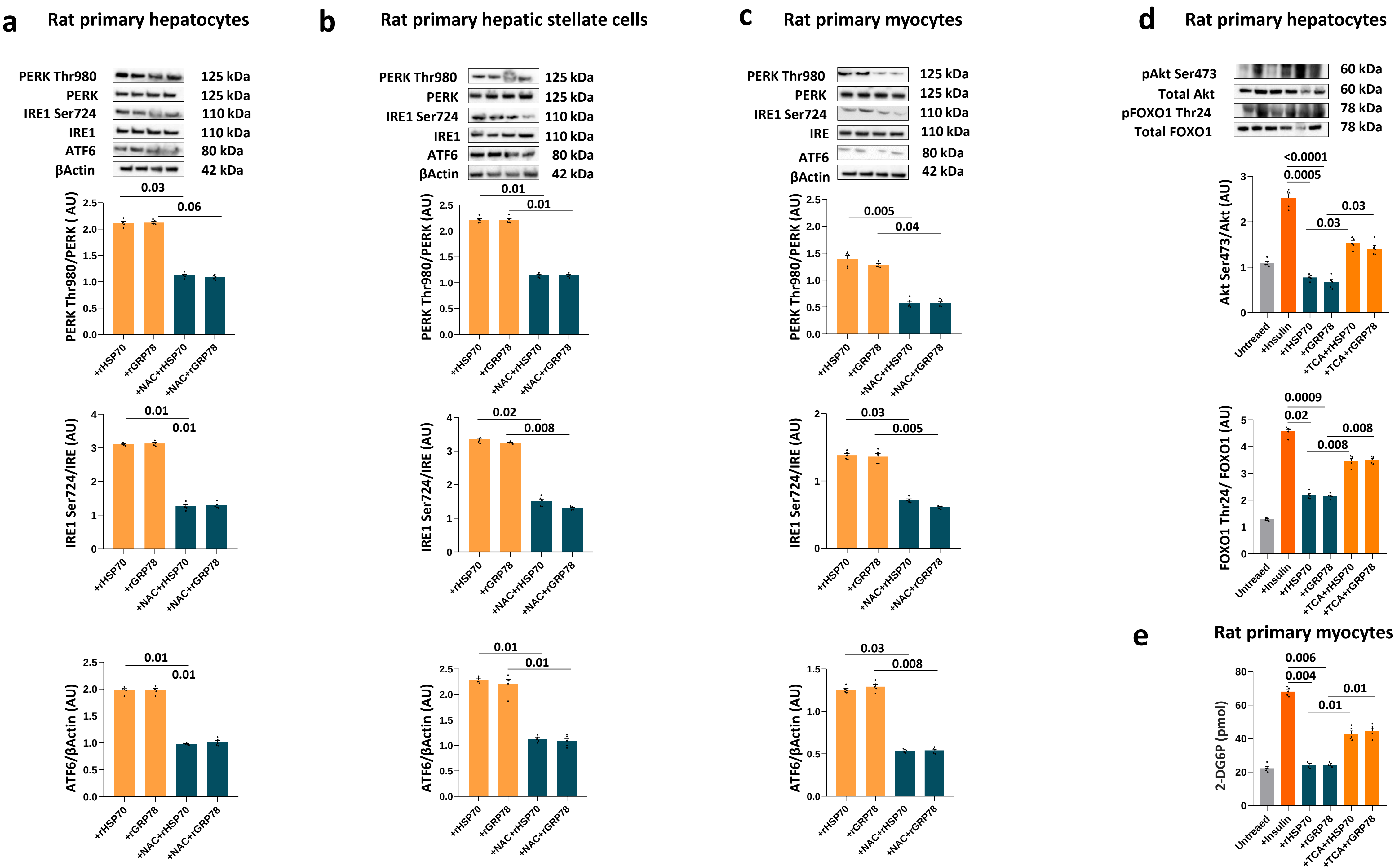
a
Rat primary hepatocytes
PERK Thr980
PERK
IRE1 Ser724
IRE1
ATF6
βActin
125 kDa
125 kDa
110 kDa
110 kDa
80 kDa
42 kDa
0.03
0.06
PERK Thr980/PERK (AU)
0.01
0.01
IRE1 Ser724/IRE (AU)
0.01
0.01
ATF6/βActin (AU)
+rHSP70
+rGRP78
+NAC+rHSP70
+NAC+rGRP78
b
Rat primary hepatic stellate cells
PERK Thr980
PERK
IRE1 Ser724
IRE1
ATF6
βActin
125 kDa
125 kDa
110 kDa
110 kDa
80 kDa
42 kDa
0.01
0.01
PERK Thr980/PERK (AU)
0.02
0.008
IRE1 Ser724/IRE (AU)
0.01
0.01
ATF6/βActin (AU)
c
Rat primary myocytes
PERK Thr980
PERK
IRE1 Ser724
IRE
ATF6
βActin
125 kDa
125 kDa
110 kDa
110 kDa
80 kDa
42 kDa
0.005
0.04
PERK Thr980/PERK (AU)
0.03
0.005
IRE1 Ser724/IRE (AU)
0.03
0.008
ATF6/βActin (AU)
d
Rat primary hepatocytes
pAkt Ser473
Total Akt
pFOXO1 Thr24
Total FOXO1
60 kDa
60 kDa
78 kDa
78 kDa
<0.0001
0.0005
0.03
0.03
Akt Ser473/Akt (AU)
0.0009
0.02
0.008
0.008
FOXO1 Thr24/ FOXO1 (AU)
Untreaed
+Insulin
+rHSP70
+rGRP78
+TCA+rHSP70
+TCA+rGRP78
e
Rat primary myocytes
0.006
0.004
0.01
0.01
2-DG6P (pmol)

Figure S8.

a-c, Western Blot analysis of key proteins involved in Unfolded Protein Response (UPR). Rat primary hepatocytes (a), rat primary hepatic stellate cells (b) and rat primary myocytes (c) were treated with recombinant heat-shock proteins (15 ng/ml of HSP70 and 3 ng/ml of GRP78), in the presence or absence of N-acetyl cysteine (NAC) (50µM). **d,** Western Blot analysis of insulin mediated Akt Ser473 and Foxo1 Thr24 phosphorylation in rat primary hepatocytes treated with recombinant heat-shock proteins (15 ng/ml of HSP70 and 3 ng/ml of GRP78), in the presence or absence of Taurocholate (TCA) (1 mM). Hepatocytes were stimulated with 100nM insulin for 10 minutes. **e,** Insulin mediated glucose uptake of rat primary myocytes treated with HSP70 (15 ng/ml), GRP78 (3ng/ml) alone or in combination with Taurocholate (1 mM). Myocyte were stimulated with 100nM insulin for 10 minutes. Data are mean ± SEM of n=4 independent experiments. Statistical significances were calculated by Kruskal–Wallis test with Dunnett's correction for multiple comparisons. Source data are provided as a Source Data file.

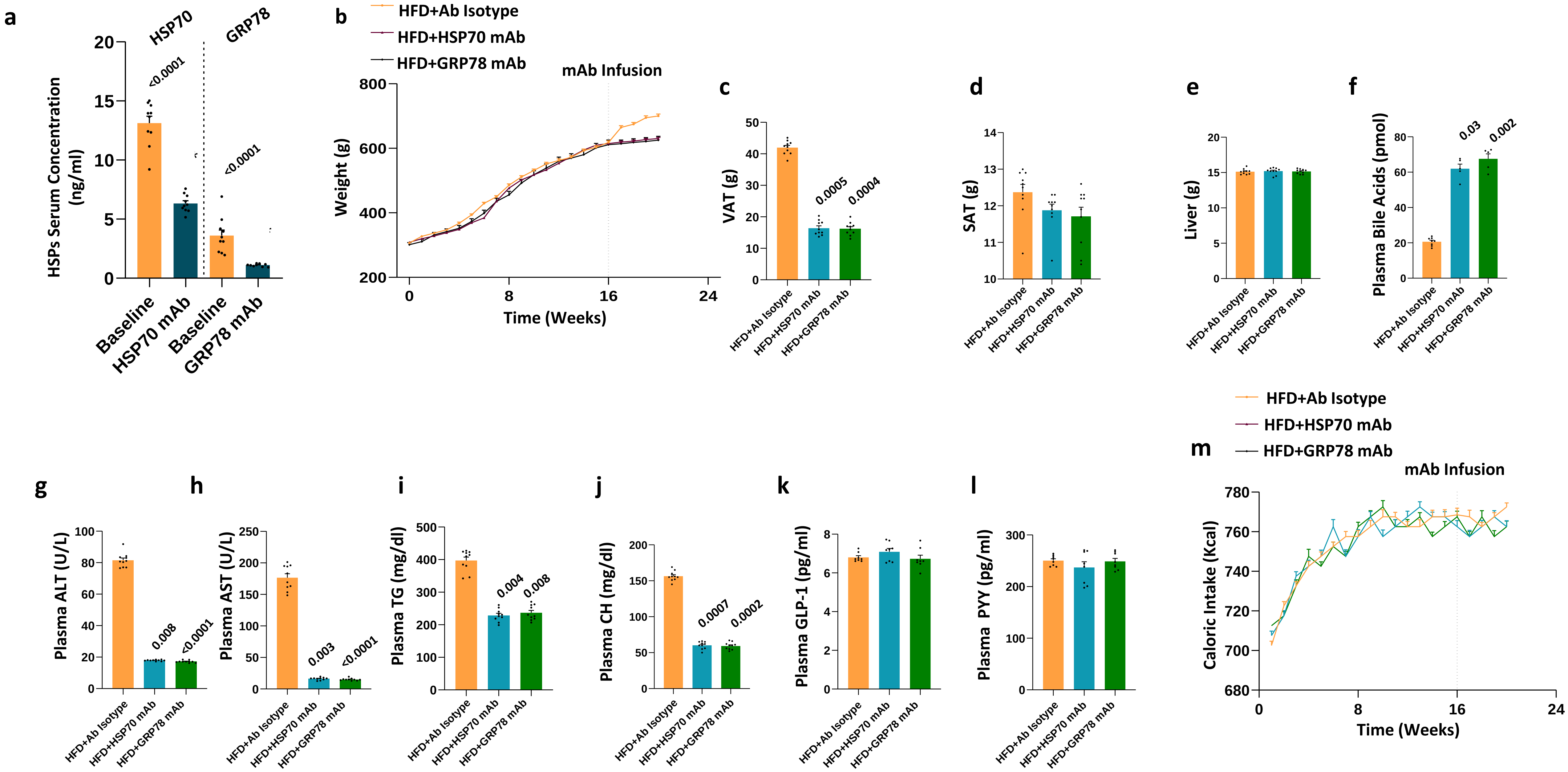
a
HSP70
GRP78
HSPs Serum Concentration (ng/ml)
<0.0001
<0.0001
Baseline
HSP70 mAb
Baseline
GRP78 mAb
b
HFD+Ab Isotype
HFD+HSP70 mAb
HFD+GRP78 mAb
mAb Infusion
Weight (g)
Time (Weeks)
c
VAT (g)
0.0005
0.0004
d
SAT (g)
e
Liver (g)
f
Plasma Bile Acids (pmol)
0.03
0.002
g
Plasma ALT (U/L)
0.008
<0.0001
h
Plasma AST (U/L)
0.003
<0.0001
i
Plasma TG (mg/dl)
0.004
0.008
j
Plasma CH (mg/dl)
0.0007
0.0002
k
Plasma GLP-1 (pg/ml)
l
Plasma PYY (pg/ml)
m
HFD+Ab Isotype
HFD+HSP70 mAb
HFD+GRP78 mAb
mAb Infusion
Caloric Intake (Kcal)
Time (Weeks)

Figure S9.

a, Serum concentrations of HSP70 and GRP78 at baseline and following mAbs infusion. **b,** Average body weight time-course; the dotted line represents the time when the osmotic pumps were implanted to infuse mAbs against HSP70 or GRP78 or Ab Isotype. **c-e,** Body composition; weight of visceral adipose tissue (VAT) (c), subcutaneous adipose tissue (SAT) (d), liver weight (e). **f-l,** Plasma levels of total Bile Acids (f), Alanine aminotransferase (ALT) (g), Aspartate aminotransferase (AST) (h), Triglycerides (TG) (i), Cholesterol (CH) (j), Glucagon-like peptide-1 (GLP-1) (k) and Peptide YY (PYY) (l)in rats fed a high fat diet (HFD) and infused with mAbs against HSP70 or GRP78 or Ab Isotype. **l,** Time course of caloric intake; the dotted line represents the time when the osmotic pumps were implanted to infuse mAbs against HSP70 or GRP78 or Ab Isotype. Data are mean ± SEM from n=10 biologically independent animals. Statistical significances were calculated by Kruskal–Wallis test with Dunnett's correction for multiple comparisons and two-tailed Mann–Whitney test. Source data are provided as a Source Data file.

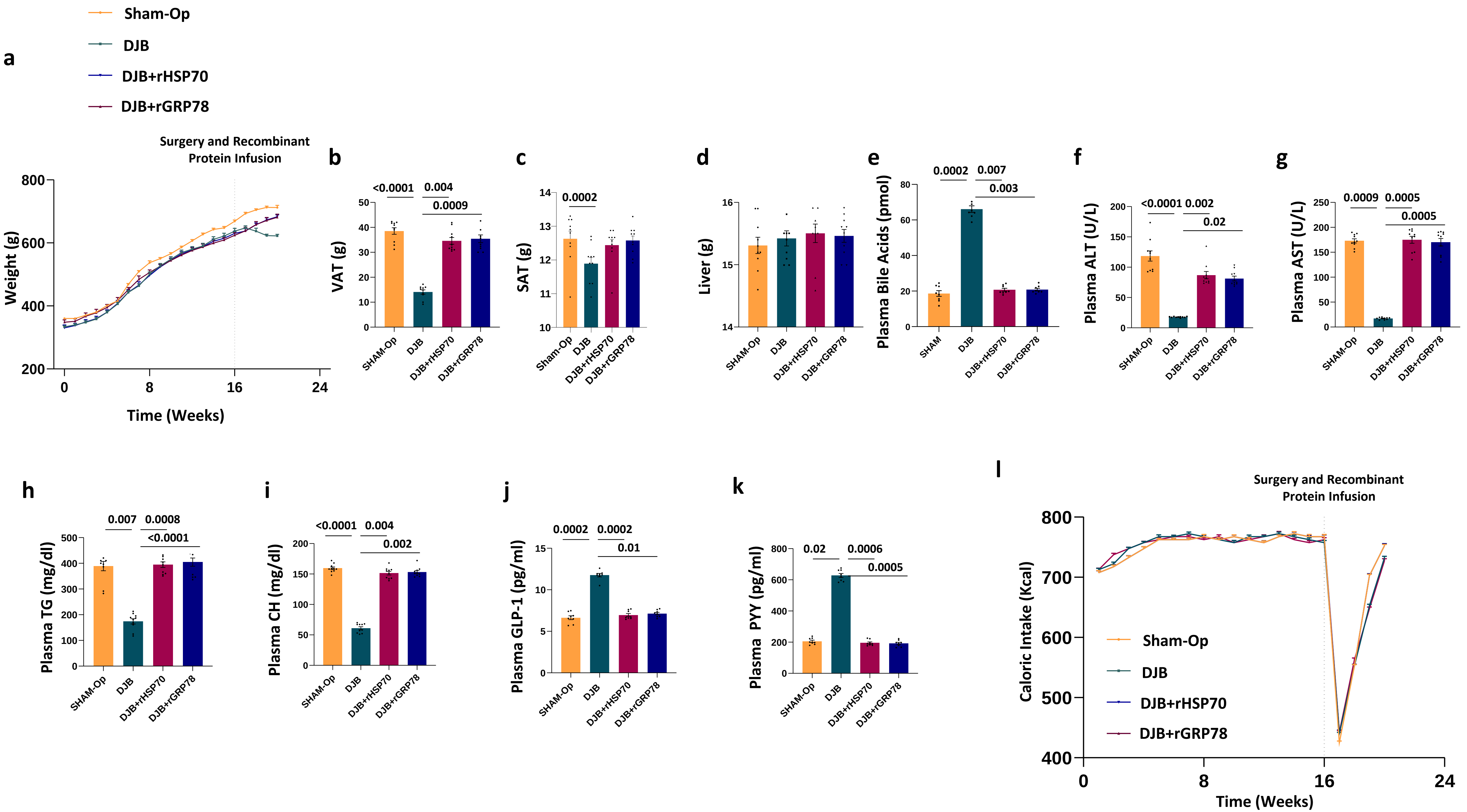

Sham-Op
DJB
DJB+rHSP70
DJB+rGRP78
a
b
c
d
e
f
g
h
i
j
k
l
Surgery and Recombinant Protein Infusion
Weight (g)
Time (Weeks)
VAT (g)
SAT (g)
Liver (g)
Plasma Bile Acids (pmol)
Plasma ALT (U/L)
Plasma AST (U/L)
Plasma TG (mg/dl)
Plasma CH (mg/dl)
Plasma GLP-1 (pg/ml)
Plasma PYY (pg/ml)
Caloric Intake (Kcal)
SHAM-Op
Sham-Op
SHAM
DJB
DJB+rHSP70
DJB+rGRP78
<0.0001
0.004
0.0009
0.0002
0.007
0.003
0.002
0.02
0.0005
0.0008
0.01
0.0006

Figure S10.

a, Average body weight time course; the dotted line represents the time when surgery was performed and when the osmotic pumps were implanted to infuse recombinant HSP70 or GRP78 or saline solution. **b-d,** Weight of visceral adipose tissue (VAT) (b), subcutaneous adipose tissue (SAT) (c), liver weight (d). **e-k,** Plasma levels of total Bile Acids (e) Alanine aminotransferase (ALT) (f), Aspartate aminotransferase (AST) (g), Triglycerides (TG) (h), Cholesterol (CH) (i), Glucagon-like peptide-1 (GLP-1) (j) and Peptide YY (PYY) (k) in rats after sham-operation (Sham-Op) or duodenal jejunal bypass (DJB) and infused with recombinant HSP70 or GRP78 or saline solution. **l,** Time course of caloric intake; the dotted line represents the time when surgery was performed and when the osmotic pumps were implanted to infuse recombinant HSP70 or GRP78 or saline solution. Data are mean±SEM of n=10 biologically independent animals. Statistical significances were calculated by Kruskal–Wallis test with Dunnett's correction for multiple comparisons. Source data are provided as a Source Data file.

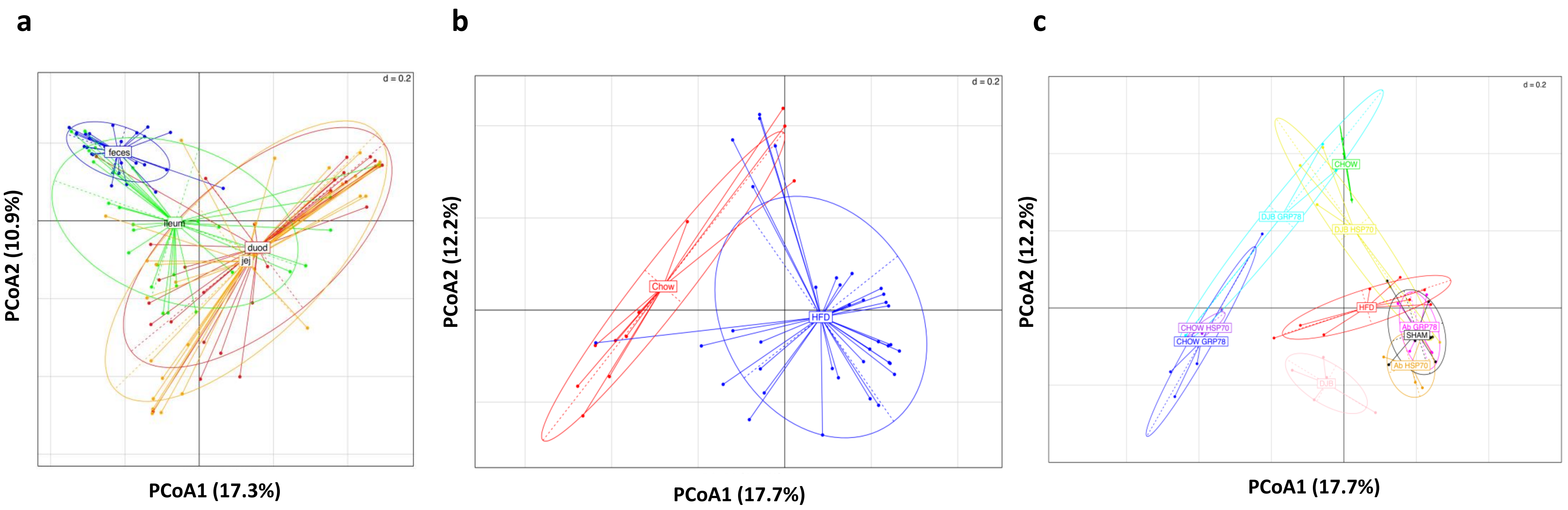

a
b
c
d = 0.2
feces
ileum
duod
jej
PCoA2 (10.9%)
PCoA1 (17.3%)
Chow
HFD
PCoA2 (12.2%)
PCoA1 (17.7%)
CHOW
DJB GRP78
DJB HSP70
HFD
CHOW HSP70
CHOW GRP78
Ab GRP78
SHAM
Ab HSP70
DJB
PCoA2 (12.2%)
PCoA1 (17.7%)

Figure S11.

a, Principal component analysis (PCoA) on the Bray-Curtis dissimilarity index calculated for gut microbiota profiles of amplicon sequence variants (ASVs). Small intestinal (duod, duodenum, n=20; jej, jejunum, n=17; ile, ileum, n=27) and fecal samples (n=48) were added in this analysis. **b,** PCoA on the Bray-Curtis dissimilarity index calculated for fecal microbiota profiles of amplicon sequence variants (ASVs) colored by diet (Chow, n=11; HFD, n=37) and **c,** colored by group (Chow, n=3; Chow+rHSP70, n=4; Chow+rGRP78, n=4; HFD, n=8; HFD+AbHSP70, n=4; HFD+AbGRP78, n=5; Sham, n=7; DJB, n=5; DJB+rHSP70, n=5; DJB+rGRP78, n=3). The numbers in brackets next to the axes indicate the amount of compositional variation explained by each PCo.

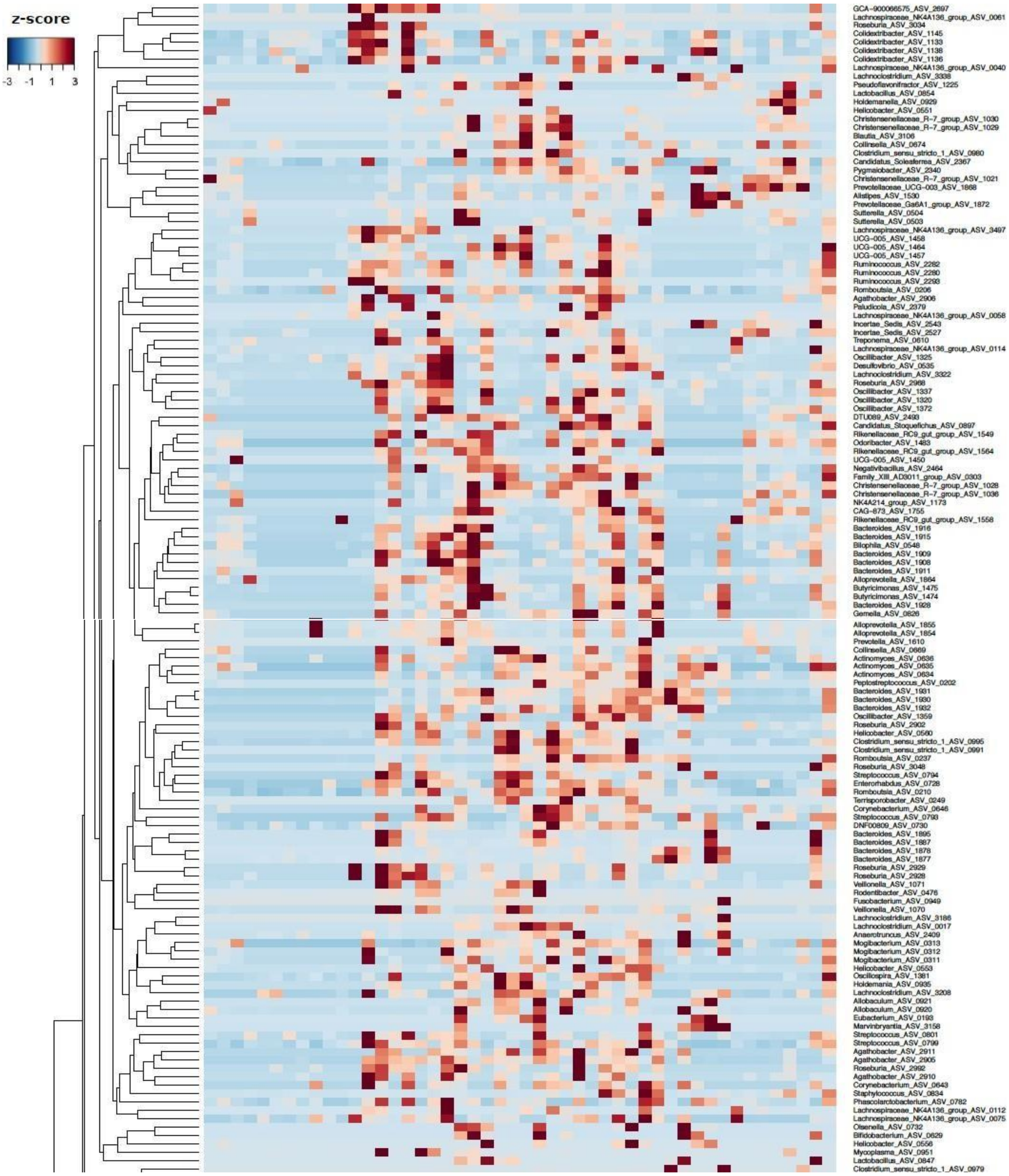
z-score
-3
-1
1
3
GCA-900066575_ASV_2697
Lachnospiraceae_NK4A136_group_ASV_0061
Roseburia_ASV_3034
Colidextribacter_ASV_1145
Colidextribacter_ASV_1133
Colidextribacter_ASV_1138
Colidextribacter_ASV_1136
Lachnospiraceae_NK4A136_group_ASV_0040
Lachnoclostridium_ASV_3338
Pseudoflavonifractor_ASV_1225
Lactobacillus_ASV_0854
Holdemanella_ASV_0929
Helicobacter_ASV_0551
Christensenellaceae_R-7_group_ASV_1030
Christensenellaceae_R-7_group_ASV_1029
Blautia_ASV_3106
Collinsella_ASV_0674
Clostridium_sensu_stricto_1_ASV_0980
Candidatus_Soleaferrea_ASV_2367
Pygmaiobacter_ASV_2340
Christensenellaceae_R-7_group_ASV_1021
Prevotellaceae_UCG-003_ASV_1868
Alistipes_ASV_1530
Prevotellaceae_Ga6A1_group_ASV_1872
Sutterella_ASV_0504
Sutterella_ASV_0503
Lachnospiraceae_NK4A136_group_ASV_3497
UCG-005_ASV_1458
UCG-005_ASV_1464
UCG-005_ASV_1457
Ruminococcus_ASV_2282
Ruminococcus_ASV_2280
Ruminococcus_ASV_2293
Romboutsia_ASV_0206
Agathobacter_ASV_2906
Paludicola_ASV_2379
Lachnospiraceae_NK4A136_group_ASV_0058
Incertae_Sedis_ASV_2543
Incertae_Sedis_ASV_2527
Treponema_ASV_0610
Lachnospiraceae_NK4A136_group_ASV_0114
Oscillibacter_ASV_1325
Desulfovibrio_ASV_0535
Lachnoclostridium_ASV_3322
Roseburia_ASV_2968
Oscillibacter_ASV_1337
Oscillibacter_ASV_1320
Oscillibacter_ASV_1372
DTU089_ASV_2493
Candidatus_Stoquefichus_ASV_0897
Rikenellaceae_RC9_gut_group_ASV_1549
Odoribacter_ASV_1483
Rikenellaceae_RC9_gut_group_ASV_1564
UCG-005_ASV_1450
Negativibacillus_ASV_2464
Family_XIII_AD3011_group_ASV_0303
Christensenellaceae_R-7_group_ASV_1028
Christensenellaceae_R-7_group_ASV_1036
NK4A214_group_ASV_1173
CAG-873_ASV_1755
Rikenellaceae_RC9_gut_group_ASV_1558
Bacteroides_ASV_1916
Bacteroides_ASV_1915
Bilophila_ASV_0548
Bacteroides_ASV_1909
Bacteroides_ASV_1908
Bacteroides_ASV_1911
Alloprevotella_ASV_1864
Butyricimonas_ASV_1475
Butyricimonas_ASV_1474
Bacteroides_ASV_1928
Gemella_ASV_0826
Alloprevotella_ASV_1855
Alloprevotella_ASV_1854
Prevotella_ASV_1610
Collinsella_ASV_0669
Actinomyces_ASV_0636
Actinomyces_ASV_0635
Actinomyces_ASV_0634
Peptostreptococcus_ASV_0202
Bacteroides_ASV_1931
Bacteroides_ASV_1930
Bacteroides_ASV_1932
Oscillibacter_ASV_1359
Roseburia_ASV_2902
Helicobacter_ASV_0560
Clostridium_sensu_stricto_1_ASV_0995
Clostridium_sensu_stricto_1_ASV_0991
Romboutsia_ASV_0237
Roseburia_ASV_3048
Streptococcus_ASV_0794
Enterorhabdus_ASV_0728
Romboutsia_ASV_0210
Terrisporobacter_ASV_0249
Corynebacterium_ASV_0646
Streptococcus_ASV_0793
DNF00809_ASV_0730
Bacteroides_ASV_1895
Bacteroides_ASV_1887
Bacteroides_ASV_1878
Bacteroides_ASV_1877
Roseburia_ASV_2929
Roseburia_ASV_2928
Veillonella_ASV_1071
Rodentibacter_ASV_0476
Fusobacterium_ASV_0949
Veillonella_ASV_1070
Lachnoclostridium_ASV_3186
Lachnoclostridium_ASV_0017
Anaerotruncus_ASV_2409
Mogibacterium_ASV_0313
Mogibacterium_ASV_0312
Mogibacterium_ASV_0311
Helicobacter_ASV_0553
Oscillospira_ASV_1381
Holdemania_ASV_0935
Lachnoclostridium_ASV_3208
Allobaculum_ASV_0921
Allobaculum_ASV_0920
Eubacterium_ASV_0193
Marvinbryantia_ASV_3158
Streptococcus_ASV_0801
Streptococcus_ASV_0799
Agathobacter_ASV_2911
Agathobacter_ASV_2905
Roseburia_ASV_2992
Agathobacter_ASV_2910
Corynebacterium_ASV_0643
Staphylococcus_ASV_0834
Phascolarctobacterium_ASV_0782
Lachnospiraceae_NK4A136_group_ASV_0112
Lachnospiraceae_NK4A136_group_ASV_0075
Olsenella_ASV_0732
Bifidobacterium_ASV_0629
Helicobacter_ASV_0556
Mycoplasma_ASV_0951
Lactobacillus_ASV_0847
Clostridium_sensu_stricto_1_ASV_0979

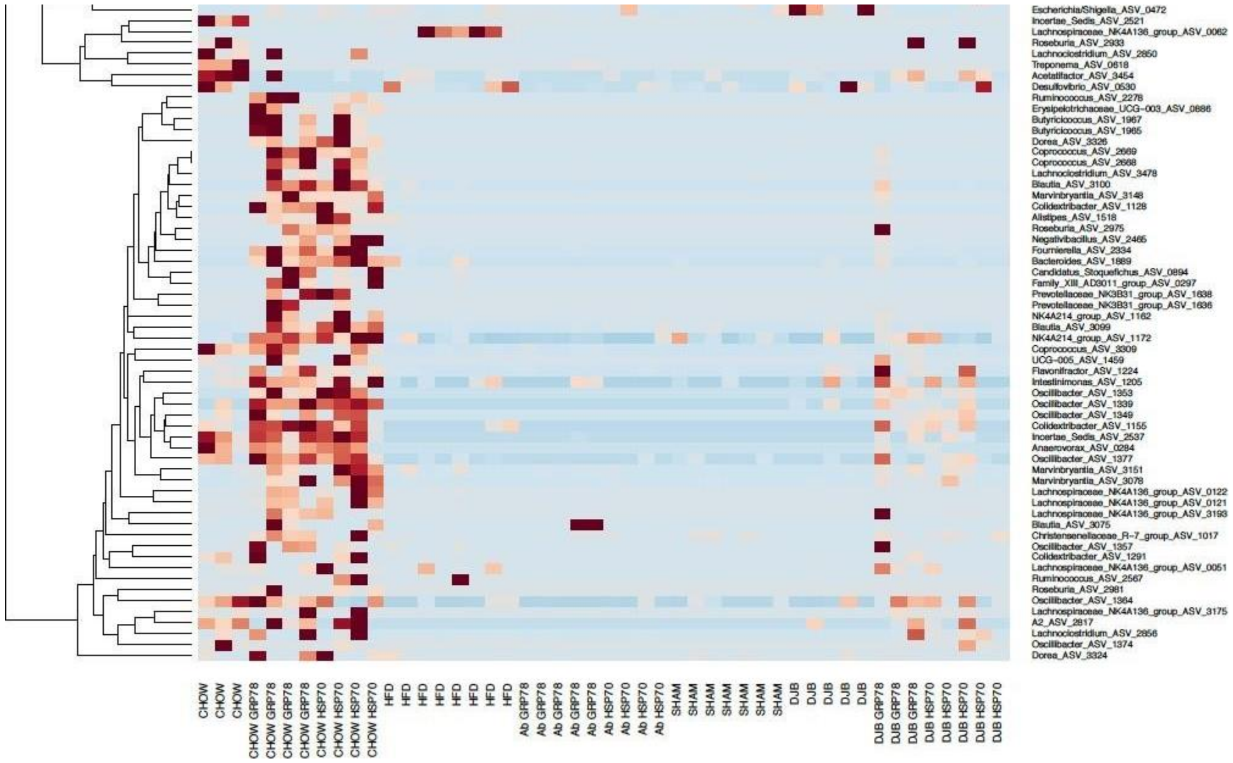
Escherichia/Shigella_ASV_0472
Incertae_Sedis_ASV_2521
Lachnospiraceae_NK4A136_group_ASV_0062
Roseburia_ASV_2933
Lachnoclostridium_ASV_2850
Treponema_ASV_0618
Acetatifactor_ASV_3454
Desulfovibrio_ASV_0530
Ruminococcus_ASV_2278
Erysipelotrichaceae_UCG-003_ASV_0686
Butyricicoccus_ASV_1967
Butyricicoccus_ASV_1965
Dorea_ASV_3326
Coprococcus_ASV_2669
Coprococcus_ASV_2668
Lachnoclostridium_ASV_3478
Blautia_ASV_3100
Marvinbryantia_ASV_3148
Colidextribacter_ASV_1128
Alistipes_ASV_1518
Roseburia_ASV_2975
Negativibacillus_ASV_2465
Fournierella_ASV_2334
Bacteroides_ASV_1889
Candidatus_Stoquefichus_ASV_0894
Family_XIII_AD3011_group_ASV_0297
Prevotellaceae_NK3B31_group_ASV_1638
Prevotellaceae_NK3B31_group_ASV_1636
NK4A214_group_ASV_1162
Blautia_ASV_3099
NK4A214_group_ASV_1172
Coprococcus_ASV_3309
UCG-005_ASV_1459
Flavonifractor_ASV_1224
Intestinimonas_ASV_1205
Oscillibacter_ASV_1353
Oscillibacter_ASV_1339
Oscillibacter_ASV_1349
Colidextribacter_ASV_1155
Incertae_Sedis_ASV_2537
Anaerovorax_ASV_0284
Oscillibacter_ASV_1377
Marvinbryantia_ASV_3151
Marvinbryantia_ASV_3078
Lachnospiraceae_NK4A136_group_ASV_0122
Lachnospiraceae_NK4A136_group_ASV_0121
Lachnospiraceae_NK4A136_group_ASV_3193
Blautia_ASV_3075
Christensenellaceae_R-7_group_ASV_1017
Oscillibacter_ASV_1357
Colidextribacter_ASV_1291
Lachnospiraceae_NK4A136_group_ASV_0051
Ruminococcus_ASV_2567
Roseburia_ASV_2981
Oscillibacter_ASV_1364
Lachnospiraceae_NK4A136_group_ASV_3175
A2_ASV_2817
Lachnoclostridium_ASV_2856
Oscillibacter_ASV_1374
Dorea_ASV_3324
CHOW
CHOW
CHOW
CHOW GRP78
CHOW GRP78
CHOW GRP78
CHOW GRP78
CHOW HSP70
CHOW HSP70
CHOW HSP70
CHOW HSP70
HFD
HFD
HFD
HFD
HFD
HFD
HFD
HFD
Ab GRP78
Ab GRP78
Ab GRP78
Ab GRP78
Ab GRP78
Ab HSP70
Ab HSP70
Ab HSP70
Ab HSP70
SHAM
SHAM
SHAM
SHAM
SHAM
SHAM
SHAM
DJB
DJB
DJB
DJB
DJB
DJB GRP78
DJB GRP78
DJB GRP78
DJB HSP70
DJB HSP70
DJB HSP70
DJB HSP70
DJB HSP70

Figure S12.

Heat map of prevalent differentially abundant amplicon sequence variants (ASVs) (FDR-adjusted p value < 0.05) present in at least 10% of the fecal samples and with taxonomic affiliation at least at genus level. The red-blue color scale indicates z-score normalized relative abundance. CD, n=3; CD+rHSP70, n=4; CD+rGRP78, n=4; HFD, n=8; HFD+AbHSP70, n=4; HFD+AbGRP78, n=5; Sham, n=7; DJB, n=5; DJB+rHSP70, n=5; DJB+rGRP78, n=3.

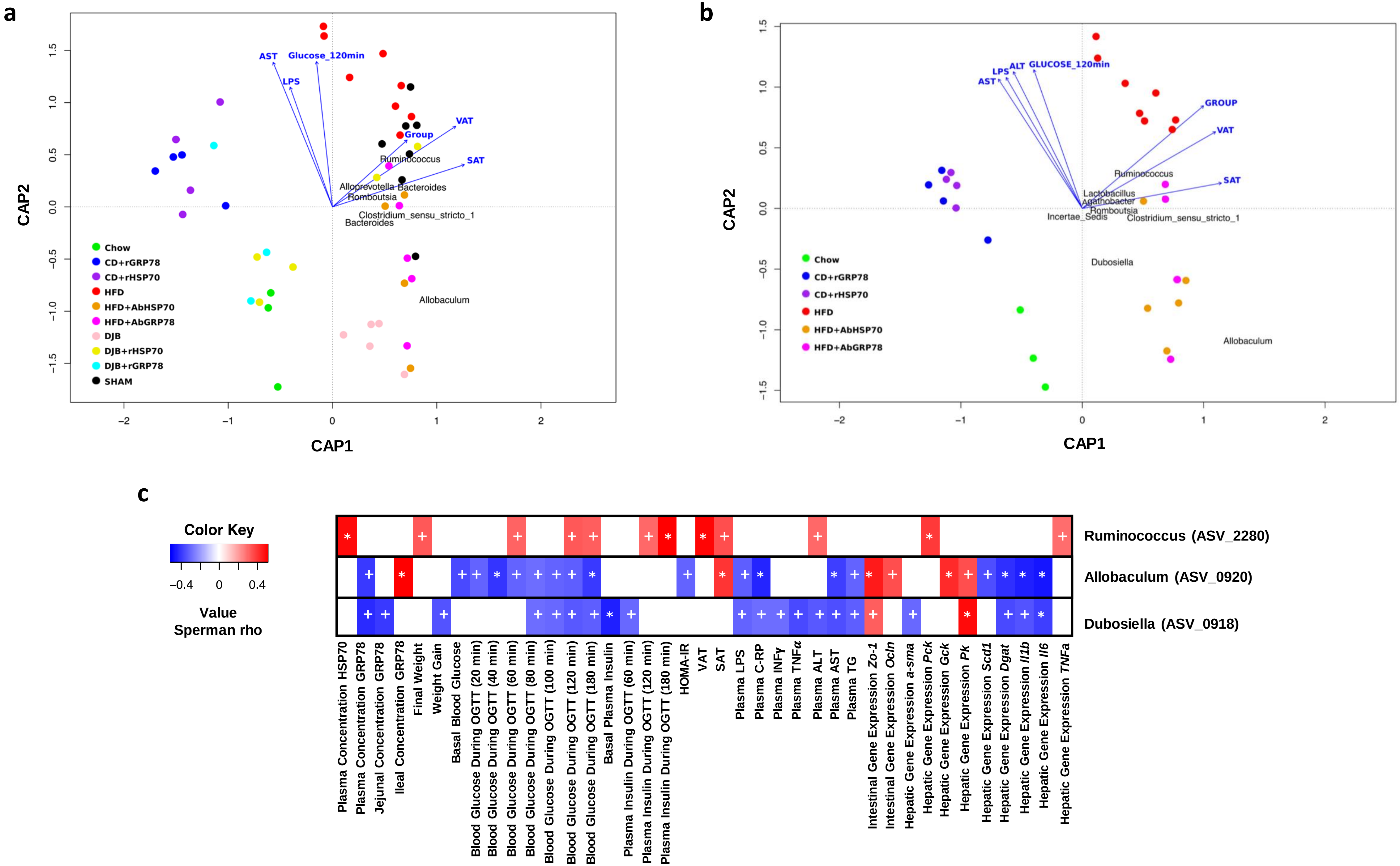

a
b
c
CAP1
CAP2
AST
Glucose_120min
LPS
VAT
Group
SAT
Ruminococcus
Alloprevotella
Bacteroides
Romboutsia
Clostridium_sensu_stricto_1
Allobaculum
Chow
CD+rGRP78
CD+rHSP70
HFD
HFD+AbHSP70
HFD+AbGRP78
DJB
DJB+rHSP70
DJB+rGRP78
SHAM
ALT
GLUCOSE_120min
GROUP
Lactobacillus
Agathobacter
Incertae_Sedis
Dubosiella
Color Key
Value
Sperman rho
Ruminococcus (ASV_2280)
Allobaculum (ASV_0920)
Dubosiella (ASV_0918)
Plasma Concentration HSP70
Plasma Concentration GRP78
Jejunal Concentration GRP78
Ileal Concentration GRP78
Final Weight
Weight Gain
Basal Blood Glucose
Blood Glucose During OGTT (20 min)
Blood Glucose During OGTT (40 min)
Blood Glucose During OGTT (60 min)
Blood Glucose During OGTT (80 min)
Blood Glucose During OGTT (100 min)
Blood Glucose During OGTT (120 min)
Blood Glucose During OGTT (180 min)
Basal Plasma Insulin
Plasma Insulin During OGTT (60 min)
Plasma Insulin During OGTT (120 min)
Plasma Insulin During OGTT (180 min)
HOMA-IR
VAT
SAT
Plasma LPS
Plasma C-RP
Plasma INFγ
Plasma TNFα
Plasma ALT
Plasma AST
Plasma TG
Intestinal Gene Expression Zo-1
Intestinal Gene Expression Ocln
Hepatic Gene Expression a-sma
Hepatic Gene Expression Pck
Hepatic Gene Expression Gck
Hepatic Gene Expression Pk
Hepatic Gene Expression Scd1
Hepatic Gene Expression Dgat
Hepatic Gene Expression Il1b
Hepatic Gene Expression Il6
Hepatic Gene Expression TNFa

Figure S13.

a, Distance-based redundancy analysis (db-RDA) for Bray-Curtis dissimilarity distances of amplicon sequence variants (ASVs) fecal microbiota profiles, metabolic/inflammatory variables and microbial taxa that best explain the variation of gut microbiota composition in fecal samples (CD, n=3; CD+rHSP70, n=4; CD+rGRP78, n=4; HFD, n=8; HFD+AbHSP70, n=4; HFD+AbGRP78, n=5; Sham, n=7; DJB, n=5; DJB+rHSP70, n=5; DJB+rGRP78, n=3). The statistical model constrained 21.8% of the compositional variation ($p<0.001$). Arrows indicate variables significantly correlated with variation of fecal microbiota composition. AST, aspartate aminotransferase; LPS, lipopolysaccharides; Glucose_120min, blood glucose 120 minutes after oral glucose challenge; VAT, visceral adipose tissue; SAT, subcutaneous adipose tissue. Group is a categorical variable that indicates control and treated samples as: 1=Chow; 2=CD+rGRP78; 3=CD+rHSP70; 4=HFD; 5=HFD+AbGRP78; 6=HFD+AbHSP70; 7=DJB; 8=DJB+rGRP78; 9=DJB+rHSP70; 10=SHAM. **b,** db-RDA for Bray-Curtis dissimilarity distances of ASVs profiles, metabolic/inflammatory variables and microbial taxa that best explain the variation of gut microbiota composition in fecal samples from chow diet CD HFD fed rats treated with recombinant proteins (rHSP70, rGRP78) or blocking antibodies (AbHSP70, AbGRP78) (CD, n=3; CD+rHSP70, n=4; CD+rGRP78, n=4; HFD, n=8; HFD+AbHSP70, n=4; HFD+AbGRP78, n=5). The statistical model constrained 32.5% of the compositional variation (p-value=0.001). Arrows indicate variables significantly correlated with variation of fecal microbiota composition. ALT, alanine aminotransferase; AST, aspartate aminotransferase; LPS, lipopolysaccharides; Glucose_120min, blood glucose 120 minutes after oral glucose challenge; VAT, visceral adipose tissue; SAT, subcutaneous adipose tissue. Group is a categorical variable that indicates control and treated samples as: 1=Chow; 2=CD+rGRP78; 3=CD+rHSP70; 4=HFD+AbGRP78; 5=HFD+AbHSP70; 6=HFD. **c,** Significant Spearman's correlations between abundance of fecal ASVs and metabolic/inflammatory variables. The red-blue color scale indicates the direction and intensity of Spearman's correlations, with red and blue showing positive and negative correlations, respectively. +$p<0.05$; *$p<0.001$ (FDR adjusted).

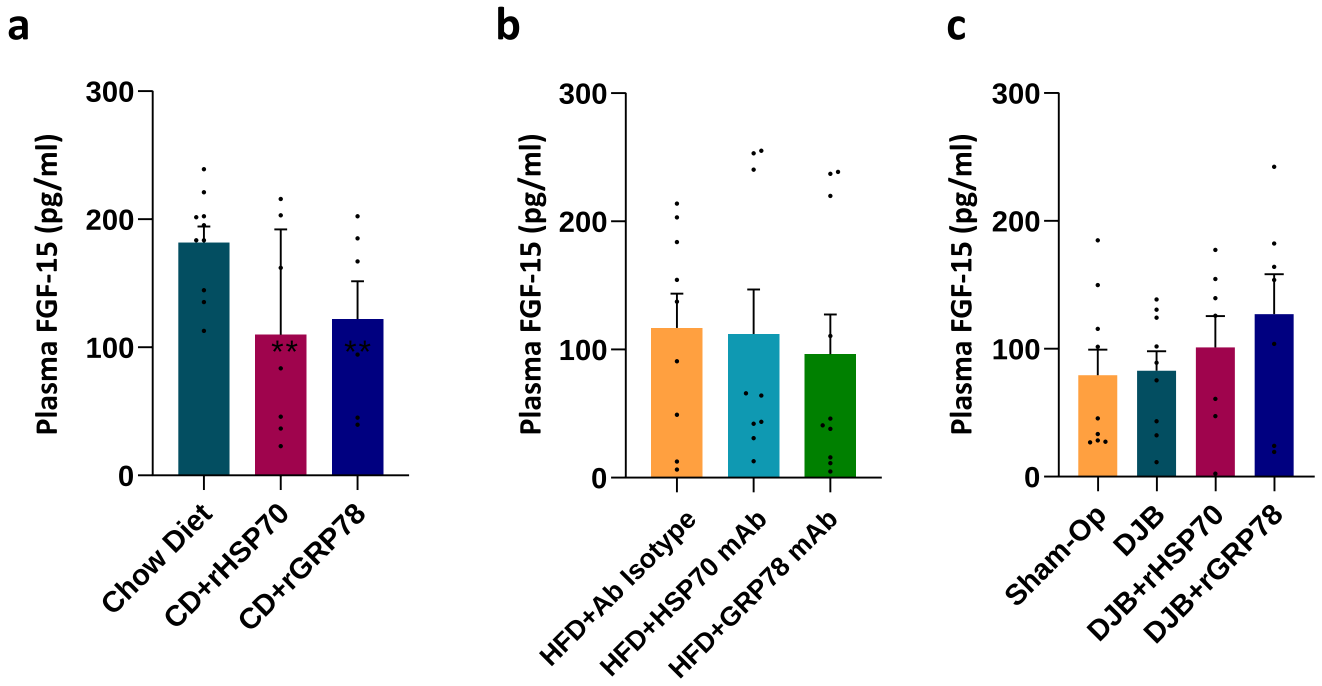
a
b
c
Plasma FGF-15 (pg/ml)
300
200
100
0
**
**
Chow Diet
CD+rHSP70
CD+rGRP78
HFD+Ab Isotype
HFD+HSP70 mAb
HFD+GRP78 mAb
Sham-Op
DJB
DJB+rHSP70
DJB+rGRP78

Figure S14.

a-c, Plasma levels of Fibroblast growth factor 15 (FGF15). Data are mean ±SEM from n=10 biologically independent animals. Statistical significances were calculated by Kruskal–Wallis test with Dunnett's correction for multiple comparisons. Source data are provided as a Source Data file.

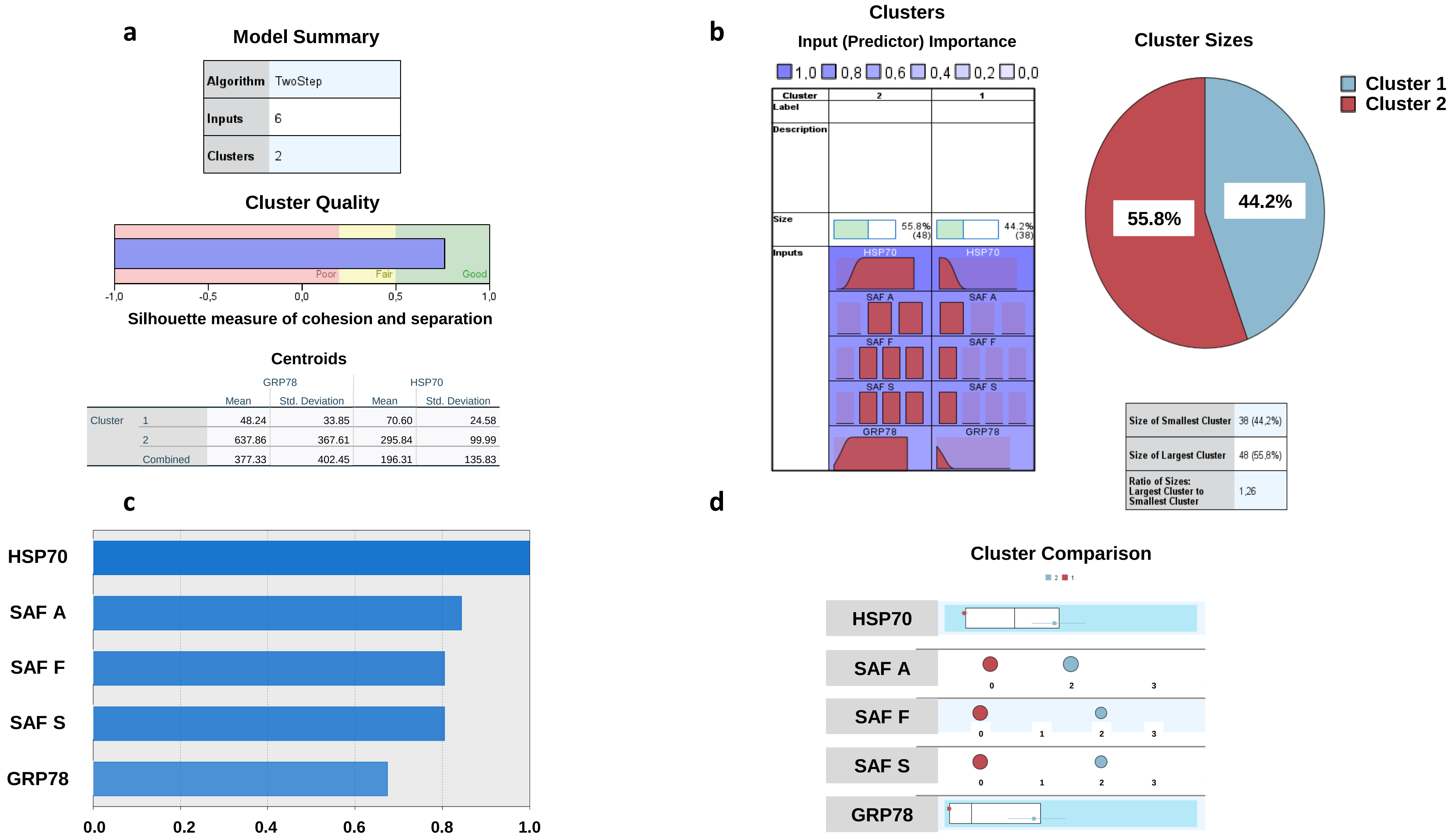

Algorithm	TwoStep
Inputs	6
Clusters	2

		GRP78		HSP70	
		Mean	Std. Deviation	Mean	Std. Deviation
Cluster	1	48.24	33.85	70.60	24.58
	2	637.86	367.61	295.84	99.99
	Combined	377.33	402.45	196.31	135.83

Size of Smallest Cluster	38 (44,2%)
Size of Largest Cluster	48 (55,8%)
Ratio of Sizes: Largest Cluster to Smallest Cluster	1,26

Figure S15.

a, Two-step partitioning cluster analysis with HSP70 and GRP78 as continuous variables and histological liver scores, i.e. SAF-S, SAF-A and SAF-F, as categorical variables. The quality of the cluster is good. The centroids values of the two clusters (subjects with NASH vs. healthy controls) for HSP70 and GRP78 are reported in the table. **b,** The cluster size (47 subjects with NASH and 39 healthy controls) shows that 1 HC was misclassified. The input importance shows that HSP70 and GRP78 are major predictors of NASH presence. **c,** Importance of HSP70, SAF-A, SAF-F, SAF-S and GRP78 as predictors of NASH presence. **d,** Comparison of cluster composition**.**

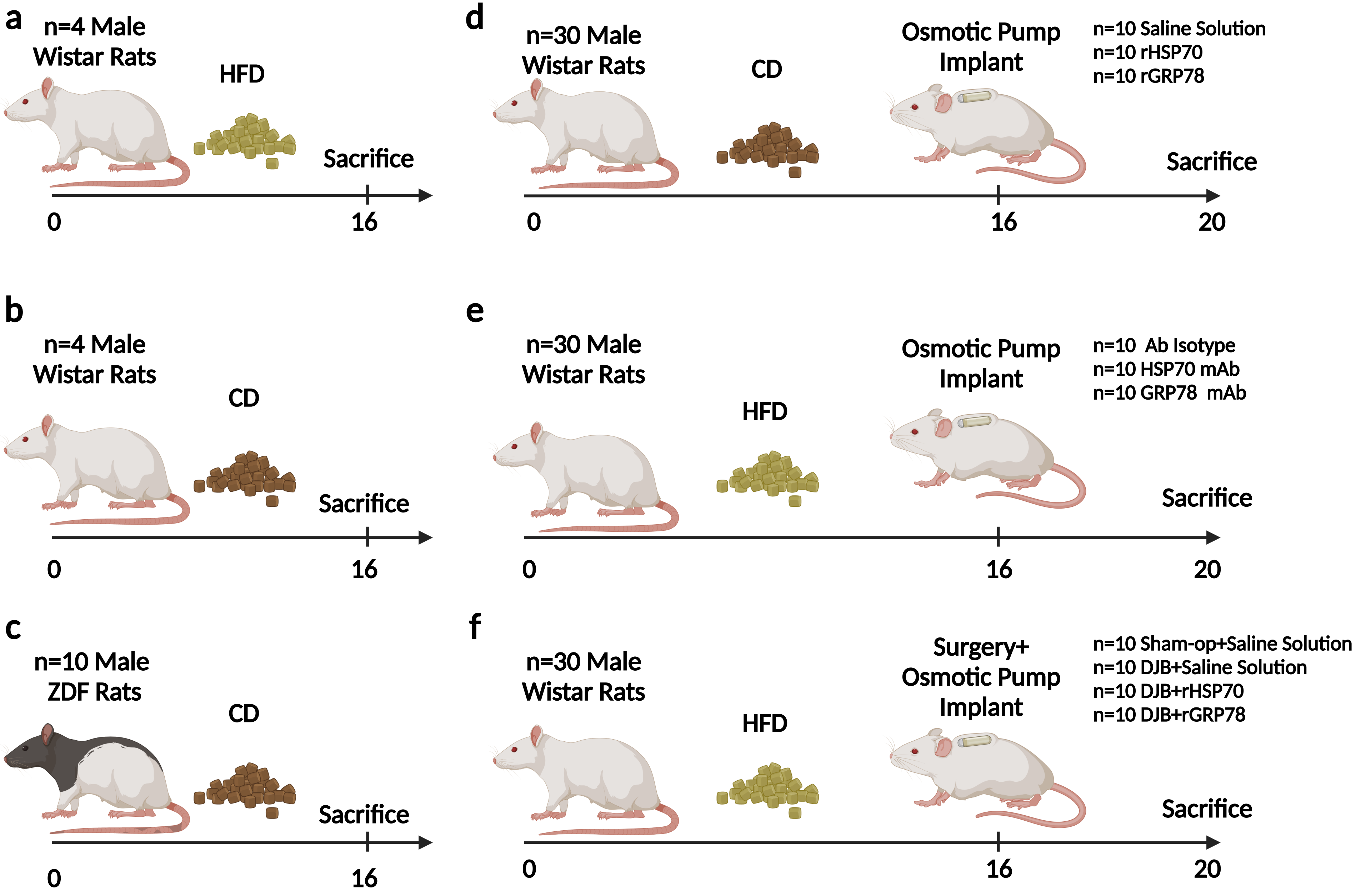
a
n=4 Male
Wistar Rats
HFD
Sacrifice
0
16
b
n=4 Male
Wistar Rats
CD
Sacrifice
0
16
c
n=10 Male
ZDF Rats
CD
Sacrifice
0
16
d
n=30 Male
Wistar Rats
CD
Osmotic Pump
Implant
n=10 Saline Solution
n=10 rHSP70
n=10 rGRP78
Sacrifice
0
16
20
e
n=30 Male
Wistar Rats
HFD
Osmotic Pump
Implant
n=10 Ab Isotype
n=10 HSP70 mAb
n=10 GRP78 mAb
Sacrifice
0
16
20
f
n=30 Male
Wistar Rats
HFD
Surgery+
Osmotic Pump
Implant
n=10 Sham-op+Saline Solution
n=10 DJB+Saline Solution
n=10 DJB+rHSP70
n=10 DJB+rGRP78
Sacrifice
0
16
20

Figure S16.

a-c, Experimental design of the study involving dietary intervention. **d,e,** Experimental design of the study involving dietary intervention and infusion of recombinant protein or blocking monoclonal antibodies. **f,** Experimental design of the study including metabolic surgery and infusion of recombinant protein. Created with BioRender.com.

a

b

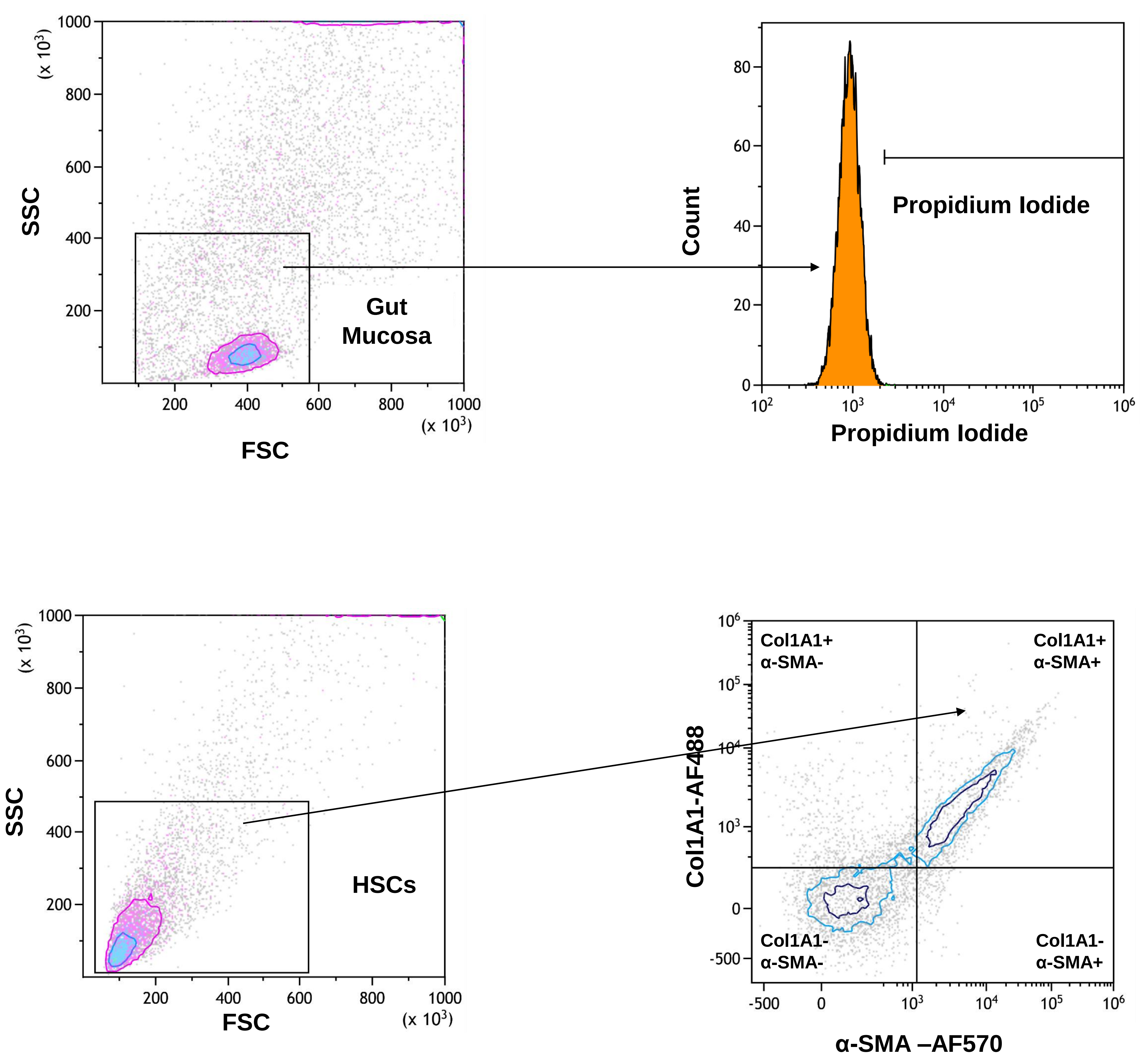

SSC
(x 10³)
FSC
(x 10³)
Gut Mucosa
Count
Propidium Iodide
Propidium Iodide
HSCs
Col1A1+ α-SMA-
Col1A1+ α-SMA+
Col1A1- α-SMA-
Col1A1- α-SMA+
Col1A1-AF488
α-SMA –AF570

Figure S17.

a, Gating strategy of gut mucosa cell death using propidium iodide staining. **b,** Gating strategies of rat hepatic stellate cells (HSCs) stained with alpha-smooth muscle Actin (α-SMA) and Collagen type I alpha 1 (COL1A1).

SUPPLEMTENTARY TABLES

Table S1. Recombinant and native HSP70 and GRP78 kinetics in rats

	**A (ng/ml·min^{-1})**	**K1 (l·min^{-1})**	**K2 (l·min^{-1})**	**Tm (ng·min^{-1})**	**Km (ng/ml)**	**EV (min)**
**rHSP70**	8.37±1.50	0.034±0.006	0.018±0.009	0.031±0.004	7.74±0.73	64.33±6.15
**Native HSP70**	7.95±1.69	0.031±0.011	0.010±0.002	0.030±0.004	8.94±0.12	68.33±12.78
**rGRP78**	4.4±1.43	0.009±0.003	0.010±0.003	0.017±0.005	12.94±5.20	62.17±18.00
**Native GRP78**	2.86±0.68	0.017±0.008	0.009±0.003	0.029±0.009	9.72±1.06	60.50±11.00

Data are expressed as mean ± SEM.

A (ng/ml·min^{-1}) is the maximal inflow rate of the protein; K1 and K2 are the rates (1/min^{-1}) of inflow increase and decrease; Tm (ng/min) and Km (ng/mL) are the parameters of the Michaelis-Menten function.

Table S2. Insulin sensitivity and insulin secretion, measured by the oral glucose minimal model

	**SG×10^2 (min^{-1})**	**p×10^2 (min^{-1})**	**SIx10^4 (pM^{-1} min^{-1})**	**Volume (mL)**	**Φx10^9 (min^{-1})**	**AUCISR (nmoles)**
**Chow Diet**	4.09±0.12	0.15±0.005	0.82±0.04	72.22±0.98	2.99±0.49	1.35±0.15
**CD+rHSP70**	4.060.12	0.24±0.02	0.14±0.02 **P<0.0001**	70.86±0.98	22.33±2.83 **P<0.0001**	22.65±2.68 **P<0.0001**
**CD+rGRP78**	3.80±0.13	0.17±0.02	0.16±0.02 **P<0.0001**	81.14±2.18 **P=0.049**	22.81±4.38 **P<0.0001**	20.94±2.46 **P<0.0001**
**HFD+ Isotype**	3.86±0.12	0.17±0.03	0.15±0.03	96.33±1.42	22.35±2.63	38.41±3.64
**HFD+AbHSP70**	3.91±0.22	0.17±0.04	0.76±0.02 **P<0.0001**	69.20±0.44 **P<0.0001**	3.92±0.69 **P<0.0001**	7.83±1.04 **P<0.0001**
**HFD+AbGRP78**	3.96±0.15	0.18±0.03	0.93±0.04 **P<0.0001**	68.60±0.45 **P<0.0001**	2.84±0.57 **P<0.0001**	7.37±0.97 **P<0.0001**
**Sham-Op**	4.09±0.14	0.33±0.07	0.13±0.005	106.80±1.50	24.862±2.30	40.14±4.73
**DJB**	4.09±0.13	0.16±0.02	0.50±0.04 **P<0.0001**	72.41±2.82 **P<0.0001**	2.99±0.48 **P<0.0001**	1.68±0.40 **P<0.0001**
**DJB+rHSP70**	4.35±0.16	0.15±0.02	0.10±0.01 **P<0.0001**	81.65±1.55 **P=0.0027**	22.85±2.62 **P<0.0001**	20.82±1.23 **P<0.0001**
**DJB+rGRP78**	4.09±0.14	0.16±0.01	0.13±0.005 **P<0.0001**	80.14±0.76 **P=0.0021**	20.34±2.30 **P<0.0001**	20.94±2.46 **P<0.0001**

Data are expressed as mean ± SEM.

SG, glucose effectiveness; p, minimal model parameter; SI, insulin sensitivity; Volume, glucose volume of distribution; Φ, total β-cell glucose sensitivity; AUCISR, incremental area under the curve of the insulin secretion rate over basal; CD, Chow Diet; HFD, High Fat Diet; Sham-op, Sham-operation; DJB, Duodenal-Jejunal Bypass. HSP70, Heat Shock Protein 70; GRP78, Glucose-related Protein 78.

Table S3. Anthropometric and metabolic data of people with NASH and healthy controls.

	People With NASH (n=47)	Healthy Controls (n=39)	pValue
**Age**	49.85±1.33	41.97±1.38	NS
**Weight**	123.5±3.21	63.95±1.75	<0.0001
**BMI**	43.65±0.89	21.62±0.26	<0.0001
**Plasma Glucose (mg/dl)**	105.2±3.96	80.16±1.29	<0.0001
**Plasma Insulin (mIU/l)**	25.08±3.07	8.74±0.30	<0.0001
**HOMA-IR**	6.49±0.85	1.75±0.080	<0.0001
**Cholesterol (mg/dl)**	192.1±4.27	173.2±2.70	0.0007
**HDL (mg/dl)**	54.64±3.59	67.26±1.77	0.0044
***LDL (equation)**	107.9±5.87	99.08±2.14	NS
**Triglycerides(mg/dl)**	145.3±8.98	85.16±3.87	<0.0001
**ALT (IU/l)**	24.89±1.25	16.45±0.86	<0.0001
**AST (IU/l)**	33.70±2.49	17.03±0.99	<0.0001
**HbA1c (mmol/mol)**	43.09±1.27	25.34±0.42	<0.0001
**NAFLD Activity Score (NAS)**	3.88±0.11	0.00±0.00	<0.0001
**SAF S**	1.73±0.093	0.00±0.00	<0.0001
**SAF A**	2.17±0.054	0.00±0.00	<0.0001
**SAF F**	1.69±0.095	0.00±0.00	<0.0001
**Metavir**	1.04±0.073	0.00±0.00	<0.0001
**†NAFLD Fibrosis Score (Formula)**	0.029±0.13	-2.08±0.17	<0.0001
**‡AST to Platelet Ratio Index (Formula)**	0.26±0.022	0.19±0.011	0.015
**Plasma HSP70 (ng/ml)**	282.90±11.74	75.87±36.20	<0.0001
**Plasma GRP78 (ng/ml)**	631.60±80.89	61.06±11.24	<0.0001

Data are expressed as mean±SE.

*LDL-cholesterol (mg/dl) = Total Cholesterol (mg/dl) − HDL-cholesterol (mg/dl) – Triglycerides (mg/dl)/5.

†NAFLD fibrosis score = −1.675 + 0.037 – age (years) + 0.094 – BMI (kg/m^2) + 1.13 × IFG/diabetes (yes = 1, no = 0) + 0.99 × AST/ALT ratio – 0.013 × platelet count (×10^9/l) – 0.66 × albumin (g/dl).

‡AST to Platelet Ratio Index =[(AST (UI/L)/upper limit of the normal AST range) X 100]/Platelet Count (x10^9/l).

Table S4. Anthropometric and metabolic data of people before and after Vertical Sleeve Gastrectomy (VSG) or Low-Fat Diet (LFD).

	**Before VSG (n=10)**	**After VSG (n=10)**	**pValue**	**Before LFD (n=10)**	**After LFD (n=10)**	**pValue**
**Age (years)**	49.60±2.47	50.60±2.47	NS	54.80±3.35	55.80±3.35	NS
**Weight (kg)**	128.10±6.31	85.23±4.38	<0.0001	117.10±7.13	101.7±7.58	0.0003
**BMI (kg/m^2)**	44.58±1.33	29.70±0.90	<0.0001	39.48±2.07	35.27±2.28	0.0001
**Plasma Glucose (mg/dl)**	107.60±9.48	77.30±2.97	0.013	106.0±6.34	89.70±4.90	NS
**Plasma Insulin (mIU/l)**	33.84±8.28	7.22±1.15	0.008	23.56±3.51	7.02±0.64	<0.0001
**HOMA-IR**	9.91±3.18	1.39±0.22	0.024	5.99±0.80	1.55±0.15	<0.0001
**Cholesterol (mg/dl)**	186.30±9.26	15.80±8.74	0.015	191.70±7.72	177.90±13.96	NS
**HDL (mg/dl)**	43.30±4.28	52.20±3.54	0.0492	41.80±2.19	48.00±3.04	NS
***LDL (equation)**	114.30±8.14	90.58±6.36	0.0137	116.20±6.35	99.40±32.77	NS
**Triglycerides(mg/dl)**	144.4±17.12	69.30±8.08	0.0027	167.80±23.78	60.63±20.21	NS
**ALT (IU/l)**	33.70±8.17	14.40±1.43	0.031	39.80±9.19	21.20±3.35	NS
**AST (IU/l)**	23.30±3.66	17.30±0.99	NS	28.40±4.12	20.00±1.76	NS
**HbA1c (mmol/mol)**	34.16±4.87	34.90±0.62	NS	42.89±3.58	37.38±2.70	NS
**Serum HSP70 (ng/ml)**	347.00±16.66	212.60±5.90	<0.0001	298.50±17.86	70.74±5.57	<0.0001
**Serum GRP78 (ng/ml)**	822.20±81.57	423.8±27.15	0.0004	749.50±99.04	276.90±41.02	0.0036

Data are expressed as mean±SE.

*LDL-cholesterol (mg/dl) = Total Cholesterol (mg/dl) − HDL-cholesterol (mg/dl) – Triglycerides (mg/dl)/5.

Table S5. HSP70 and GRP78 kinetics after a mixed meal

	A (ng/ml·min^{-1})	K1 (l·min^{-1})	K2 (l·min^{-1})	Tm (ng·min^{-1})	Km (ng/ml)	EV (min)
**HSP70 (Healthy Participant)**	110.80±27.78	0.031±0.009	0.020±0.004	0.068±0.022	47.62±11.40	49.30±16.49
**HSP70 (Participants With Obesity)**	298.40±34.89	0.053±0.006	0.043±0.007	0.094±0.049	14.00±2.45	32.20±7.85
**GRP78 (Healthy Participants)**	94.40±10.36	0.036±0.005	0.035±0.004	0.039±0.002	10.60±0.24	22.80±4.90
**GRP78 (Participants With Obesity)**	370.00±46.58	0.034±0.004	0.04±0.013	0.057±0.089	60.70±4.38	20.40±2.66

Data are expressed as mean ± SEM.

A (ng/ml·min^{-1}) is the maximal inflow rate of the protein; K1 and K2 are the rates (1/min) of inflow increase and decrease; Tm (ng/min) and Km (ng/mL) are the parameters of the Michaelis-Menten function.

Table S6. Primer sequences used in qRT-PCR

Gene	Primer sequence (5′to 3′)
Phosphoenolpyruvate carboxykinase (Pck)	ATGACAACTGCTGGTTGGCT
	CCACCACGTAGGGTGAATCC
Glucose 6-phosphatase (G6Pc)	ACAGGTCCAGGAAGTCCATCT
	GCATGCCACCAATTACTCCAAG
Glucokinase (Gck)	AGTTGTTGACTCTGGGCACC
	TTCATGTGCCCGTTGTGAGT
Pyruvate kinase (Pk)	CTTCCCCTTGCTCTACCGTG
	ACCACGGAGCTTTCCACTTTC
Interleukin 1β (IL1β)	TCCAGTCAGGCTTCCTTGTG
	AGGTCATTCTCCTCACTGTCG
Interleukin 6 (IL6)	GCCCACCAGGAACGAAAGTC
	TGGCTGGAAGTCTCTTGCGG
Tumor Necrosis Factor α (TNFα)	CGTCGTAGCAAACCACCAAG
	TGGACCCAGAGCCACAATTC
Alpha-smooth muscle actin (Acta)	TTCCTTCGTGACTACTGCTGAG
	GAACTGAAGGCGCTGATCCA
Collagen type I α1 (Col1a1)	GTACATCAGCCCAAACCCCA
	TCGCTTCCATACTCGAACTGG
Transforming growth factor β1 (Tgfb1)	GCTGAACCAAGGAGACGGAA
	CCACGTAGTAGACGATGGGC
Acetyl-CoA Carboxylase (Acc1)	ATTGGGGCTTACCTTGTCCG
	CCACGGTTCGGGTTTCTACA
Fatty acid synthase (Fasn)	GAATCCGCACAGGCTACCAA
	CTGGGCTTCACCATCACCAT
Stearoyl-CoA desaturase 1 (Scd1)	CCACAACTACCATCACGCCT
	CAGGAACTCAGAAGCCCAGAA
Diglyceride acyltransferase (Dgat)	AGCAGGAGTAGGCCCCATAG
	ATTGGGGCTTACCTTGTCCG
Occludin (Ocln)	AGCAACGCGGCTAGGG
	GCAGACACATTTTTAACCCACTC
Haptoglobin (Zon1)	ATGCCACAGACATTGAAGATGAC
	CTCGGGGTGGAGAACTACCT
β2-microglobulin	GACCGTGATCTTTCTGGTGCT
	ACACTTGAATTTGGGGAGTTTTCTG

Primer sequences used for gene expression analysis by qRT-PCR.

Table S7. Quantitative Real-Time PCR

Gene	Chow Diet	CD+rHSP70	CD+rGRP78	pValue
**PEPCK**	5.61±1.86	20.78±2.84	21.07±3.07	†=0.041; ‡=0.014
**G6Pc**	2.69±0.51	21.51±7.14	20.66±4.22	†=0.033; ‡=0.018
**GCK**	33.89±10.19	3.42±0.88	3.45±0.84	†=0.027; ‡=0.022
**PK**	21.46±3.72	3.26±0.62	3.42±0.60	†=0.014; ‡=0.014
**IL1β**	1.81±0.17	13.58±1.62	11.60±3.07	†=0.011; ‡=0.048
**IL6**	20.94±5.95	410.40±187.3	458.10±139.80	†=0.022; ‡=0.027
**TNFα**	2.46±0.41	11.02±4.76	9.73±3.07	†=0.033; ‡=0.018
**α-SMA**	1.35±0.12	5.44±0.82	5.07±0.55	†=0.014; ‡=0.040
**Col1a1**	5.83±1.30	25.60±6.10	19.15±2.48	†=0.014; ‡=0.040
**ACC1**	45.93±14.49	342.70±147.60	402.20±120.10	†=0.040; ‡=0.014
**FASN**	2.89±0.65	12.88±2.06	9.72±1.18	†=0.012; ‡=0.047
**DGAT**	1.42±0.17	38.39±19.09	32.06±10.31	†=0.027; ‡=0.022
**SCD1**	47.77±28.38	2100±1008	2185±1120	†=0.014; ‡=0.040
**OCLN**	0.79±0.02	0.11±0.03	0.15±0.04	†=0.012; ‡=0.046
**ZON1**	0.62±0.07	0.12±0.04	0.11±0.03	†=0.017; ‡=0.033

†= pValue between Chow Diet and CD+rHSP70; ‡= pValue between Chow Diet and CD+rGRP78

Gene	HFD+ Isotype	HFD+AbHSP70	HFD+AbGRP78	pValue
**PEPCK**	24.65±3.19	5.17±1.01	8.77±1.91	†=0.0005; ‡=0.0005
**G6Pc**	18.64±6.34	2.54±0.46	2.29±0.50	†=0.032; ‡=0.017
**GCK**	4.54±1.12	27.71±7.38	30.36±7.15	†=0.027; ‡=0.022
**PK**	3.88±0.52	15.89±2.59	17.70±2.99	†=0.0062; ‡=0.0034
**IL1β**	10.13±2.30	1.75±0.20	2.89±0.06	†=0.040; ‡=0.012
**IL6**	461.4±125.10	13.94±6.48	10.64±3.67	†=0.033; ‡=0.018
**TNFα**	17.22±5.61	2.93±0.40	3.33±0.36	†=0.022; ‡=0.027
**α-SMA**	5.64±0.52	1.99±0.22	1.53±0.24	†<0.0001; ‡<0.0001
**Col1a1**	37.84±6.04	8.33±1.65	8.18±1.93	†=0.022; ‡=0.027
**ACC1**	278.70±97.76	57.74±12.96	65.14±14.92	†=0.014; ‡=0.040
**FASN**	9.45±0.39	3.68±0.59	4.27±0.93	†=0.0002; ‡=0.0003
**DGAT**	37.00±19.47	1.30±0.09	1.20±0.10	†=0.048; ‡=0.011
**SCD1**	1085±292.40	30.80±3.82	15.77±7.66	†=0.0023; ‡=0.0023
**OCLN**	0.14±0.04	0.85±0.05	0.81±0.06	†=0.022; ‡=0.039
**ZON1**	0.12±0.01	0.53±0.09	0.55±0.10	†=0.006; ‡=0.039

†= pValue between High Fat Diet and HFD+AbHSP70 ; ‡= pValue between High Fat Diet and HFD+AbGRP78

Gene	Sham-Op	DJB	CD+rHSP70	CD+rGRP78	pValue
**PEPCK**	49.31±8.05	12.17±3.51	56.38±12.21	51.81±10.82	†=0.016; ‡=0.023; §=0.031
**G6Pc**	45.77±14.26	3.14±0.69	25.12±4.26	26.99±7.03	†=0.0048; ‡=0.042; §=0.048
**GCK**	4.60±1.48	37.20±10.73	5.11±1.40	4.66±1.24	†=0.048;‡=0.026§=0.008
**PK**	3.38±0.93	17.44±4.78	3.27±0.88	3.70±0.96	†=0.015; ‡=0.015; §=0.048
**IL1β**	6.68±1.36	2.04±0.55	6.56±1.26	6.33±1.13	†=0.016 ‡=0.023; §=0.031
**IL6**	254.10±83.88	12.58±5.83	235.50±71.92	234.40±62.42	†=0.026; ‡=0.014; §=0.023
**TNFα**	10.31±2.70	2.90±0.39	9.83±2.55	8.55±1.36	†=0.016; ‡=0.036; §=0.019
**α-SMA**	22.24±3.06	3.53±0.47	19.50±3.30	16.76±3.58	†=0.0058; ‡=0.036; §=0.048
**Col1a1**	70.06±6.30	32.46±3.46	70.57±4.75	77.90±8.02	†=0.0048; ‡=0.026; §=0.008
**ACC1**	475.40±197.60	55.35±15.13	281.80±75.79	395.60±142.0	†=0.014; ‡=0.042; §=0.019
**FASN**	13.69±3.26	3.25±0.80	14.72±3.40	10.57±1.10	†=0.023; ‡=0.012; §=0.042
**DGAT**	25.77±7.78	1.66±0.21	19.71±5.93	20.33±4.59	†=0.023; ‡=0.049; §=0.036
**SCD1**	4652±2720	49.25±7.65	4450±2710	4360±1339	†=0.036; ‡=0.016; §=0.019
**OCLN**	0.11±0.007	0.84±0.02	0.15±0.01	0.015±0.009	†=0.0004;‡=0.032;§=0.032
**ZON1**	45.77±14.26	3.14±0.69	25.12±4.26	26.99±7.03	†=0.0005‡=0.032;§=0.032

†= pValue between SHAM and DJB ; ‡= pValue between DJB and DJB+rHSP70; §= pValue between DJB and DJB+rGRP78

Data are mean ± SEM of n=10 rats per group.

Upper Part: gene expression analysis of rats under Chow Diet and Chow Diet combined with continuous infusion of recombinant proteins.

Middle Part: gene expression analysis of rats under High Fat Diet and HFD combined with continuous infusion of blocking antibodies.

Lower Part: gene expression analysis of rats after SHAM, DJB and DJB combined with continuous infusion of recombinant proteins.

Abbreviations: PEPCK1, Phosphoenolpyruvate carboxykinase 1; G6Pase, Glucose 6-phosphatase; GCK, Glucokinase; PK, Pyruvate kinase; IL1β, Interleukin 1β; IL6, Interleukin 6; TNFα, Tumor Necrosis Factor α; α-SMA, Alpha-smooth muscle actin; Col1a1, Collagen type I α1; ACC1, acetyl-CoA carboxylase; FASN, Fatty acid synthase; SCD1, Stearoyl-CoA desaturase 1; DGAT, Diglyceride acyltransferase; OCLN, Occludin; ZON1, Zonulin 1. CD, Chow Diet; HFD, High Fat Diet; Sham-op, Sham-operation; DJB, Duodenal-Jejunal Bypass. HSP70, Heat Shock Protein 70; GRP78, Glucose-related Protein 78.

Table S8. Quantitative Real-Time PCR of in vitro experiments

Gene	Untreated	+rHSP70	TAK242+rHSP70	+rGRP78	TAK242+rGRP78
			**Rat Primary Hepatocytes Cultures**		
**CD36**	2.01±0.25	20.60±6.54	2.20±0.02<br>**P=0.035**	18.13±8.00	1.23±0.13<br>**P=0.035**
**ACC1**	3.56±0.72	84.68±25.44	2.58±0.63<br>**P=0.036**	46.05±20.39	2.67±0.74<br>**P=0.036**
**FASN**	2.43±0.46	65.80±11.08	3.87±0.18<br>**P=0.036**	49.38±12.98	1.91±0.34<br>**P=0.036**
**DGAT**	1.53±0.13	3.78±0.67	1.54±0.27<br>**P=0.036**	3.22±0.39	1.70±0.13<br>**P=0.036**
**SCD1**	75.17±14.82	1230.00±371.8	104.00±3.58<br>**P=0.036**	964.60±294.60	77.67±12.22<br>**P=0.036**
			**Rat Primary Colon epithelial cell Cultures**		
**IL1β**	32.90±13.48	161.50±49.08	26.00±14.06<br>**P=0.029**	131.5±35.20	33.79±17.10<br>**P=0.047**
**IL6**	0.94±0.09	3.24±0.54	1.31±0.12<br>**P=0.029**	2.79±0.32	1.51±0.15<br>**P=0.029**
**TNFα**	2.80±0.47	10.55±0.88	3.84±0.19<br>**P=0.029**	12.37±1.28	3.96±1.32<br>**P=0.029**
**OCLN**	0.11±0.007	0.84±0.02	0.15±0.01	0.015±0.009	131.5±35.20<br>**P=0.029**
**ZON1**	2.71±0.22	10.09±0.04	2.62±0.19<br>**P=0.029**	1.40±0.1	2.67±0.17<br>**P=0.029**

Data are mean ± SEM of n=5 independent experiments.

Upper Part: gene expression analysis of rat primary hepatocytes cultures incubated with or without recombinant proteins (15 ng/ml of HSP70 and 3 ng/ml of GRP78) and in the presence or absence of TAK-242 (200nM)

Lower Part: gene expression analysis of rat primary colon epithelial cell cultures incubated with or without recombinant proteins (15 ng/ml of HSP70 and 3 ng/ml of GRP78) and in the presence or absence of TAK-242 (200nM)

Abbreviations: IL1β, Interleukin 1β; IL6, Interleukin 6; TNFα, Tumor Necrosis Factor α; ACC1, acetyl-CoA carboxylase; FASN, Fatty acid synthase; SCD1, Stearoyl-CoA desaturase 1; DGAT, Diglyceride acyltransferase; OCLN, Occludin; ZON1, Zonulin 1; HSP70, Heat Shock Protein 70; GRP78, Glucose-related Protein 78.
