# Supplementary material for: Upper gut heat shock proteins HSP70 and GRP78 promote insulin resistance, hyperglycemia, and non-alcoholic steatohepatitis
Source: Nat Commun. 2022 Dec 13;13:7715. doi: 10.1038/s41467-022-35310-5 (PMC9748124; doi:10.1038/s41467-022-35310-5)
Supplement: Supplementary file 2 — Reporting Summary [file 41467_2022_35310_MOESM2_ESM.pdf]

nature portfolio

Corresponding author(s): Geltrude Mingrone

Last updated by author(s): Oct 24, 2022

Reporting Summary

Nature Portfolio wishes to improve the reproducibility of the work that we publish. This form provides structure for consistency and transparency in reporting. For further information on Nature Portfolio policies, see our Editorial Policies and the Editorial Policy Checklist.

Statistics

For all statistical analyses, confirm that the following items are present in the figure legend, table legend, main text, or Methods section.

n/a	Confirmed	
☐	☒	The exact sample size (*n*) for each experimental group/condition, given as a discrete number and unit of measurement
☐	☒	A statement on whether measurements were taken from distinct samples or whether the same sample was measured repeatedly
☐	☒	The statistical test(s) used AND whether they are one- or two-sided<br>*Only common tests should be described solely by name; describe more complex techniques in the Methods section.*
☒	☐	A description of all covariates tested
☐	☒	A description of any assumptions or corrections, such as tests of normality and adjustment for multiple comparisons
☐	☒	A full description of the statistical parameters including central tendency (e.g. means) or other basic estimates (e.g. regression coefficient) AND variation (e.g. standard deviation) or associated estimates of uncertainty (e.g. confidence intervals)
☐	☒	For null hypothesis testing, the test statistic (e.g. *F*, *t*, *r*) with confidence intervals, effect sizes, degrees of freedom and *P* value noted<br>*Give P values as exact values whenever suitable.*
☒	☐	For Bayesian analysis, information on the choice of priors and Markov chain Monte Carlo settings
☒	☐	For hierarchical and complex designs, identification of the appropriate level for tests and full reporting of outcomes
☒	☐	Estimates of effect sizes (e.g. Cohen's *d*, Pearson's *r*), indicating how they were calculated

Our web collection on statistics for biologists contains articles on many of the points above.

Software and code

Policy information about availability of computer code

Data collection	Multiskan™ GO Microplate Spectrophotometer (Thermo Fischer Scientific, Waltham, MA)<br>Luminex™ 200™ Instrument System and Bio-Plex Manager Software (Bio-Rad Laboratories, Hercules, CA)<br>Nikon Eclipse e200 (Nikon, Tokyo, J)<br>Chemidoc XRS Image system and Image Lab 5.0 software (Bio-Rad Laboratories, Hercules, CA)<br>CFX96 Touch Real-Time PCR Detection System<br>CFX Maestro Software 2.0 (Bio-Rad Laboratories, Hercules, CA)<br>CytoFlex and Kaluza software 2.1 (Beckman Coulter, Brea, CA)<br>Nikon A1 RHD25 and NIS-Elements imaging software (Nikon, Tokyo, J)<br>FACSAria (BD Biosciences, Franklin Lakes, NJ)<br>IVIS Spectrum (Perkin Elmer, Waltham, MA)<br>Bruker SPR-32pro (Bruker Daltonics SPR, Hamburg, Germany)
Data analysis	R environment (version 4.0.3)<br>SPSS version 26 software (SPSS Inc., Chicago, IL, USA)

For manuscripts utilizing custom algorithms or software that are central to the research but not yet described in published literature, software must be made available to editors and reviewers. We strongly encourage code deposition in a community repository (e.g. GitHub). See the Nature Portfolio guidelines for submitting code & software for further information.

Data

Policy information about availability of data

All manuscripts must include a data availability statement. This statement should provide the following information, where applicable:

- Accession codes, unique identifiers, or web links for publicly available datasets
- A description of any restrictions on data availability
- For clinical datasets or third party data, please ensure that the statement adheres to our policy

The authors declare that the data supporting the findings of this study are available within the paper and Supplementary Information. Source data are provided with this paper.

Field-specific reporting

Please select the one below that is the best fit for your research. If you are not sure, read the appropriate sections before making your selection.

☒ Life sciences ☐ Behavioural & social sciences ☐ Ecological, evolutionary & environmental sciences

For a reference copy of the document with all sections, see nature.com/documents/nr-reporting-summary-flat.pdf

Life sciences study design

All studies must disclose on these points even when the disclosure is negative.

Sample size	For all the experiments sample sizes were not predetermined.<br>Our sample size was based on similar experiments previously described in litterature (DOI: 10.1152/ajpgi.00357.2019) to give sufficient data values to conduct standard statistical test.
Data exclusions	No samples or animals were excluded fro the analyses.
Replication	All experiments were performed at least in duplicate.<br>All attempts at replication were successfuL
Randomization	All samples/animals were randomly allocated to experimental group and processed.
Blinding	Investigator were not blinded in cells or mice treatment. Blinding was not necessary because the results are quantitative and do not require subjective interpretation.

Reporting for specific materials, systems and methods

We require information from authors about some types of materials, experimental systems and methods used in many studies. Here, indicate whether each material, system or method listed is relevant to your study. If you are not sure if a list item applies to your research, read the appropriate section before selecting a response.

Materials & experimental systems

n/a	Involved in the study	
☐	☒	Antibodies
☒	☐	Eukaryotic cell lines
☒	☐	Palaeontology and archaeology
☐	☒	Animals and other organisms
☐	☒	Human research participants
☐	☒	Clinical data
☒	☐	Dual use research of concern

Methods

n/a	Involved in the study	
☒	☐	ChIP-seq
☐	☒	Flow cytometry
☒	☐	MRI-based neuroimaging

Antibodies

Antibodies used

The following antibodies were used in the study; they are listed as protein name, antigen , fluorophore (were applicable) followed by catalog number and supplier.

HSP70 (3A3): sc-32239; Santa Cruz Biotecnology (Dallas, TX)
GRP78 (76-E6): sc-13539; Santa Cruz Biotecnology (Dallas, TX)
normal mouse IgG: sc-2025; Santa Cruz Biotecnology (Dallas, TX)
Phospho-Akt (Ser473) (D9E) XP® Rabbit mAb #4060; Cell Signaling Technology (Danvers, MA)
Phospho-GSK-3α/β (Ser21/9) Antibody #9331; Cell Signaling Technology (Danvers, MA)

Phospho-FoxO1 (Thr24) Antibody #9464; Cell Signaling Technology (Danvers, MA)
Akt (pan) (C67E7) Rabbit mAb #4691; Cell Signaling Technology (Danvers, MA)
GSK-3α/β (D75D3) Rabbit mAb #5676; Cell Signaling Technology (Danvers, MA)
FOXO1 (C-9): sc-374427; Santa Cruz Biotecnology (Dallas, TX)
Glut4 (IF8): sc-53566; Santa Cruz Biotecnology (Dallas, TX)
Actin (C-2): sc-8432; Santa Cruz Biotecnology (Dallas, TX)
Alpha-Smooth Muscle Actin Monoclonal Antibody (1A4), eFluor 570, eBioscience™ #41-9760-82;Thermo Fisher scientific, (Waltham, MA)
COL1A1 Antibody (PA5-29569) Thermo Fisher scienGoat Anti-Rabbit IgG H&L (HRP) (ab6721)tific, (Waltham, MA)
Goat anti-Rabbit IgG (H+L) Highly Cross-Adsorbed Secondary Antibody, Alexa Fluor Plus 488 Thermo Fisher scientificCat # A-11008; (Waltham, MA)
Integrin beta4 (B-7) FITC: sc-514426; Santa Cruz Biotecnology (Dallas, TX)
Claudin-4 (A-12): Alexa Fluor 647: sc376643;Santa Cruz Biotecnology (Dallas, TX)
Phospho-PERK (Thr980) (16F8) Rabbit mAb #3179;Cell Signaling Technology (Danvers, MA)
Phospho-IRE1 Ser724 (ab48187);Abcam (Cambridge, UK)
ATF6 (F-7): sc-166659;Santa Cruz Biotecnology (Dallas, TX)
PERK (C33E10) Rabbit mAb #3192;Cell Signaling Technology (Danvers, MA)
IRE1 (14C10) Rabbit mAb #3294;Cell Signaling Technology (Danvers, MA)
Goat Anti-Mouse IgG H&L (HRP) (ab97023);Abcam (Cambridge, UK)
Goat Anti-Rabbit IgG H&L (HRP) (ab6721)

Validation

All antibodies are well validated by the manufacturers for identification of antigens, flow cytometry, western blot applications, as described on the manufacturers' websites.

Animals and other organisms

Policy information about studies involving animals; ARRIVE guidelines recommended for reporting animal research

Laboratory animals

One-hundred eight male Wistar rats, aged 8–10 weeks.
Ten male Obese fa/fa Zucker Diabetic Fatty rats (ZDF), aged 6-8-weeks

Wild animals

The study did not involved wild animals.

Field-collected samples

No field collected samples were used in the study.

Ethics oversight

All animal procedures were approved by the Catholic University of Rome Institutional Animal Care Committee.

Note that full information on the approval of the study protocol must also be provided in the manuscript.

Human research participants

Policy information about studies involving human research participants

Population characteristics

Inclusion Criteria:
Subjects of both sex (25 Years to 65 Years)
Subjects with NASH documented by liver biopsy and no evidence of another form of liver disease with a BMI ≥ 30 and ≤55 kg/m2.
Subjects with normal liver who underwent laparoscopic elective cholecystectomy, but otherwise in healthy conditions, will be used as controls for the discovery of non-invasive biomarkers.

Exclusion Criteria:

Coronary event or procedure (myocardial infarction, unstable angina, coronary artery bypass, surgery or coronary angioplasty) in the previous 6 months; Liver cirrhosis; End stage renal failure; Participation in any other concurrent therapeutic clinical trial; Any other life-threatening, non-cardiac disease; Pregnancy; Inability to give informed consent; Substantial alcohol consumption (>20 g/day for women or >30 g/day for men); Wilson's disease; Lipodystrophy; Parenteral nutrition; Abetalipoproteinemia; Interfering medications (e.g., amiodarone, methotrexate, tamoxifen, corticosteroids).

Recruitment

Patients were recruited from the Outpatient Clinics and Day Hospital of Obesity of the Catholic University of Rome, Italy.The study was approved by the Ethical Committee of the aCatholic University of the Sacred Heart in accordance with the guidelines of the National Health Ministry and the Helsinki Declaration, as revised in 2000. All participants provided written informed consent to participate in the study. Additional written informed consent was obtained prior to the intervention.

Ethics oversight

Catholic University of the Sacred Heart

Note that full information on the approval of the study protocol must also be provided in the manuscript.

Clinical data

Policy information about clinical studies

All manuscripts should comply with the ICMJE guidelines for publication of clinical research and a completed CONSORT checklist must be included with all submissions.

Clinical trial registration	NCT03524365; NCT04677101.
Study protocol	ClinicalTrials.gov
Data collection	The study population was recreutied between Decmber 2018 and July 2020.<br>Patients were recruited from the Outpatient Clinics and Day Hospital of Obesity of the Catholic University of Rome, Italy.
Outcomes	The primary aim of our study is to assess the effects of bariatric-metabolic surgery on NASH at 1 year after the interventions using liver histology.<br>Secondary aims are to assess the safety of bariatric surgery and the improvement of liver fibrosis by histological assesment, CVD by the Framingham risk score, insulin sensitivity by euglycemic clamp, T2DM by glycated hemoglobin , lipoprotein profile by lipidomic, NASH markers at 1 year and the follow up of NASH markers up to 2 years by Blood-based biomarkers screening.

ChIP-seq

Data deposition

☐ Confirm that both raw and final processed data have been deposited in a public database such as GEO.

☐ Confirm that you have deposited or provided access to graph files (e.g. BED files) for the called peaks.

Data access links<br>*May remain private before publication.*	*For "Initial submission" or "Revised version" documents, provide reviewer access links. For your "Final submission" document, provide a link to the deposited data.*
Files in database submission	*Provide a list of all files available in the database submission.*
Genome browser session<br>(e.g. UCSC)	*Provide a link to an anonymized genome browser session for "Initial submission" and "Revised version" documents only, to enable peer review. Write "no longer applicable" for "Final submission" documents.*

Methodology

Replicates	*Describe the experimental replicates, specifying number, type and replicate agreement.*
Sequencing depth	*Describe the sequencing depth for each experiment, providing the total number of reads, uniquely mapped reads, length of reads and whether they were paired- or single-end.*
Antibodies	*Describe the antibodies used for the ChIP-seq experiments; as applicable, provide supplier name, catalog number, clone name, and lot number.*
Peak calling parameters	*Specify the command line program and parameters used for read mapping and peak calling, including the ChIP, control and index files used.*
Data quality	*Describe the methods used to ensure data quality in full detail, including how many peaks are at FDR 5% and above 5-fold enrichment.*
Software	*Describe the software used to collect and analyze the ChIP-seq data. For custom code that has been deposited into a community repository, provide accession details.*

Flow Cytometry

Plots

Confirm that:

☒ The axis labels state the marker and fluorochrome used (e.g. CD4-FITC).

☒ The axis scales are clearly visible. Include numbers along axes only for bottom left plot of group (a 'group' is an analysis of identical markers).

☒ All plots are contour plots with outliers or pseudocolor plots.

☒ A numerical value for number of cells or percentage (with statistics) is provided.

Methodology

Sample preparation	To assess lipid droplet accumulation, primary hepatocytes were stimulated with complete DMEM medium supplemented with HSP70 (15 ng/ml) or GRP78 (3 ng/ml) or Oleic Acid (400 µmol/L) for 24 hours. To test the effect of TLR4 inhibition, cells

	were pretreated with or without TAK-242 (200 nM) for one hour. After the stimulation, half of the cells were stained with Nile Red (100 ng/mL) for 45 minutes.<br><br>HSCs activation was assessed by flow cytometry.<br>Cells were fixed and permeabilzed and stained for 15 minutes with alpha-smooth muscle Actin (α-SMA) and Collagen type I alpha 1 (COL1A1). Cells were washed and COLA1A was conjugated with alexafluor 488 secondary antibody.
Instrument	CytoFlex (Beckman Coulter, Brea, CA)
Software	Kaluza software (Beckman Coulter, Brea, CA).
Cell population abundance	No sorting was performed in this study
Gating strategy	Fluorescence target channels were met for all channels by using CytoFLEX Daily QC beads and the acquisition settings were defined by using VersaComp Antibody Capture Kit.

☒ Tick this box to confirm that a figure exemplifying the gating strategy is provided in the Supplementary Information.

Magnetic resonance imaging

Experimental design

Design type	*Indicate task or resting state; event-related or block design.*
Design specifications	*Specify the number of blocks, trials or experimental units per session and/or subject, and specify the length of each trial or block (if trials are blocked) and interval between trials.*
Behavioral performance measures	*State number and/or type of variables recorded (e.g. correct button press, response time) and what statistics were used to establish that the subjects were performing the task as expected (e.g. mean, range, and/or standard deviation across subjects).*

Acquisition

Imaging type(s)	*Specify: functional, structural, diffusion, perfusion.*
Field strength	*Specify in Tesla*
Sequence & imaging parameters	*Specify the pulse sequence type (gradient echo, spin echo, etc.), imaging type (EPI, spiral, etc.), field of view, matrix size, slice thickness, orientation and TE/TR/flip angle.*
Area of acquisition	*State whether a whole brain scan was used OR define the area of acquisition, describing how the region was determined.*

Diffusion MRI ☐ Used ☐ Not used

Preprocessing

Preprocessing software	*Provide detail on software version and revision number and on specific parameters (model/functions, brain extraction, segmentation, smoothing kernel size, etc.).*
Normalization	*If data were normalized/standardized, describe the approach(es): specify linear or non-linear and define image types used for transformation OR indicate that data were not normalized and explain rationale for lack of normalization.*
Normalization template	*Describe the template used for normalization/transformation, specifying subject space or group standardized space (e.g. original Talairach, MNI305, ICBM152) OR indicate that the data were not normalized.*
Noise and artifact removal	*Describe your procedure(s) for artifact and structured noise removal, specifying motion parameters, tissue signals and physiological signals (heart rate, respiration).*
Volume censoring	*Define your software and/or method and criteria for volume censoring, and state the extent of such censoring.*

Statistical modeling & inference

Model type and settings	*Specify type (mass univariate, multivariate, RSA, predictive, etc.) and describe essential details of the model at the first and second levels (e.g. fixed, random or mixed effects; drift or auto-correlation).*
Effect(s) tested	*Define precise effect in terms of the task or stimulus conditions instead of psychological concepts and indicate whether ANOVA or factorial designs were used.*

Specify type of analysis: ☐ Whole brain ☐ ROI-based ☐ Both

Statistic type for inference (See Eklund et al. 2016)	*Specify voxel-wise or cluster-wise and report all relevant parameters for cluster-wise methods.*

Correction | *Describe the type of correction and how it is obtained for multiple comparisons (e.g. FWE, FDR, permutation or Monte Carlo).*

Models & analysis

n/a	Involved in the study
☐	☐ Functional and/or effective connectivity
☐	☐ Graph analysis
☐	☐ Multivariate modeling or predictive analysis

Functional and/or effective connectivity | *Report the measures of dependence used and the model details (e.g. Pearson correlation, partial correlation, mutual information).*

Graph analysis | *Report the dependent variable and connectivity measure, specifying weighted graph or binarized graph, subject- or group-level, and the global and/or node summaries used (e.g. clustering coefficient, efficiency, etc.).*

Multivariate modeling and predictive analysis | *Specify independent variables, features extraction and dimension reduction, model, training and evaluation metrics.*
